# “I probably shouldn’t go in today”: Inequitable access to paid sick leave and its impacts on health behaviors during the emergence of COVID-19 in the Seattle area

**DOI:** 10.1371/journal.pone.0307734

**Published:** 2024-09-10

**Authors:** Chidozie D. Iwu, Sarah N. Cox, Sarah L. Sohlberg, Ashley E. Kim, Jennifer Logue, Peter D. Han, Thomas R. Sibley, Misja Ilcisin, Kairsten A. Fay, Jover Lee, Denise J. McCulloch, Yongzhe Wang, Michael Boeckh, Janet A. Englund, Lea M. Starita, Anjum Hajat, Helen Y. Chu

**Affiliations:** 1 Department of Medicine, University of Washington, Seattle, Washington, United States of America; 2 Department of Epidemiology, University of Washington, Seattle, Washington, United States of America; 3 Brotman Baty Institute for Precision Medicine, Seattle, Washington, United States of America; 4 Department of Genome Sciences, University of Washington, Seattle, Washington, United States of America; 5 Fred Hutchinson Cancer Center, Seattle, Washington, United States of America; 6 Department of Surgery and Population Sciences, City of Hope Comprehensive Cancer Center, Duarte, CA, United States of America; 7 Seattle Children’s Research Institute, Seattle, Washington, United States of America; 8 Department of Pediatrics, University of Washington, Seattle, Washington, United States of America; International Medical University, MALAYSIA

## Abstract

This study examines inequities in access to paid sick leave (PSL) by race/ethnicity, income, and sex and the role of PSL access on leave-taking and care-seeking behaviors among Seattle-area workers in the months leading up to and during the emergence of COVID-19 in the region. Survey responses were collected online and in-person from individuals experiencing acute respiratory illness symptoms between November 2019 and March 2020 as part of a community-based respiratory viral surveillance study. Chi-square tests and log-binomial models were used to assess the association between PSL access and various socioeconomic indicators. A total of 66.6% (n = 2,276) respondents reported access to PSL. Proportionally, access to PSL was highest in respondents identifying as Asian (70.5%), followed by White (68.7%), Latine (58.4%), Multiracial (57.1%), Black (47.1%), and Other (43.1%). Access to PSL increased with household income. Eighty three percent of high-income respondents reported access compared to 52.9% of low-income households. Only 23.3% of the lowest-income households reported access to PSL. Fewer females (65.2%) than males (70.7%) reported access to PSL. Access to PSL is inequitably distributed across income, race/ethnicity, and sex. This study reinforces the vast body of knowledge on how socioeconomic inequalities increase individual and community-level vulnerability to the impacts of infectious disease outbreaks. It also supports the role of labor and economic policy in mitigating (or exacerbating) these impacts. Exemplified by the COVID-19 pandemic, universal access to PSL, especially for marginalized populations, benefits all.

## 1 Introduction

The COVID-19 pandemic illuminated the social, economic, and health inequities faced by Black, Indigenous, and people of color (BIPOC) in the United States (U.S.). Structural racism and classism in healthcare, social, economic, and political systems have led to low-income, causing the BIPOC individuals to bear a disproportionate share of COVID-19 cases and deaths [1]. Policies created without an equity lens disproportionately harm some, while upholding the economic and social power of others. Labor policy encompasses a wide range of regulations and mandates (or lack thereof) with considerable downstream health effects [2]. One such contributing factor is the ability to miss work due to illness.

Paid sick leave (PSL) is widely regarded as essential for protecting the health of workers and their families [3]. Even so, the U.S. is one of the few high-income countries with no national PSL mandate [4]. Additionally, about half of all Americans rely on employer-provided insurance coverage for their healthcare [5]. As a result, many facets of preventive health and healthcare access are determined by one’s status as an employee. In a nation where healthcare costs are already prohibitive to accessing care [6], the added prospect of wage or job loss due to illness is untenable for many.

The ability to take PSL is important for several reasons. First, going to work while sick can put others’ health at risk. Being at work while contagious increases the risk of transmission in the workplace, as evidenced by several studies of influenza-like-illness, H1N1, and COVID-19 [7–10]. Access to PSL has been linked to changes in care-seeking behaviors such as increased usage of preventive health services [11, 12] and overall lower mortality [13]. Without access to PSL, workers are less likely to seek care or treatment for their illness or injuries, prolonging recovery and decreasing productivity [14].

According to the Bureau of Labor Statistics (BLS), the proportion of workers in the U.S. with PSL access has remained relatively constant over the past decade [15]. As a result, there is a gap in our understanding on how sick leave access may have changed, especially since the beginning of the COVID-19 pandemic. The primary objective of this study is to address this gap by assessing the sociodemographic disparity in access to PSL, focusing on race/ethnicity, annual household income, and sex within a community-based sample. The secondary objective is to examine how access to PSL may influence leave-taking and care-seeking behaviors within this population.

## 2. Methods

### 2.1 Data collection

This study uses data from the Seattle Flu Study (SFS), a multi-arm, cross-sectional respiratory virus surveillance study conducted in Washington state during the influenza season [16]. This analysis includes data from 2019–2020 in two arms of SFS: “Swab-and-Send,” [17] in which participants enrolled at home and returned self-collected swabs in the mail, and “Kiosk” enrollments [15], in which participants enrolled at community kiosks staffed by research assistants throughout the city. The study participants were enrolled from 2019-10-01 through 2020-03-30. Written informed consent was obtained from the study participants online through REDCap and in person. The study was approved by the University of Washington’s Institutional Review Board.

### 2.2 Study population

People with influenza-like illness (ILI) were eligible to enroll in the parent study. For the current study, participants must have completed their full enrollment questionnaires, be 18 years or older, and be employed.

### 2.3 Study setting

For the kiosk arm, data collection sites were set up at various public locations in King County, WA. Eligible and interested participants were able to consent, enroll, and have a nasal swab collected by trained study personnel onsite. For the Swab-and-Send arm, all questionnaires (Appendices A-D in S1 File) were completed online. For both arms of the study, detailed specimen collection and laboratory pathogen testing methods were conducted as described previously [18].

### 2.4 Measures

Primary exposures of interest are annual household income, race/ethnicity, and sex. All measures in this analysis were self-reported through online or in-person questionnaires. Race was self-reported based on U.S. Office of Management and Budget categories [18] and respondents were able to select all that apply (Appendix B n S1 File). Ethnicity was assessed by asking respondents if they identified as Hispanic/Latino (hereto referred to as ‘Latine’). Those who selected ‘Yes’ to Latine ethnicity were not included in any other race categories. To obtain adequate sample size in each category, those who selected only ‘Native Hawaiian or Pacific Islander’, ‘American Indian or Alaska Native’, or ‘Other’ were combined into the ‘Other, Non-Latine’ group. In our study, race and ethnicity are social constructs that represent the long-standing discrimination in the labor market and the racialization of employment [19]. Participants chose the income range that best represented their household income (Appendix C in S1 File). To determine participant sex, respondents were able to choose “Male,” “Female,” “Other,” or “Prefer Not to Say.” The study did not collect data on participants’ gender identity. The outcome of interest, access to PSL, was self-reported in the initial questionnaire and defined as “Yes” or “No”.

### 2.5 Statistical analysis

A Pearson’s chi-square test of independence was performed to test whether there is a difference between race/ethnicity, household income, and sex, respectively with access to PSL. Additional chi-square tests were performed to test associations between access to PSL with leave-taking, flu vaccination, and care-seeking, respectively. For the combined race and ethnicity variable, those who responded with “Prefer not to say” were excluded from chi-square tests and regression models. Those who responded “Don’t know” or “Prefer not to say” to the annual household income question were also excluded. For lack of adequate sample size, those who responded “Other” or “Prefer not to say” for sex were excluded from the data analyses.

A multivariate log-binomial model was used to estimate the prevalence ratios between household income and access to PSL, adjusting for race/ethnicity, sex, education, and age. Given historical and continued labor market discrimination, household income and education are presumed to be on the causal pathway between both sex and PSL and race and ethnicity and PSL. Therefore, the associations between both variables and PSL were analyzed in a separate log-binomial model including only sex, race/ethnicity, and age as covariates. For each variable, the category with the largest number of participants was used as the reference category. Two tailed p-values and 95% confidence intervals were calculated for all analyses using 2-sided Wald tests. P-values were considered significant at an alpha level of 0.05. All analyses were performed using R version 3.6.3 [20].

## 3. Results

There were 3,513 enrollments by participants who met inclusion criteria; of these, 97 individuals had multiple enrollment instances, resulting in 3,416 total participants included. Data from the individual’s first enrollment was included in this analysis. Eighty percent (n = 2,733) of the 3,416 participants completed the week-follow-up questionnaire and were included in the analysis of the associations between PSL with leave-taking and care-seeking behavior. Descriptive statistics of the study population are presented in Table 1. Overall, 66.6% (n = 2,276) of participants reported access to PSL.

**Table 1:** Sociodemographic characteristics of study sample, Washington, 2019–2020^†^.

	Access to PSL		Overall (N = 3,416)
	No (n = 1,140)	Yes (n = 2,276)	
**Age at Enrollment (years)**			
Median [Min, Max]	34.0 [18.0, 82.0]	36.0 [18.0, 73.0]	35.0 [18.0, 82.0]
**Sex**			
Female	788 (69.1%)	1,469 (64.5%)	2,257 (66.1%)
Male	334 (29.3%)	792 (34.8%)	1,126 (33.0%)
Other/Don’t Say	18 (1.6%)	15 (0.7%)	33 (1.0%)
**Race/Ethnicity**			
White	667 (58.5%)	1,461 (64.2%)	2,128 (62.3%)
Asian	205 (18.0%)	485 (21.3%)	690 (20.2%)
Black	48 (4.2%)	41 (1.8%)	89 (2.6%)
Latine	92 (8.1%)	130 (5.7%)	222 (6.5%)
Multiracial	68 (6.0%)	89 (3.9%)	157 (4.6%)
Other	60 (5.3%)	70 (3.1%)	130 (3.8%)
**Household Income Level**			
<25k	168 (14.7%)	51 (2.2%)	219 (6.4%)
25k-50k	178 (15.6%)	200 (8.8%)	378 (11.1%)
50k-75k	145 (12.7%)	266 (11.7%)	411 (12.0%)
75k-100k	142 (12.5%)	277 (12.2%)	419 (12.3%)
100k-125k	100 (8.8%)	277 (12.2%)	377 (11.0%)
125k-150k	65 (5.7%)	207 (9.1%)	272 (8.0%)
>150k	161 (14.1%)	791 (34.8%)	952 (27.9%)
Missing	181 (15.9%)	207 (9.1%)	388 (11.4%)
**Education Level**			
Less than high school	15 (1.3%)	7 (0.3%)	22 (0.6%)
High school/GED	107 (9.4%)	54 (2.4%)	161 (4.7%)
Some college	315 (27.6%)	264 (11.6%)	579 (16.9%)
Bachelor’s	369 (32.4%)	919 (40.4%)	1,288 (37.7%)
Advanced degree	309 (27.1%)	1,006 (44.2%)	1,315 (38.5%)
Prefer not to say	19 (1.7%)	25 (1.1%)	44 (1.3%)
Missing	6 (0.5%)	1 (0.0%)	7 (0.2%)
**Sought Care for Illness**			
No	468 (41.1%)	1,171 (51.4%)	1,639 (48.0%)
Yes	368 (32.3%)	698 (30.7%)	1,066 (31.2%)
Missing	304 (26.7%)	407 (17.9%)	711 (20.8%)
**Received Flu Vaccine**			
No	502 (44.0%)	728 (32.0%)	1,230 (36.0%)
Yes	638 (56.0%)	1,548 (68.0%)	2,186 (64.0%)
**Worked from Home due to Illness**			
No	624 (54.7%)	1,064 (46.7%)	1,688 (49.4%)
Yes	230 (20.2%)	987 (43.4%)	1,217 (35.6%)
Missing	286 (25.1%)	225 (9.9%)	511 (15.0%)
**Worked Fewer Hours due to Illness**			
No	505 (44.3%)	1,128 (49.6%)	1,633 (47.8%)
Yes	349 (30.6%)	923 (40.6%)	1,272 (37.2%)
Missing	286 (25.1%)	225 (9.9%)	511 (15.0%)
**Missed Work due to Illness**			
No	363 (31.8%)	904 (39.7%)	1,267 (37.1%)
Yes	491 (43.1%)	1,147 (50.4%)	1,638 (48.0%)
Missing	286 (25.1%)	225 (9.9%)	511 (15.0%)

^†^Numbers in parentheses show percentage of column total

### 3.1 Sociodemographic differences in access to paid sick leave

**Fig 1:**
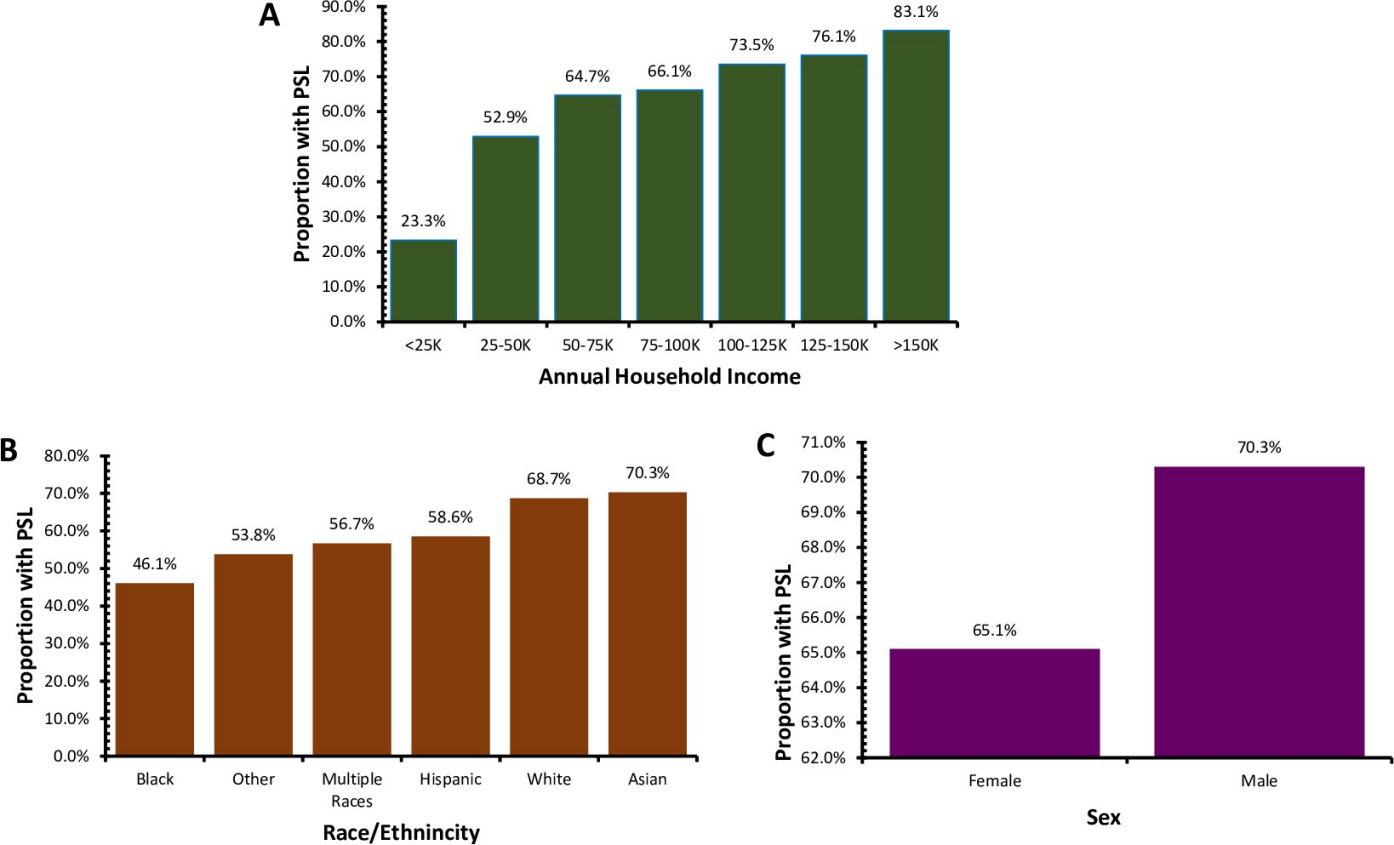
Access to PSL by (A) annual household income, (B) race and ethnicity, and (C) sex.

PSL access increased with each categorical increase in household income (Fig 1A) and differed significantly, χ^2^ (6, N = 3,028) = 349.9, p < .0001. Only 23.3% of those in the lowest annual household income category (“Less than or equal to $25,000”) had PSL, while 83.1% of those in the highest category had PSL. Reported access to PSL varied by race [χ^2^ (5, N = 3,416) = 53.3, p < .001] and was highest in the Asian group (70.5%), followed by White (68.7%), Latine (58.4%), two or more races (57.1%), Black (47.1%), and Other (43.1%) (Fig 1B). White participants comprised 64.2% of those with access to PSL, but only 58.5% of those without it. 65.2% of female respondents reported PSL, compared with 70.7% of male respondents, a statistically significant difference (χ^2^ (1, N = 3,383) = 9.9, p = 0.002 (Fig 1C).

**Fig 2:**
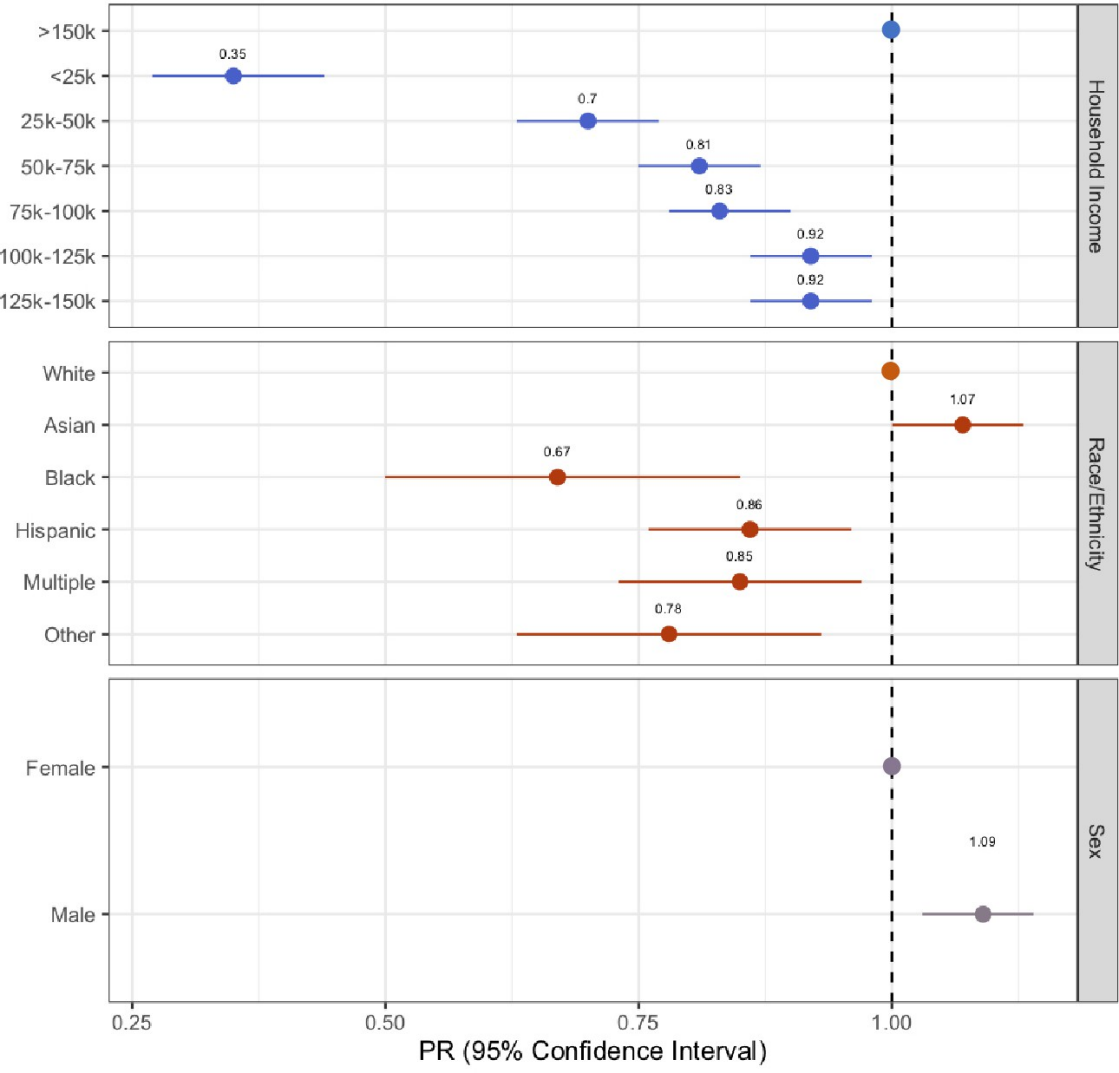
Forest plot showing adjusted prevalence ratios and 95% confidence intervals from the log binomial regression models. For race and ethnicity, White served as the reference category. For annual household income, over $150,000 per year was the reference category. For sex, female was the reference category. The dotted line represents the null value of 1.00.

Results from the log-binomial regression models are summarized in Fig 2 (S1 Table in S1 File). After adjusting for sex and age, access to PSL was 7% higher in Asian participants (PR: 1.07, 95% CI:1.00, 1.13), 33% lower among Black participants (PR: 0.67, 95% CI:0.50–0.85), 14% lower among Latine participants (PR:0.86, 95% CI:0.76–0.96), 15% lower among those who selected two or more race categories (PR: 0.85, 95% CI:0.73–0.97), and 22% lower among those who selected the “Other” race category (PR: 0.78, 95% CI:0.63–0.93) relative to White participants.

After adjusting for race/ethnicity and age, access to PSL among males was 9% higher than that of females (PR: 1.09, 95% CI:1.03–1.14). After adjusting for race/ethnicity, sex, age, and education, the probability of having PSL among those in the lowest income category was 65% lower than those in the highest income category (PR: 0.35, 95% CI:0.27–0.44). All income categories had statistically significant lower access to PSL relative to the highest income category.

### 3.2 Paid sick leave and care-seeking behavior

Overall, 79.2% of participants answered whether they sought clinical care for their current illness. A significant difference [χ^2^ (1, N = 3,416) = 47.5, p<0.001] was seen between those with access to PSL being 1.2 times more likely to have received the flu vaccine than those without (68.0% and 56.0%, respectively). However, a higher proportion of those without PSL who completed the follow-up questionnaire reported having sought care for their illness a week later; χ^2^ (6, N = 2,705) = 9.42, p = 0.001.

Eighty-five percent of participants answered the question of whether they had missed work or worked from home due to their illness. Missing work was similar between those with PSL (55.2% missed work, 40.9% worked fewer hours) and those without (54.2% missed work, 45.0% worked fewer hours). The association between missing a day of work and PSL was not statistically significant χ^2^ (1, N = 2,905) = 0.54, p = 0.462; however, working fewer hours was found to be associated with PSL χ^2^ (1, N = 2,905) = 4.02, p = 0.045. Participants who had PSL were more likely to have worked from home during their illness episode, χ^2^ (1, N = 2,905) = 110.36, p<0.001 (Table 1).

## 4. Discussion

Data included in this study were collected during the emergence of COVID-19 in the Seattle region and have implications for the increased vulnerability of workers who are not able to work remotely or take PSL during that time. Our results reinforce existing evidence that access to PSL is unequally distributed, with low-income, Black and Latine, and female individuals bearing the highest burden [13, 21]. A U.S. study using nationally-representative data from 2008–2016, showed that around 70% of civilian full-time workers had access to PSL, relative to about 20% for part-time workers [22]. In terms of racial disparities, this study concluded that Hispanic workers were less likely to have access to paid sick leave compared with White non-Hispanic workers, while there were no significant differences for Black non-Hispanic workers relative to Whites [15]. The relationships between health behaviors and access to PSL illustrate the impact of labor policy on individual health decisions and the importance of labor policy change as a tool for building community resilience to future infectious disease outbreaks.

Although those with and without access to PSL reported missing days of work due to their illness in similar proportions, incongruencies in remote work access and reduction of hours provide some insight into other employment factors that may have influenced these results. Those with PSL were 2.2 times more likely to work from home when sick than those without PSL. This provides evidence that those working in jobs without PSL may have been least able to work remotely, putting themselves and their families at higher risk during an infectious disease outbreak. As part of the federal government’s COVID-19 relief bill, the Families First Coronavirus Response Act, included a temporary provision for PSL for certain employers [23]. Even so, PSL has not been discussed widely in the literature as a contributing factor to economic and racial disparities in COVID-19 incidence and mortality.

This study found that 66.6% of participants reported access to PSL, a lower proportion than the BLS estimate of 76% of civilian workers [15]. Our findings that workers without PSL were less likely to have received a flu shot is consistent with prior findings from studies in the U.S. regarding preventive healthcare access and use [12, 24]. While other studies have reported an association between no PSL and delayed or forgone care [25], we observed higher reports of individuals seeking care for their illness a week later in those without PSL. This, combined with our results of those without PSL being less likely to work from home and less likely to reduce their hours while ill, could suggest worse health outcomes and further disease progression before implementing home or clinical care interventions. Especially, at a time when the study region was experiencing an unusually high burden of influenza B and the underlying emergence of SARS-CoV-2, both more virulent than what is typical of seasonal respiratory viruses.

### 4.1. Limitations

There were several limitations to this study. One main limitation is the convenience sampling strategy, which may have impacted the representativeness of the sample. The median household income in King County in 2018 was $95,009 [26]. In our study sample, the median household income category was between $100,001 to $125,000, indicating a, potentially, slightly higher average income among the study sample than the greater King County population. Additionally, median household income in the United States in 2019 was $68,703 [27]. These data indicate that the study population included here was significantly wealthier, on average, than the U.S. population.

Limited race and ethnicity categories and statistical power requirements meant that participants of many different racial and ethnic identities were combined, however, this is not meant to infer that all are equally affected by access to PSL. We also did not collect data on participant’s gender identity or include the “Other” sex category in analysis due to statistical power requirements. Research on disaggregated racial and ethnic data and the use of more discrete measures of social, cultural, and structural variables is needed to identify high-risk sub-groups and the risk/protective factors of intersectional identities.

This study had several potential unmeasured confounding variables. Illness severity and length, or biased completion of the follow-up survey could have impacted results on care-seeking and PSL access. Additional socioeconomic factors such as immigration status, occupation, industry, or part-time/full-time status, were not surveyed and so were not included in the analysis. Additionally, no information was collected on the type of PSL available to participants or on any employer specific PSL policies. For example, if an employee is required to find their own shift replacement, provide a doctor’s note, or if PSL is subtracted from other paid time off such as holidays or vacation.

### 4.2. Policy implications

Our findings show how current labor policy contributes to systemic racism, classism, and sexism, exacerbates health disparities, and puts the larger community at risk in the event of future infectious disease outbreaks. Empowering individuals to make better health decisions and working towards a more equitable healthcare system requires the expansion of PSL access to all workers, regardless of length of occupation, industry, full-time status, or salaried vs hourly. Doing so improves the impact of preventive care services such as flu vaccination, reduces infectious disease transmission, and protects economically vulnerable workers from job and wage loss in the event of illness.

## 5. Conclusion

We have shown that access to PSL is inequitably distributed across income, race/ethnicity, and sex as well as how PSL may contribute to differences in the utilization of preventative care, health behaviors, and care-seeking. Lack of access to PSL forces workers to choose between their health and wages and leads to compounding vulnerability for themselves and their communities. Future studies should investigate barriers in accessing PSL and it’s impact on healthcare-seeking behaviors using data collected from workers’ point-of-view using more nuanced analyses of socioeconomic variables and should examine the impact of changes in PSL policy on individual, workforce, and community-level clinical outcomes. Interdisciplinary multi-systems approaches are needed to improve community resilience to future disease outbreaks and address health inequities in the United States. Providing equitable and adequate PSL to all workers is a single step in the right direction.

## Supporting information

S1 File (DOCX)

S2 File (ZIP)
