# Supplementary material for: “I probably shouldn’t go in today”: Inequitable access to paid sick leave and its impacts on health behaviors during the emergence of COVID-19 in the Seattle area
Source: PLoS One. 2024 Sep 10;19(9):e0307734. doi: 10.1371/journal.pone.0307734 (PMC11386467; doi:10.1371/journal.pone.0307734)
Supplement: S1 File — (DOCX) [file pone.0307734.s001.docx]

**APPENDICES**

Appendix A: Consent Forms

- A1: Kiosk Consent Form
- A2: Swab-and-Send Consent Form

Appendix B: Enrollment Questionnaire

Appendix C: Illness Questionnaire

Appendix D: One Week Follow-Up Questionnaire

**Supplemental Methods**

The outcome of interest, access to PSL, was assessed with the question, “At my place of work, employees are encouraged to take time off or work from home if they are sick.” Respondents could answer with one of the following options: “I am not currently employed” (excluded), “Yes, and I would be paid for hours missed,” “Yes, but I would not be paid for hours missed,” or “No.” Those who answered with "Yes, but I would not be paid for hours missed" or "No" were categorized as not having access to PSL, while those who answered "Yes, and I would be paid for hours missed" were categorized as having access to PSL.

The secondary exposures, participants were asked, “Have you sought clinical care for your illness?” at the one-week follow-up survey. Swab-and-Send participants were asked the same question in both the enrollment questionnaire and the one-week follow-up survey. Receipt of flu vaccination, a form of preventive care-seeking, was assessed with the question, “Have you received this season's influenza (flu) vaccine?” Leave-taking was measured with the question: “Did any of the following occur because you were feeling sick?” Participants were able to select all that apply from “I missed work”, “I worked from home”, “I worked fewer hours than usual”, or “None of the above.”

All nasal swab samples were screened using a custom, TaqMan-based Open Array panel (Thermo Fisher) for multiple respiratory pathogens. See Kim et al (2021)^30^ for detailed pathogen testing methods.

**Supplemental Table 1:** Log binomial regression results^†^

|  | **PR^‡, §^** | **95% CI^‡^** |
| --- | --- | --- |
| **Household Income** |  |  |
| >150k | Ref**^‡^** | Ref**^‡^** |
| <25k | 0.35 | 0.27, 0.44 |
| 25k-50k | 0.7 | 0.63, 0.77 |
| 50k-75k | 0.81 | 0.75, 0.87 |
| 75k-100k | 0.83 | 0.78, 0.90 |
| 100k-125k | 0.92 | 0.86, 0.98 |
| 125k-150k | 0.92 | 0.86, 0.98 |
| **Race/Ethnicity** |  |  |
| White | Ref**^‡^** | Ref**^‡^** |
| Asian | 1.07 | 1.00, 1.13 |
| Black | 0.67 | 0.50, 0.85 |
| Latine | 0.86 | 0.76, 0.96 |
| Multiracial | 0.85 | 0.73, 0.97 |
| Other | 0.78 | 0.63, 0.93 |
| **Sex** |  |  |
| Female | Ref**^‡^** | Ref**^‡^** |
| Male | 1.09 | 1.03, 1.14 |
| ^†^421 participants had missing values in one or more of the covariate categories and were excluded from the models. | | |
| ^‡^PR=prevalence ratio. CI=confidence interval. Ref=reference category. | | |
| ^§^Income PRs are adjusted for race and ethnicity, sex, education and age. Sex PRs are adjusted for race and ethnicity and age (due to education and income on the causal pathway). Race/ethnicity PRs are adjusted for sex and age (due to education and income on the causal pathway). | | |
