# Supplementary material for: “I probably shouldn’t go in today”: Inequitable access to paid sick leave and its impacts on health behaviors during the emergence of COVID-19 in the Seattle area
Source: PLoS One. 2024 Sep 10;19(9):e0307734. doi: 10.1371/journal.pone.0307734 (PMC11386467; doi:10.1371/journal.pone.0307734)
Supplement: S2 File — (ZIP) [file pone.0307734.s002.zip › Copy of AppendixA1_SFS_Kiosk_Consent.pdf]

Seattle Flu Study Consent

UNIVERSITY OF WASHINGTON CONSENT FORM: Ages 18 and up ASSENT FORM: Ages 13-17 SEATTLE FLU STUDY Researchers: Helen Y. Chu, MD, MPH, Assistant Professor of Medicine (University of Washington)

Janet A. Englund, MD, Professor of Pediatric Infectious Diseases (Seattle Children's Hospital)

Michael Boeckh, MD, PhD, Professor of Medicine (Fred Hutchinson Cancer Research Center)

Contact: Izzy Brandstetter, MPH, Research Coordinator (University of Washington) (206) 221-4588

We are asking you to be in a research study. This form gives you information to help you decide whether or not to be in the study. Being in the study is voluntary. Please read this carefully. You may ask any questions about the study. Then you can decide whether or not you want to be in the study.

This form also serves as an assent form for individuals age 13-17. This means that if you choose to take part in this research study, you would sign this form to confirm your choice. Your parent or legally-authorized representative would also need to give their permission and sign this form for you to join the study.

This form is also used for parents and legally-authorized representatives of subjects to provide permission to the research team for individuals who are not capable of providing permission themselves. In such cases, the term "you" refers to the subject or your child.

Purpose of the Study Colds are common in children and adults, and can cause a runny nose, cough, or trouble breathing. We want to understand how people get sick with these cold germs and how they spread from person to person. Once we understand how the germs start and spread, we can have a better plan to prevent them from making people sick. We also want to provide peoiple with a way to see what germs are in their neighborhood. This information may be on the internet for everyone to see.

Seattle Flu Study researchers, Washington State Department of Health, and the Centers for Disease Control and Prevention (CDC) are closely monitoring an outbreak or respiratory disease. This outbreak is caused by a novel (new) coronavirus. Some person-to-person spread of this virus outside China has been detected. Because of your symptoms today, we would like to test to see if you (or your child) have an infection caused by this new coronavirus. This information will not be on the internet for everyone to see.

Study Procedures We are testing children and adults who might have a cold. We are inviting you to participate because you have the symptoms of a cold. This study involves:

- Answering questions about your health, who you are around, and your living situation. We think these quesitons will take about 15 minutes. You may choose not to answer any sensitive question that you do not want to.

- Doing a swab of your nose to look for germs. This will only take about 5 seconds.

- To test for novel coronavirus, the swab may be sent to a public health laboratory for testing with a test that was authorized for emergency use. If the test is positive, you may be contacted by your local health department. You might be asked to restrict your (your child's) movement. This may mean that you (your child) would need to stay away from other people until the infection has cleared. Also, you might be asked about who you (your child) has been in contact with since becoming ill. The people you (your child) has been in contact with might be contacted to see if they are ill too. You may also be asked to provide additional swabs for testing. You may not be contacted if the first test result is negative.

- Emailing you to ask questions about your cold in about 1 week.

- We may ask you to blow your nose into a tissue (like Kleenex) so we can compare the test results to the swab from your nose.

- If you are eligible and interested, we may test your nose swab for germs that can cause your cold symptoms on-site and reutn your results to you today. This is optional and you may opt out of having your sample tested on-site or receiving your results.

This study does not replace care you would receive from your doctor, and your participation is voluntary.

Risks, Stress, or Discomfort We do not expect serious side effects from this study. Getting the nasal swab may cause mild discomfort, watery eyes, or sneezing. Some of the questions that we might ask you are sensitive and may make you feel uncomfortable. You do not have to answer any question that you do not want to. There is a risk that your privacy could be breached. We will do everything that we can to make sure that this does not happen.

Benefits of the Study This study will not benefit you directly. It may help others in the future by learning what germs make people sick. We hope that the neighborhood map helps you understand what germs are in your community. Results from the novel coronavirus test could help identify spread of the virus in the community.

Source of Funding The University of Washington is receiving financial support from an anonymous donor.

Confidentiality of Research Information Your record will be kept secret. You will not be identified in any report about this study. Your study record will never be used against you.

The information we collect from you as well as the germs that make you sick will be on a public website to show the spread of germs in your community. This information will not be shared in a way that will reveal your identity.

The persons involved in these novel coronavirus testing activities and the state or local health department will know about your personal (your child's) information and the results of testing. Beyond this, your name (and your child's name) will be kept confidential and will not be shared.

Government and university staff sometimes review studies such as this one to make sure they are being done safely and legally. If a review of this study takes place, your records may be examined. The reviewers will protect your privacy. The study records will never be used against you.

Washington State law requires that we report certain health conditions to local public health jurisdictions or the Department of Health. If your sample tests positive for one of these conditions, a local health jurisdiction or the Department of Health might contact you with more questions. They will keep your information secret.

Use of Information and Specimens Characterizing Germs

We will characterize the germs that are making you sick and share information about them. Information that identifies you would not be shared on the internet.

Returning Results to You

You will receive a link to a website where you can view the results of your nose swab test within three months of when the swab is collected. You can enter your nose swab barcode to view your results for influenza (flu) and Respiratory Syncytial Virus (RSV) testing when they are ready. The results may tell you what germs are making you sick, how they are characterized, and advice on how to keep other people from getting sick. You are not required to go to the website to look at your results if you do not want to. If you do not have access to the internet and would like to know your results, you may bring your barcode to one of our community kiosks, or call 206-221-4588. We may

not be able to give you your results if you lose your barcode.

Using Your Data in Future Research

Storing samples so researchers can use them in the future is called "banking." Researchers also bank information and samples so they can share it with other researchers.

The information and/or samples that we obtain from you for this study might be used for future studies. We may remove anything that might identify you from the information and samples. If we do so, that information and samples may then be used for future research studies or given to another researcher without getting additional permission from you. It is also possible that in the future we may want to use or share study information that might identify you. If we do, a review board will decide whether or not we need to get additional permission from you. It is possible the information will be used in future public health investigations. However, these will NOT include human genetic analysis.

By signing this consent form, you are giving your permission for these researchers to use your sample for future use.

Commercial Profit

Your data and/or samples may be used to make new products, tests, or findings. These may have value and may be developed and owned by the research team and/or others. If this happens, there are no plans to pay you.

Other Information You may refuse to participate and you are free to leave this study at any time without penalty or loss of benefits to which you are otherwise entitled (such as your normal medical care). Please note you have the option to accept or refuse this novel coronavirus testing.

By signing this form, you do not give up any of your rights.

If you join this study, you will receive an up to $10 gift card for each nose swab that we collect. You will receive the gift card the same day that we collect the sample.

Research-Related Injury If you think you have been harmed from being in this research, contact Izzy Brandstetter at (206) 221-4588. The University of Washington does not normally provide compensation for harm except through its discretionary program for medical industry. However, the law may allow you to seek other compensatio if the harm is the fault of the researchers. You do not waive any right to seek payment by signing this consent form.

Subject's Statement This study has been explained to me. I volunteer to take part in this research. I have had a chance to ask questions. If I have questions later about the research, or if I have been harmed by participating in this study, I can contact one of the researchers listed onthe first page of this consent form. If I have any questions about my rights as a research subject, I can call the Human Subjects Division at (206) 543-0098 or call collect at (206) 221-5940. I will receive a copy of this consent form.

UNIVERSIDAD DE WASHINGTON
FORMULARIO DE CONSENTIMIENTO: Edades 18 y más
FORMULARIO DE ACEPTACIÓN: Edades 13-17
ESTUDIO DE LA INFLUENZA EN SEATTLE

Investigadores:
Dra. Helen Y. Chu, Profesora Asistente de Medicina (Universidad de Washington)
Dra. Janet. A Englund, Profesora de Enfermedades Infecciosas Pediátricas (Hospital de Niños de Seattle)
Dr. Michael Boeckh, Profesor de Medicina (Centro de Investigación para el Cáncer Fred Hutchinson)

Contacto:
Izzy Brandstetter, MPH, Coordinadora de Investigación (Universidad de Washington) (206) 221-4588

Le pedimos participar en este estudio de investigación. Este formulario le brinda la información para ayudarle a decidir si desea o no participar en el estudio. Estar en el estudio es voluntario. Favor de leer esto cuidadosamente. Usted puede hacer preguntas acerca de este estudio. Después puede decidir si desea o no participar en el estudio. Este formulario también sirve como un formulario de acuerdo para las personas de 13-17 años. Esto significa que si elige participar en este estudio de investigación, usted firmará este formulario para confirmar su elección. Su padre o representante legalmente autorizado también necesitará dar su permiso y firmar este formulario para que usted se incorpore al estudio.
Este formulario también es utilizado por los padres y representantes legalmente autorizados del participante para dar permiso al equipo de investigación por los participantes que no son competentes para dar autorización ellos mismos. En dichos casos, el término “usted” se refiere al participante o su hijo.

PROPÓSITO DEL ESTUDIO

La gripe es común en los niños y adultos, y puede causar secreción nasal, tos o dificultades para respirar. Queremos comprender cómo las personas se enferman con estos gérmenes de la gripe y cómo ellos se propagan de persona a persona. Una vez que comprendamos como los gérmenes comienzan y se propagan, podremos tener un mejor plan para prevenir que ellos enfermen a las personas. También queremos proporcionar a las personas una forma de ver qué gérmenes hay en su vecindario. Esta información puede estar en el internet para que todos la vean.

Investigadores del Estidio de la Influenza en Seattle, el Departmento de Salud del Estado de Washington, y los Centros de Control y Prevención de Enfermedades (CDC) están monitoreando cerca de un brote de una enfermedad respiratoria. Este brote es causa por el nuevo coronavirus. Alguna propagación de esta enfermedad entre personas a fuera de China ha sido detectada. Debido a sus síntomas de hoy, nos gustaría ver si usted (o su hijo) tiene una infección causada por este virus nuevo. Esta información no estará en el internet para que todos la vean.

PROCEDIMIENTOS DEL ESTUDIO

Estamos evaluando a niños y adultos que podrían tener una gripe. Estamos invitándole a participar porque tiene los síntomas de una gripe. Este estudio involucra:

· Contestar preguntas acerca de su salud, con quién está y su situación de vida. Pensamos que estas preguntas tardarán unos 15 minutos. Puede optar no contestar a ninguna pregunta delicada que no desee contestar.

· Tomar una muestra nasal con hisopo para buscar gérmenes. Esto solo tardará unos 5 segundos.

· Para probar para el nuevo coronavirus, el hisopo puede ser enviado a un laboratorio de salud pública. Si la prueba es positiva, puede estar contactado por el departamento local de la salud pública. Se le puede pedir que restrinja su (o su hijo) movimiento. Esto puede significar que usted (o su hijo) necesitaría mantenerse alejado de ostras personas hasta que la infección haya desaparecido. Además, es posible que le preuntarán sobre con quien ha estado en contacto desde que se enfermó. Las personas con quien usted (o su hijo) ha estado en contacto pueden ser contactados para ver si también está enfermos. También se le puede pedir que proporcione hisopos adicionales para probar. Es posible que no le contactemos si el primer resultado de la prueba es negativo.

· Enviarle un correo electrónico con preguntas acerca de su gripe en aproximadamente 1 semana.

· Podemos pedirle también que se suene la nariz en un pañuelo (como un kleenex) para que podamos comparar el resultado de la prueba con el del hisopo tomado a su nariz.

· Si está elegible e interesado, podemos analizar su hisopo nasal en busca de los gérmenes que pueden causar sus síntomas de resfriado en el sitio y darle los resultados hoy mismo. Esto es opcional y puede optar por que su muestra no sea analizada en ese momento en el sitio o recibir sus resultados.

Este estudio no reemplaza el cuidado que recibe de su médico, y su participación es voluntaria.

RIESGOS, ESTRÉS O INCOMODIDAD

No esperamos efectos secundarios de gravedad de este estudio. Obtener la muestra del hisopo nasal puede causar una molestia leve, ojos llorosos, o estornudos. Algunas de las preguntas que podríamos hacer son delicadas y puede hacerle sentir incómodo. No tiene que contestar ninguna pregunta que no desee. Existe el riesgo de que su privacidad se viole. Haremos todo lo posible para asegurarnos que esto no ocurra.

BENEFICIOS DEL ESTUDIO

Este estudio no le beneficiará directamente. Puede ayudar a otros en un futuro a conocer que gérmenes enferman a las personas. Esperamos que el mapa de su vecindario le ayude a comprender que gérmenes hay en su comunidad. Resultados de la prueba de coronavirus nuevo pudieran ayudar a identificar la propagación del virus en la comunidad.

FUENTE DE FINANCIACIÓN

La Universidad de Washington está recibiendo apoyo financiero de un donante anónimo.

CONFIDENCIALIDAD DE LA INFORMACIÓN DE LA INVESTIGACIÓN

Su expediente se mantendrá en secreto. No será identificado en ningún reporte acerca del estudio. Su expediente nunca será usado en su contra.

La información que recopilamos de usted al igual que los gérmenes que lo han enfermado estará en un sitio web público para mostrar como los gérmenes se propagan en su comunidad. Esta información no será compartida de ninguna manera en la cual se revelé su identidad.

Las personas involucradas en estas actividades de pruebas para el nuevo coronavirus y el departmento de salud del estado o local sabrán sobre su información personal (la de su hijo) y los resultados de las pruebas. Más allá de esto, su nombre (o lo de su hijo) mantendrá confidencial y no se compartirá.

El personal del gobierno y de la Universidad a veces revisa los estudios como este para asegurarse que se realicen

de manera segura y legal. Si se realiza una revisión de este estudio, sus registros pueden ser examinados. Los revisores protegerán su privacidad. Los expedientes del estudio nunca serán usados en su contra.

La ley del estado de Washington requiere que reportemos ciertas condiciones a las jurisdicciones locales de salud pública o al Departamento de Salud. Si su muestra da positiva por alguna de estas condiciones, una jurisdicción local de salud o el Departamento de Salud puede contactarle con más preguntas. Ellos mantendrán su información en secreto.

USO DE LA INFORMACIÓN Y ESPECÍMENES

Caracterizando Gérmenes

Caracterizaremos los gérmenes que lo enferman y compartiremos la información acerca de ellos. La información que le identifica no será compartida en el internet.

Haciéndole saber los resultados

Recibirá un enlace al sitio web donde podrá ver los resultados de la prueba de hisopo nasal dentro de tres meses posteriores a la recolección del hisopo. Puede ingresar el código de barras para ver sus resultados para las pruebas de la influenza (flu) y el virus respiratorio sincitial (VRS) cuando estén listos. Los resultados pueden decirle que gérmenes lo están enfermando, como están caracterizados, y consejos de cómo evitar que otras personas se enfermen. No es necesario que vaya al sitio web y vea sus resultados si no lo quiere hacer. Si no tiene acceso al internet y le gustaría saber los resultados, puede traer su código de barras a uno de los quioscos comunitarios, o llamar al (206) 221-4588. Es posible que no podamos darle los resultados si pierde su código de barras.

Usando sus datos en investigaciones futuras.

El almacenamiento de muestras para que los investigadores puedan usarlas en el futuro se llama “banco” Los investigadores también guardan la información y muestras para poder compartirla con otros investigadores.

La información y/o muestras que son obtenidas de usted para este estudio pueden ser usadas para estudios futuros. Podríamos remover cualquier cosa que pudiera identificarle de la información y muestras. Si lo hacemos, esa información y muestras pueden entonces ser usadas para estudios futuros de investigación o dadas a otros investigadores sin obtener permiso adicional. También es posible que en el futuro queramos usar o compartir la información del estudio que pudiera identificarle. Si lo hacemos, una junta de revisión decidirá si necesitamos o no obtener su permiso adicional. Es posible que la información será utilizada en investigaciones futuras de la salud pública. Sin embargo, estas NO incluirán análisis de genético humano.

Al firmar este formulario de consentimiento, está dando permiso para que estos investigadores usen su muestra para un uso futuro.

Ganancia Comercial

Su información y/o muestras pueden ser usadas para producir nuevos productos, pruebas o hallazgos. Esto puede tener valor y puede ser desarrollado y propiedad del equipo de investigación y/u otros. Si esto ocurriera, no hay planes para pagarle.

OTRA INFORMACIÓN

Puede negarse a participar y tiene la libertad de abandonar este estudio en cualquier momento sin penalidad o pérdida de beneficios a los cuales de otra manera tiene derecho (como es a su cuidado médico normal.) Por favor, tenga en cuenta que tiene la opción de aceptar o negar esta prueba del nuevo coronavirus.

Al firmar este formulario, no renuncia a ninguno de sus derechos.

Si participa en este estudio, usted recibirá hasta $10 en tarjeta de regalo por cada hisopo nasal que recolectemos. Recibirá la tarjeta de regalo el mismo día que recolectemos la muestra.

LESIONES RELACIONADAS CON INVESTIGACIÓN

Si cree que ha resultado lesionado por participar en este estudio, contacte a Izzy Brandstetter al (206) 221-4588. La Universidad de Washington normalmente no proporciona compensación por daños excepto a través de su programa discrecional para lesiones industrial. Sin embargo, la ley puede permitir que busque otra compensación si el daño es culpa de los investigadores. Usted no renuncia a ningún derecho a solicitar el pago firmando este formulario de consentimiento.

Declaración del Paciente

Se me ha explicado este estudio. Voluntariamente participo en esta investigación. He tenido la oportunidad de hacer preguntas. Si más tarde tengo preguntas acerca de la investigación, o si he sido lesionado por la participación en este estudio, puedo contactar a uno de los investigadores que figuran en la primera página de este formulario de consentimiento. Si tengo preguntas acerca de mis derechos como participante en la investigación, puedo llamar a la División de Sujetos Humanos al (206) 543-0098 o llamar por cobrar al (206) 221-5940. Recibiré una copia de este formulario de consentimiento.

Participant's First Name: ______________________________

Participant's Last Name: ______________________________

Nombre del participante ______________________________

First name of [participant_first_name]'s parent/legally authorized representative: ______________________________

Last name of [participant_first_name]'s parent/legally authorized representative: ______________________________

Nombre del padre o tutor legal de [part_name_sp] ______________________________

If applicable:
First name of [participant_first_name]'s legally-authorized representative: ______________________________

If applicable:
First name of [participant_first_name]'s legally-authorized representative: ______________________________

Nombre del representante ______________________________

Signature of [participant_first_name]'s legally-authorized representative:

____

Signature of [participant_first_name]'s parent/legally authorized representative:

____

Firma de [parent_name_sp]

____

Firma del representante

____

UNIVERSITY OF WASHINGTON ASSENT TO RESEARCH: Ages 7-12 SEATTLE FLU STUDY Researchers: Helen Y. Chu, MD, MPH, Assistant Professor of Medicine (University of Washington)

Janet A. Englund, MD, Professor of Pediatric Infectious Diseases (Seattle Children's Hospital)

Michael Boeckh, MD, PhD, Professor of Medicine (Fred Hutchinson Cancer Research Center)

Contact: Izzy Brandstetter, MPH, Research Coordinator (University of Washington) (206) 221-4588

Researcher's statement:

We are asking you to be in a research study because you have the symptoms of a respiratory virus (cold) such as a fever, running nose, and cough. We are trying to learn more about the viruses (germs) that cause these symptoms.

Seattle Flu Study researchers, Washington State Department of Health, and the Centers for Disease Control and Prevention (CDC) are closely monitoring an outbreak of respiratory disease. This outbreak is caused by a novel (new) coronavirus. Some person-to-person spread of this virus outside China has been detected. Because of your symptoms today, we would like to test to see if you (or your child) have an infection caused by this new coronavirus.

If you choose to be in the research, we would ask you to do the following:

- Questions: A person on the research team may ask you and/or your parents some questions. You or your parents do not have to answer any sensitive question you don't want to.

- Nasal swab: A little bit of snot would be collected from your nose by putting a swab (Q-tip) in your nose and twirling it around.

- To test for novel coronavirus, the swab may be sent to a public health laboratory. If the test is positive, you may be contacted by your local health department. You might be asked to restrict your movement. This may mean that you (your child) would need to stay away from other people until the infection has cleared. Also, you might be asked about who you have been in contact with since becoming ill. The people you (your child) have been in contact with might be contacted to see if they are ill too. You may also be asked to provide additional swabs for testing. You may not be contacted if the first test result is negative.

- Tissue: We might take a bit of snot from your nose by asking you to blow your nose in a facial tissue like Kleenex.

The nose swab test might hurt just a little bit. It might make you feel like you have to sneeze or cough. Some of the questions might make you uncomfortable. You do not have to answer any question you don't want to.

This research will not help you. We do hope to learn something from this research though. Results from the novel coronavirus test could help identify spread of the virus in the community.

Please talk this over with your parents before you decide whether or not to do this. We will also ask your parents if it is okay for you to be in this study. But even if your parents say "yes" you can still decide not to do this.

If you don't want to be in the study, you don't have to participate. Remember, being in this study is up to you and no one will be upset if you don't want to participate or even if you change your mind later and want to stop.

You can ask any questions about this study. If you have questions later you can call a researcher at (206) 221-4588.

Signing your name at the bottom means that you agree to be in this study. You and your parents will be given a copy of this form after you have signed it.

Your statement:

This research has been explained to me. I agree to take part in this study. I have had a chance to ask questions. If I have more questions, I can ask the doctor or researcher.

Participant's Signature:

____

Firma del participante

____

Don't forget to hit SAVE on your signature!

!No lo olvide presionar guardar en su firma!

HIPAA Authorization Permission to Use, Create and Share Health Information for Research Study Title: Seattle Flu Study

PI Name: Janet Englund, MD

PI Contact Information: 206-987-2239

The purpose of this form is to give your permission to the research team to collect, use or share your protected health information (PHI). Please read this form carefully.

To be in this research study, you must sign this permission form. After reading this form, you can refuse to sign this form. If you do n ot want to sign this permission form, this will not affect the care and treatment you receive. You will also be asked to sign a research consent form that describes details about the research. If you have questions about this permission form or the research consent form you can ask the research team at any time.

The word "you/your" in this form may refer to you or your child.

What Will Happen with Your Protected Health Information? If you join the study, we will keep your information confidential as provided by law.

You have certain privacy rights with regards to your health information, and only with your permission may we collect, use, or share your health information for this study. The following describes the type of information the study will create, use or share, who may use it or share it, and the purposes for which it may be used or shared.

This information may include things like:

- Past or future medical records,

- Research records, such as surveys, questionnaires, interviews, or self-reports about medical history

- Medical or laboratory records related to this study, and

- Information specific to you like your name, address, or birthday

This information may be used by or shared with:

- Researchers (such as doctors and their staff) taking part in this study here and at other centers,

- Research sponsors - this includes any persons or companies working for, with, or owned by the sponsor,

- Review boards (such as Seattle Children's Institutional Review Board), data and safety monitoring boards, and others responsible for watching the conduct of research (such as monitors),

- Governmental agencies like the U.S. Food and Drug Administration (FDA) and the Department of Health and Human Services (DHHS), including similar agencies in other countries, and

- Public health authorities to whom we are required by law to report information for the prevention or control of disease, injury, abuse, or disability.

- If the sponsor pays any of your medical expenses, we may be required to give the sponsor your name, date of birth, and Medicare ID or social security number.

This information may be used or shared to:

- Complete and publish the results of the study described in this form,

- Study the results of this research,

- Check if this study was done correctly, and

- Comply with non-research obligations (if we think you or someone else could be harmed).

You may looik at or copy the information that may be used or disclosed. However, for certain types of research studies, some of the research information may not be available to you during the study. This does not affect your right to see what is in your medical (hospital) records.

There is no time limit for the use or sharing of your information. Researchers continue to analyze data for many years, and it is not always possible to know when they will be done. If your information will be banked as part of this study, it may be used in the future for other research. We would not ask for your permission prior to this future research.

Your permission for the use or sharing of your information will not expire, but you may cancel it at any time. You can do this by notifying the study team in writing. If you cancel your permission, no new information will be collected about you, but information that has already been collected may still be used and shared with others.

The use or sharing of your information will follow privacy laws, but these laws only apply to doctors, hospitals, and other health care providers. Some people who receive your health information as part of this study may share it with others without your permission if doing so is permitted by the laws they must follow.

If the results of the study are published, information that identifies you would not be used.

Your permission is documented by signing this form below. If you decide that we cannot use or share your information, you cannot participate in this study.

Permission I have read this form describing how health information will be used. I have had a chance to ask questions aout the use of health information and I have received answers to my questions.

By signing this form, I agree to the creation, use, and sharing of my or my child's health information for the purposes of this research study. I will be given a copy of this signed form.

UNIVERSIDAD DE WASHINGTON
ACUERDO PARA LA INVESTIGACIÓN: EDADES 7-12
ESTUDIO DE LA INFLUENZA EN SEATTLE

Investigadores:
Dra. Helen Y. Chu, Profesora Asistente de Medicina (Universidad de Washington)
Dra. Janet. A Englund, Profesora de Enfermedades Infecciosas Pediátricas (Hospital Seattle Children)
Dr. Michael Boeckh, Profesor de Medicina (Centro de Investigación para el Cáncer Fred Hutchinson)

Contacto:
Izzy Brandstetter, MPH, Coordinadora de investigación (Universidad de Washington) (206) 221-4588

Declaración del Investigador:
Le pedimos que participe en estudio de investigación porque tiene los síntomas de la gripe. Estamos tratando de aprender más acerca de los gérmenes que causan estos síntomas.

Investigadores del Estudio de la Influenza en Seattle, el Department de Salud del Estado de Washington, y los Centros de Control y Prevención de Enfermedades (CDC) están monitoreando de cerca de un brote de una enfermedad respiratoria. Esta brote se causa por el coronavirus nuevo. Alguna propagación de esta enfermedad entre personas a fuera de China ha sido detectada. Debido a sus síntomas de hoy, nos gustaría ver si usted tiene una infección causada por esta virus nuevo. Esta información no estará en el internet para que todos la vean.

Si elige participar en la investigación, le pediremos hacer lo siguiente:

● Preguntas: Una persona del equipo de investigación le puede hacer a usted y/o sus padres algunas preguntas. Usted o sus padres no tienen que contestar ninguna pregunta delicada sino quieren.
● Muestra nasal: Se recogería un poco de moco de su nariz por colocando un hisopo (punta Q) a dentro su nariz y girándolo.

● Para probar para el nuevo coronavirus, el hisopo puede ser enviado a un laboratorio de salud pública. Si la prueba es positiva, puede estar contactado por el departamento local de la salud pública. Se le puede pedir que restrinja su movimiento. Esto puede significar que usted necesitaría mantenerse alejado de ostras personas hasta que la infección haya desaparecido. Además, es posible que le pregunten sobre con quien ha estado en contacto desde que se enfermó. Las personas con las que usted ha estado en contacto pueden ser contactados para ver si también están enfermos. También se le puede pedir que proporcione hisopos adicionales para probar Es posible que no le contacemos si el primer resultado de la prueba es negativo.
● Pañuelo de papel: Podríamos quitarle un poco de moco de la nariz al pedirle que se suene la nariz con un pañuelo de papel facial como Kleenex.

Esas muestras nasales pueden doler solo un poco. Puede hacerle sentir como que tiene que estornudar o toser. Algunas de las preguntas pueden incomodarle. No tiene que contestar ninguna pregunta que no desee.

Esta investigación no le ayudará. Sin embargo esperamos aprender algo de esta investigación. Resultados del coronavirus nuevo pudieran ayudar a identifar la propagación del virus en la comunidad.

Favor de hablar de esto con sus padres antes de decidir si hace o no esto. Les preguntaremos a sus padres si está bien que usted participe en este estudio. Pero aunque sus padres digan "Sí" usted aún puede decidir no hacerlo.

Si no quiere participar en el estudio, no tiene que participar. Recuerde, participar en el estudio depende de usted y nadie estará molesto si no quiere participar o si incluso cambia de opinión más tarde y quiere no participar más. Puede hacer cualquier pregunta acerca del estudio. Si tiene preguntas más adelante puede llamar al investigador al (206) 221-4588.

Firmar su nombre en la parte inferior significa que está de acuerdo en participar en el estudio. A usted y sus padres se les dará una copia de este formulario después de haberlo firmado.

Su declaración:
Se me ha explicado esta investigación. Acepto participar en este estudio. He tenido la oportunidad de hacer preguntas. Si tengo más preguntas, puedo hacerlas al médico o investigador.

Permiso para usar, crear y compartir la información de salud para investigación

Nombre del estudio: Estudio de la Influencia en Seattle
Nombre del IP: Janet Englund, MD
Información del contacto del IP: 206-987-2239

El propósito de este formulario es dar su permiso al equipo de investigación para recopilar, usar o compartir su información de salud protegida (PHI siglas en inglés). Por favor lea este formulario cuidadosamente.

Para participar en este estudio de investigación, debe de firmar este formulario de consentimiento. Después de leer este formulario, puede negarse a firmar este formulario. Si no desea firmar este formulario de autorización, esto no afectará el cuidado y tratamiento que recibe. También se le pedirá firmar el consentimiento del formulario de investigación que describe los detalles acerca de la investigación. Si tiene preguntas acerca de este formulario de permiso o el formulario de consentimiento de investigación puede consultar al equipo de investigación en cualquier momento.

La palabra “usted/su” en este formulario puede referirse a usted o su hijo.
¿Qué pasará con su información de salud protegida?
Si participa en el estudio, mantendremos la confidencialidad de su información según lo dispuesto por la ley.

Usted tiene ciertos derechos de privacidad con respeto a su información de su salud, y solo con su permiso podemos recopilar, usar, o compartir su información de salud para este estudio. A continuación se describe el tipo de información que el estudio creará, usará y compartirá, quién pudiera usarla o compartirla, y los propósitos por los cuales pudiera ser usada o compartida.

Esta información puede incluir cosas como:
• Expedientes médicos pasados o futuros,
• Registros de investigación, como encuestas, cuestionarios, entrevistas, o auto informes acerca de antecedentes médicos.
• Expedientes médicos o de laboratorio relacionados a este estudio, y
• Información específica de usted como su nombre, domicilio, o fecha de nacimiento.

Esta información puede ser usada por o compartida con:
• Investigadores (como médicos y su personal) que participan en este estudio aquí o en otros centros,
• Patrocinadores de la investigación- esto incluye cualquier persona o compañías trabajando por, con, o que pertenezcan al patrocinador.
• Las juntas de revisión (como sería la junta de Revisión Institucional del hospital Children’s de Seattle), las juntas de monitoreo de datos y seguridad, y otras personas responsables de vigilar la conducta de los investigadores (como los observadores),
• Agencias gubernamentales como la Administración de Alimentos y Medicamentos (FDA siglas en inglés) y el Departamento de Salud y Servicios Humanos y (DHHS siglas en inglés) incluyendo agencias similares en otros países, y
• Las autoridades de salud pública a quienes estamos obligados por ley a reportar la información para la prevención o control de enfermedades, lesión, abuso o discapacidad.
• Si el patrocinador paga cualquiera de sus gastos médicos, podemos ser obligados darle al patrocinador su nombre, fecha de nacimiento, número de identificación de Medicare o número de seguro social.

Esta información puede ser usada o compartida para:
• Completar y publicar los resultados del estudio descrito en este formulario,
• Estudiar los resultados de esta investigación,
• Revisar si este estudio fue realizado correctamente, y
• Cumplir con las obligaciones que no son parte de la investigación (si pensamos que usted o alguien más pudiera resultar perjudicado).

Puede ver o copiar la información que puede ser usada o divulgada. Sin embargo, para ciertos tipos de estudios de investigación, algo de la información de investigación no le esté disponible durante el estudio. Esto no afecta su derecho de ver lo que se encuentra en su expediente médico (del hospital)

No hay límite de tiempo para el uso o intercambio de su información. Los investigadores continúan analizando su información durante muchos años, y no siempre es posible saber cuándo terminarán. Si su información se guarda en el banco como parte de este estudio, pudiera ser usada en el futuro para otra investigación. No le pediremos su permiso antes de esta investigación futura.

Su permiso para el uso o el intercambio de su información no caducará, pero puede cancelarla en cualquier momento. Puede hacerlo notificando por escrito al equipo de investigación. Si cancela su permiso, no se recopilará nueva información suya, pero la información de usted que ya ha sido recopilada puede todavía ser usada y compartida con otros.

El uso o el intercambio de su información cumplirá con las leyes de privacidad, pero estas leyes solo se emplean con los médicos, hospitales, y otros proveedores del cuidado de la salud. Algunas personas que reciben su información de salud como parte de este estudio pueden compartirla con otros sin su autorización, si hacerlo es permitido por las leyes que ellos deben cumplir.

Si el resultado del estudio es publicado, la información que le identifica no será usada.

Su permiso es documentado al firmar este formulario a continuación. Si usted decide que no podemos usar o compartir su información, no puede participar en el estudio.

Permiso

He leído este formulario describiendo como la información de la salud será usada. He tenido la oportunidad de hacer preguntas acerca del uso de la información de salud y he recibido las respuestas a mis preguntas.

Al firmar este formulario, acepto la creación, uso y el intercambio de la información de salud mía o de mi hijo para los fines de este estudio de investigación. Se me proporcionará una copia firmada de este formulario.

[participant_first_name] [participant_last_name]'s signature: ______________________

Firma del participante ______________________

Firma de [parent_name_sp] ______________________

[parent_first_name] [parent_last_name]'s signature: ______________________

Seattle Children's Researcher Full Name: ______________________

Seattle Children's Researcher's Signature: ______________________

!No lo olvide presionar guardar en su firma!

Don't forget to hit SAVE on your signature!

Consent Date ______________________

We would like to email you. If you choose to enroll, we will use this email address to:

- Send you a copy of your consent form

- Ask you follow-up questions about your illness

- Send you your sample barcode so you can view your test results online

- Send you more information about this study and related topics

You may opt out of receiving emails at any time. We will not share your email with anyone outside of the study team.

(Ex: myemail@email.com)

Quisiéramos enviarte correos. Si eliges inscribir, usaremos este emai para:

Enviarle una copia de su formulario de consentimiento Hacerle preguntas de seguimiento sobre su enfermedad Enviarle su código de barras de muestra para que pueda ver los resultados de su prueba en línea Enviarle más información sobre este estudio y temas relacionados Puede optar por no recibir mensajes de correo electrónico en cualquier momento. No divulgaremos su correo electrónico a nadie fuera del equipo de estudio.

(Ex: myemail@email.com)

PDF of Signed Consent Form

PDF of Signed Unable to Assent Notes To File

Necessary consent forms uploaded for participant?
(Saying yes triggers automated email to participant).

○ Yes
○ No
