# Supplementary material for: “I probably shouldn’t go in today”: Inequitable access to paid sick leave and its impacts on health behaviors during the emergence of COVID-19 in the Seattle area
Source: PLoS One. 2024 Sep 10;19(9):e0307734. doi: 10.1371/journal.pone.0307734 (PMC11386467; doi:10.1371/journal.pone.0307734)
Supplement: S2 File — (ZIP) [file pone.0307734.s002.zip › Copy of AppendixA2_SFS_Swab_Send_Consent.pdf]

Consent

Please re- enter your email address for confirmation.

____

Please enter the participant's date of birth. This will help us determine which consent form(s) should be signed.

____
(MM-DD-YYYY)

Age of enrollee (years)

____

Age of enrollee (months)

____

UNIVERSITY OF WASHINGTON

CONSENT FORM: Ages 18 and up

ASSENT FORM: Ages 13-17

SEATTLE FLU STUDY: SWAB & SEND

Researchers:

Helen Y. Chu, MD, MPH, Assistant Professor of Medicine (University of Washington)

Janet. A Englund, MD, Professor of Pediatric Infectious Diseases (Seattle Children's Hospital)

Michael Boeckh, MD, PhD, Professor of Medicine (Fred Hutchinson Cancer Research Center)

Contact:

Izzy Brandstetter, MPH, Research Coordinator (University of Washington) (206) 221-4588

We are asking you to be in a research study. This form gives you information to help you decide whether or not to be in the study. Being in the study is voluntary. Please read this carefully. You may ask any questions about the study. Then you can decide whether or not you want to be in the study.

This form also serves as an assent form for individuals age 13-17. This means that if you choose to take part in this research study, you would sign this form to confirm your choice. Your parent or legally-authorized representative would also need to give their permission and sign this form for you to join the study.

This form is also used for parents and legally-authorized representatives of subjects to provide permission to the research team for individuals who are not capable of providing permission themselves. In such cases, the term "you" refers to the subject or your child.

PURPOSE OF THE STUDY

Colds are common in children and adults, and can cause a runny nose, cough, or trouble breathing. We want to understand how people get sick with these cold germs and how they spread from person to person. Once we understand how the germs start and spread, we can have a better plan to prevent them from making people sick. We also want to provide people with a way to see what germs are in their neighborhood. This information will be on the internet for everyone to see.

STUDY PROCEDURES

We are testing children and adults who might have a cold. We are inviting you to participate because you have the symptoms of a cold. This study involves:

· Answering questions about your health, who you are around, and your living situation. We think these questions will take about 15 minutes. You may choose not to answer any sensitive question that you do not want to.

· Doing a swab of your (or your child's) nose to look for germs, and mailing it back to our office. We will provide the swabbing kit and the postage to mail it back.

· Emailing you to ask questions about your cold in about 1 week.

This study does not replace care you would receive from your doctor, and your participation is voluntary.

RISKS, STRESS, OR DISCOMFORT

We do not expect serious side effects from this study. Getting the nasal swab may cause mild discomfort, watery eyes, or sneezing. Some of the questions that we might ask you are sensitive and may make you feel uncomfortable. You do not have to answer any sensitive question that you do not want to. There is a risk that your privacy could be breached. We will do everything that we can to make sure that this does not happen.

BENEFITS OF THE STUDY

This study will not benefit you directly. It may help others in the future by learning what germs make people sick. We hope that the neighborhood map helps you understand what germs are in your community.

SOURCE OF FUNDING

The University of Washington is receiving financial support from an anonymous donor.

CONFIDENTIALITY OF RESEARCH INFORMATION

Your record will be kept secret. You will not be identified in any report about this study. Your study record will never be used against you.

The information we collect from you as well as the germs that make you sick will be on a public website to show the spread of germs in your community. This information will not be shared in a way that will reveal your identity.

Government and university staff sometimes review studies such as this one to make sure they are being done safely and legally. If a review of this study takes place, your records may be examined. The reviewers will protect your privacy. The study records will never be used against you.

Washington State law requires that we report certain conditions to local public health jurisdictions or the Department of Health. If your sample tests positive for one of these conditions, a local health jurisdiction or the Department of Health might contact you with more questions. They will keep your information secret.

USE OF INFORMATION AND SPECIMENS

Characterizing Germs

We will characterize the germs that are making you sick and share information about them. Information that identifies you would not be shared on the internet.

Returning Results to You

You may receive a link to a website where you can view the results of your nose swab test within three months of when the swab is collected. You can enter your nose swab barcode to view your results when they are ready. The results may tell you what germs are making you sick, how they are characterized, and advice on how to keep other people from getting sick. You are not required to go to the website and look at your results if you do not want to. If you do not have access to the internet and would like to know your results, you may bring your barcode to one of our community kiosks, or call 206-221-4588. We may not be able to give you your results if you lose your barcode.

Using Your Data in Future Research

Storing samples so researchers can use them in the future is called "banking." Researchers also bank information and samples so they can share it with other researchers.

The information and/or samples that we obtain from you for this study might be used for future studies. We may remove anything that might identify you from the information and samples. If we do so, that information and samples may then be used for future research studies or given to another researcher without getting additional permission from you. It is also possible that in the future we may want to use or share study information that might identify you. If we do, a review board will decide whether or not we need to get additional permission from you.

By signing this consent form, you are giving your permission for these researchers to use your sample for future use.

Commercial Profit

Your data and/or samples may be used to make new products, test, or findings. These may have value and may be developed and owned by the research team and/or others. If this happens, there are no plans to pay you.

OTHER INFORMATION

You may refuse to participate and you are free to leave this study at any time without penalty or loss of benefits to which you are otherwise entitled (such as your normal medical care). By signing this form, you do not give up any of your rights.

If you join this study, you will receive gift card of up to $10 for each nose swab that we collect. You will receive the gift card within a month of us receiving your sample.

RESEARCH-RELATED INJURY

If you think you have been harmed from being in this research, contact Izzy Brandstetter at (206) 221-4588. The University of Washington does not normally provide compensation for harm except through its discretionary program for medical industry. However, the law may allow you to seek other compensation if the harm is the fault of the researchers. You do not waive any right to seek payment by signing this consent form.

Subject's statement

This study has been explained to me. I volunteer to take part in this research. I have had a chance to ask questions. If I have questions later about the research, or if I have been harmed by participating in this study, I can contact one of the researchers listed on the first page of this consent form. If I have questions about my rights as a research subject, I can call the Human Subjects Division at (206) 543-0098 or call collect at (206) 221-5940. I will receive a copy of this consent form.

Note: The term "participant" refers to the minor/child who is currently feeling sick.	
Participant's First Name:	______________________
Participant's Last Name:	______________________
Participant's Signature:	____________________________
First name of [first_name_1]'s parent/legally authorized representative:	______________________
Last name of [first_name_1]'s parent/legally authorized representative:	______________________
Signature of [first_name_1]'s parent/legally authorized representative:	____________________________

UNIVERSITY OF WASHINGTON

ASSENT TO RESEARCH: Ages 7-12

SEATTLE FLU STUDY: SWAB & SEND

Researchers:

Helen Y. Chu, MD, MPH, Assistant Professor of Medicine (University of Washington)

Janet. A Englund, MD, Professor of Pediatric Infectious Diseases (Seattle Children's Hospital)

Michael Boeckh, MD, PhD, Professor of Medicine (Fred Hutchinson Cancer Research Center)

Contact:

Izzy Brandstetter, MPH, Research Coordinator (University of Washington) (206) 221-4588

Researcher's statement:

We are asking you to be in a research study because you have the symptoms of cold We are trying to learn more about the germs that cause these symptoms.

If you choose to be in the research, we would ask you to do the following:

● Questions: You and/or parents would answer some questions about your health on an electronic device. You do not have to answer any sensitive questions that you do not want to.

● Nasal swab: Your parents would take a little bit of snot from your nose by putting a swab (Q-tip) in your nose and twirling it around.

The nose swab test might hurt just a little bit. It might make you feel like you have to sneeze or cough. Some of the questions might make you uncomfortable. You do not have to answer any sensitive question you don't want to.

This research will not help you. We do hope to learn something from this research though.

Please talk this over with your parents before you decide whether or not to do this. We will also ask your parents if it is okay for you to be in this study. But even if your parents say "yes" you can still decide not to do this.

If you don't want to be in the study, you don't have to participate. Remember, being in this study is up to you and no one will be upset if you don't want to participate or even if you change your mind later and want to stop.

You can ask any questions about the study. If you have a question later you can call a researcher at (206) 221-4588.

Signing your name at the bottom means that you agree to be in this study. You and your parents will be given a copy of this form after you have signed it.

Your statement:

This research has been explained to me. I agree to take part in this study. I have had a chance to ask questions. If I have more questions, I can ask the doctor or researcher.

[first_name_1] [last_name_1]'s Signature: __________________________________

Consent Date __________________________

If you have any questions about the consent form, please feel free to contact our study team at Seattleflu@uw.edu or (206) 221-4588 during normal business hours.

Please continue to the next page to confirm your information.
