# Supplementary material for: “I probably shouldn’t go in today”: Inequitable access to paid sick leave and its impacts on health behaviors during the emergence of COVID-19 in the Seattle area
Source: PLoS One. 2024 Sep 10;19(9):e0307734. doi: 10.1371/journal.pone.0307734 (PMC11386467; doi:10.1371/journal.pone.0307734)
Supplement: S2 File — (ZIP) [file pone.0307734.s002.zip › Copy of AppendixB_SFS_Enrollment_Questionnaire.pdf]

Enrollment Questionnaire

If you would like to receive a paper copy of the consent form that you just signed, please ask a Seattle Flu Study staff member now.	◯ Okay
Si desea recibir una copia impresa del formulario de consentimiento que acaba de firmar, dígaselo ahora a un miembro del personal del Estudio de la Gripe de Seattle.	◯ Okay
What day did your current symptoms start?<br><br>Note: Today's date is highlighted in yellow on the calendar.	________________________<br>(MM-DD-YYYY)
¿En qué día comenzaron los síntomas que tiene ahora?<br><br>Nota: La fecha de hoy está resaltada en amarillo en el calendario.	________________________<br>(MM-DD-YYYY)
Days sick calculation	________________________
Around when did your symptoms start?	◯ Half a day ago<br>◯ Half a day - 1 day ago<br>◯ 1 - 1.5 days ago<br>◯ 1.5 - 2 days ago<br>◯ 3 days ago<br>◯ 4 days ago<br>◯ 5 or more days ago
¿Más o menos, ¿cuándo comenzaron sus síntomas?	◯ Hace medio día<br>◯ Hace medio día a 1 día<br>◯ Hace 1 a 1.5 días<br>◯ Hace 1.5 a 2 días<br>◯ Hace 3 días<br>◯ Hace 4 días<br>◯ Hace 5 o más días
How long did it take you to go from feeling not sick at all to feeling the sickest you have felt?	◯ Half a day<br>◯ Half a day - 1 day<br>◯ 1 - 1.5 days<br>◯ 1.5 - 2 days<br>◯ 3 days<br>◯ 4 days<br>◯ 5 or more days
¿En cuánto tiempo pasó de no sentirse mal a sentirse lo más enfermo que se ha sentido?	◯ Medio día<br>◯ Medio día a 1 día<br>◯ 1 a 1.5 días<br>◯ 1.5 a 2 días<br>◯ 3 días<br>◯ 4 días<br>◯ 5 o más días

How severe are your symptoms? Select the level of discomfort you felt at the worst point so far).

¿Qué tan graves son sus síntomas? (Seleccione el nivel de malestar que tuvo en el peor momento hasta ahora).

	Mild	Moderate	Severe
Feeling feverish	◯	◯	◯
Cough	◯	◯	◯
Muscle or body aches	◯	◯	◯
Feeling more tired than usual	◯	◯	◯
Sore throat or itchy/scratchy throat	◯	◯	◯

	Leve	Moderado	Intenso
Sentirse febril	◯	◯	◯
Tos	◯	◯	◯
Dolor muscular o corporal	◯	◯	◯
Sentirse más cansado/a de lo normal	◯	◯	◯
Dolor o picazón de garganta	◯	◯	◯

You are eligible for a rapid flu test. It will take 20-30 minutes for you to get a test result (by paper or via email) after you complete the questionnaire. Are you interested in having your sample tested for the flu and RSV for free?

◯ Yes ◯ No

You did not enter your email address. If you would like to receive your results by email when they are ready, please enter your email address here.

____

Usted reúne los requisitos para una prueba rápida de la gripe. Recibirá el resultado de la prueba en 20 a 30 minutos después de completar el cuestionario. ¿Le interesa que analicen su muestra gratis para ver si tiene la gripe?

◯ Sí ◯ No

No ingresó su dirección de correo electrónico. Si desea recibir sus resultados por correo electrónico cuando estén listos, ingrese su dirección de correo electrónico.

____

Demographics

Demografía

What is the sex on your medical records?

◯ Male
◯ Female
◯ Indeterminate/other
◯ Prefer not to say

What is your sex?

◯ Male
◯ Female
◯ Other (please specify)
◯ Prefer not to say

Other sex:	__________________________________
¿Cuál es su sexo indicado en sus expedientes médicos?	◯ Masculino<br>◯ Femenino<br>◯ Indeterminado/otro<br>◯ Prefiero no decir
¿Cuál es su sexo?	◯ Masculino<br>◯ Femenino<br>◯ Otra (por favor explique)<br>◯ Prefiero no decir
Otro sexo:	__________________________________
Are you Hispanic or Latino?	◯ Yes<br>◯ No<br>◯ Prefer not to say
¿Es de origen hispano o latinoamericano?	◯ Sí<br>◯ No<br>◯ Prefiero no decir
How would you describe your race? Select all that apply.	☐ American Indian or Alaska Native<br>☐ Asian<br>☐ Native Hawaiian or other Pacific Islander<br>☐ Black or African American<br>☐ White<br>☐ Other<br>☐ Prefer not to say
¿Cómo describiría su raza? Seleccione todas las que correspondan.	☐ Indio americano o nativo de Alaska<br>☐ Asiático<br>☐ Nativo de Hawái o de otra isla del Pacífico<br>☐ Negro o afroamericano<br>☐ Blanco<br>☐ Otra<br>☐ Prefiero no decir
What is the highest level of education you have completed?	◯ Less than high school graduate<br>◯ Graduated high school/obtained GED<br>◯ Some college (including vocational training, associate's degree)<br>◯ Bachelor's degree<br>◯ Advanced degree<br>◯ Prefer not to say
¿Cuál es el nivel más alto de educación que ha completado?	◯ No acabé la preparatoria (high school)<br>◯ Terminé la preparatoria (high school) / Obtuve mi GED<br>◯ Algunos estudios universitarios (incluida la formación vocacional, título de dos años)<br>◯ Licenciatura<br>◯ Título avanzado<br>◯ Prefiero no decir

Please choose the range that best represents your household income last year (before taxes). If you are still considered a "dependent" for tax purposes, choose the range that describes your parent/legal guardian's household income.	◯ Less than or equal to $25,000<br>◯ Between $25 and 50 thousand ($25,001 to $50,000)<br>◯ Between $50 and 75 thousand ($50,001 to $75,000)<br>◯ Between $75 and 100 thousand ($75,001 to $100,000)<br>◯ Between $100 and 125 thousand ($100,001 to $125,000)<br>◯ Between $125 and 150 thousand ($125,001 to $150,000)<br>◯ Over $150,000<br>◯ Don't know<br>◯ Prefer not to say
Por favor, elija el intervalo que es la mejora representación de su ingreso de hogar en el año pasado (antes de los impuestos). Si todavía está considerado/a como un/a dependiente por los impuestos, elija el intervalo que describe el ingreso de hogar de su padre / tutor legal.	◯ Menos que o igual a $25,000<br>◯ Entre veinticinco y cincuenta mil dolares ($25,001 a $50,000)<br>◯ Entre cincuenta y setenta y cinco mil dolares ($50,001 a $75,000)<br>◯ Entre setenta y cinco y cien mil dolares ($75,001 a $100,000)<br>◯ Entre cien mil y cien y veinticinco mil dolares ($100,001 a $125,000)<br>◯ Entre cien y veinticinco mil y cien y cincuenta mil dolares ($125,001 a $150,000)<br>◯ Más que $150,000<br>◯ No lo sé<br>◯ Prefiero no decir
What type of health insurance do you have? Select all that apply.	☐ Private (provided by employer and/or purchased)<br>☐ Government (Medicare/Medicaid)<br>☐ Other<br>☐ None<br>☐ Prefer not to say
¿Qué tipo de seguro médico tiene? Seleccione todas las que correspondan.	☐ Privado (proporcionado por el empleador y/o comprado)<br>☐ Gubernamental (Medicare/Medicaid)<br>☐ Otra<br>☐ Ninguno<br>☐ Prefiero no decir
Are you currently pregnant?	◯ Yes<br>◯ No<br>◯ Prefer not to say
¿Está embarazada?	◯ Sí<br>◯ No<br>◯ Prefiero no decir

Next, we will ask you some questions about where you live, including your home address.

Remember: Your address will only be used to convert your location to a census tract. It will not be given to anyone outside the study team.

A continuación, le haremos algunas preguntas sobre dónde vive, incluso le pediremos su dirección residencial. Su dirección solo se utilizará para convertirla en una división censal. No se le dará a nadie fuera del equipo de estudio.

Question	Options
Are you affiliated with the University of Washington?	◯ Yes, I am an undergraduate student<br>◯ Yes, I am a graduate/professional student<br>◯ Yes, I am a faculty member<br>◯ Yes, I am a staff member/university employee<br>◯ No
¿Tiene alguna afiliación a la Universidad de Washington?	◯ Sí, Soy estudiante de pregrado<br>◯ Sí, Soy estudiante de posgrado/estudios profesionales<br>◯ Sí, Soy miembro del profesorado<br>◯ Sí, Soy miembro del personal/empleado universitario<br>◯ No
What kind of residence do you live in?	◯ On-campus residence hall<br>◯ On-campus apartment<br>◯ Greek life housing<br>◯ Off-campus house<br>◯ Off-campus apartment<br>◯ Other
¿En qué tipo de residencia vive?	◯ Dormitorio en el campus<br>◯ Apartamento en el campus<br>◯ Casa de hermandad<br>◯ Casa fuera del campus<br>◯ Apartamento fuera del campus<br>◯ Otra
Where do you live?	◯ House/condo/townhouse<br>◯ Shelter<br>◯ Apartment<br>◯ Dormitory<br>◯ Assisted living facility<br>◯ Skilled nursing center<br>◯ No consistent primary residence<br>◯ Other
¿Dónde vive?	◯ Casa/condominio/casa adosada<br>◯ Refugio<br>◯ Apartamento<br>◯ Dormitorio<br>◯ Centro de vivienda asistida<br>◯ Centro de enfermería especializada<br>◯ Sin residencia principal regular<br>◯ Otra

Which dorm do you live in?	◯ Alder Hall<br>◯ Cedar Apartments<br>◯ Elm Hall<br>◯ Haggett Hall<br>◯ Hansee Hall<br>◯ Lander Hall<br>◯ Madrona Hall<br>◯ Maple Hall<br>◯ McCarty Hall<br>◯ McMahon Hall<br>◯ Mercer Court Apartments<br>◯ Poplar Hall<br>◯ Stevens Court Apartments<br>◯ Terry Hall<br>◯ Willow Hall<br>◯ Other
¿En qué dormitorio vive?	◯ Alder Hall<br>◯ Cedar Apartments<br>◯ Elm Hall<br>◯ Haggett Hall<br>◯ Hansee Hall<br>◯ Lander Hall<br>◯ Madrona Hall<br>◯ Maple Hall<br>◯ McCarty Hall<br>◯ McMahon Hall<br>◯ Mercer Court Apartments<br>◯ Poplar Hall<br>◯ Stevens Court Apartments<br>◯ Terry Hall<br>◯ Willow Hall<br>◯ Other
What is your usual nighttime accommodation?	◯ Shelter<br>◯ Transitional housing/safe haven<br>◯ Street/outside/tent/encampment<br>◯ Abandoned building/squat<br>◯ Vehicle (car, van, RV, camper)<br>◯ Hotel or motel<br>◯ Prefer not to say<br>◯ None of the above
¿Cuál es su alojamiento nocturno habitual?	◯ Refugio<br>◯ Vivienda de transición/refugio seguro<br>◯ Calle/afuera/tienda de campaña/campamento<br>◯ Edificio abandonado/donde pueda<br>◯ Vehículo (automóvil, camioneta, vehículo recreativo, autocaravana)<br>◯ Hotel o motel<br>◯ Prefiero no decir<br>◯ Ninguna de las anteriores respuestas

Which shelter has served as your primary residence over the last 7 days?	◯ Aloha Inn<br>◯ Blaine Center Homeless Ministry<br>◯ Bread of Life Mission<br>◯ Compass Housing Alliance<br>◯ DESC (Downtown Emergency Service Center)<br>◯ Elizabeth Gregory House<br>◯ Hammond House Women's Shelter<br>◯ Jubilee Women's Center<br>◯ King County Men's Winter Shelter<br>◯ Mary's Place<br>◯ Noel House Women's Referral Center<br>◯ Pike Market Senior Center<br>◯ Roots Young Adult Shelter<br>◯ Sacred Heart Shelter<br>◯ Saint Martin de Porres Shelter<br>◯ Salvation Army Women's Shelter<br>◯ Seattle City Hall Shelter<br>◯ Seattle Union Gospel Mission for Men<br>◯ YMCA Emergency Shelter<br>◯ Other/none of the above
¿Cuál refugio ha sido su residencia principal en los últimos 7 días?	◯ Aloha Inn<br>◯ Blaine Center Homeless Ministry<br>◯ Bread of Life Mission<br>◯ Compass Housing Alliance<br>◯ DESC (Downtown Emergency Service Center)<br>◯ Elizabeth Gregory House<br>◯ Hammond House Women's Shelter<br>◯ Jubilee Women's Center<br>◯ King County Men's Winter Shelter<br>◯ Mary's Place<br>◯ Noel House Women's Referral Center<br>◯ Pike Market Senior Center<br>◯ Roots Young Adult Shelter<br>◯ Sacred Heart Shelter<br>◯ Saint Martin de Porres Shelter<br>◯ Salvation Army Women's Shelter<br>◯ Seattle City Hall Shelter<br>◯ Seattle Union Gospel Mission for Men<br>◯ YMCA Emergency Shelter<br>◯ Otro/ninguno de los anteriores
Home address: Please enter your house number and street. (You will be asked for your city, state, and zip code in the next questions).	__________________________<br>(Ex: 1234 NE 56th St.)
Dirección residencial: Escriba su número de casa y calle. (Se le pedirá su ciudad, estado y código postal en las siguientes preguntas).	__________________________<br>(Ex: 1234 NE 56th St.)
Home address: Please enter your house number and street. (You will be asked for your city, state, and zip code in the next questions).	__________________________<br>(Ex: 1234 NE 56th St.)
Dirección residencial: Escriba su número de casa y calle. (Se le pedirá su ciudad, estado y código postal en las siguientes preguntas).	__________________________<br>(Ex: 1234 NE 56th St.)
Home address: City	◯ Seattle<br>◯ Other (please specify)

Dirección residencial: Ciudad	◯ Seattle<br>◯ Otra (por favor explique)
Home address: City	______________________________
Dirección residencial: Ciudad	______________________________

Home address: State<br><br>Type "None of the above" if you live outside of the US.	◯ Alabama<br>◯ Alaska<br>◯ Arizona<br>◯ Arkansas<br>◯ California<br>◯ Colorado<br>◯ Connecticut<br>◯ Delaware<br>◯ District of Columbia<br>◯ Florida<br>◯ Georgia<br>◯ Hawaii<br>◯ Idaho<br>◯ Illinois<br>◯ Indiana<br>◯ Iowa<br>◯ Kansas<br>◯ Kentucky<br>◯ Louisiana<br>◯ Maine<br>◯ Maryland<br>◯ Massachusetts<br>◯ Michigan<br>◯ Minnesota<br>◯ Mississippi<br>◯ Missouri<br>◯ Montana<br>◯ Nebraska<br>◯ Nevada<br>◯ New Hampshire<br>◯ New Jersey<br>◯ New Mexico<br>◯ New York<br>◯ North Carolina<br>◯ North Dakota<br>◯ Ohio<br>◯ Oklahoma<br>◯ Oregon<br>◯ Pennsylvania<br>◯ Rhode Island<br>◯ South Carolina<br>◯ South Dakota<br>◯ Tennessee<br>◯ Texas<br>◯ Utah<br>◯ Vermont<br>◯ Virginia<br>◯ Washington<br>◯ West Virginia<br>◯ Wisconsin<br>◯ Wyoming<br>◯ American Samoa<br>◯ Guam<br>◯ Northern Mariana Islands<br>◯ Puerto Rico<br>◯ U.S. Minor Outlying Islands<br>◯ U.S. Virgin Islands<br>◯ None of the above<br>(Start typing and state will be suggested for you)

Dirección residencial: Estado

Escriba "Ninguno de los anteriores" si vive fuera de los Estados Unidos.

◯ Alabama
◯ Alaska
◯ Arizona
◯ Arkansas
◯ California
◯ Colorado
◯ Connecticut
◯ Delaware
◯ Distrito de Columbia
◯ Florida
◯ Georgia
◯ Hawaii
◯ Idaho
◯ Illinois
◯ Indiana
◯ Iowa
◯ Kansas
◯ Kentucky
◯ Luisiana
◯ Maine
◯ MD Maryland
◯ Massachusetts
◯ Michigan
◯ Minnesota
◯ Mississippi
◯ Missouri
◯ Montana
◯ Nebraska
◯ Nevada
◯ New Hampshire
◯ New Jersey
◯ New Mexico
◯ New York
◯ Carolina del Norte
◯ Dakota del Norte
◯ Ohio
◯ Oklahoma
◯ Oregon
◯ Pensilvania
◯ Rhode Island
◯ Carolina del Sur
◯ Dakota del Sur
◯ Tennessee
◯ Texas
◯ Utah
◯ Vermont
◯ Virginia
◯ Washington
◯ Virginia Occidental
◯ Wisconsin
◯ Wyoming
◯ Samoa Americana
◯ Guam
◯ Islas Marianas del Norte
◯ Puerto Rico
◯ Islas Ultramarinas Menores de EE. UU.
◯ Islas Vírgenes de EE.UU
◯ Ninguna de las anteriores respuestas
(Comience a escribir y aparecerán sugerencias del estado)

Home address: Country

◯ Afghanistan
◯ Åland Islands
◯ Albania
◯ Algeria
◯ American Samoa
◯ Andorra
◯ Angola
◯ Anguilla
◯ Antarctica
◯ Antigua and Barbuda
◯ Argentina
◯ Armenia
◯ Aruba
◯ Australia
◯ Austria
◯ Azerbaijan
◯ Bahamas
◯ Bahrain
◯ Bangladesh
◯ Barbados
◯ Belarus
◯ Belgium
◯ Belize
◯ Benin
◯ Bermuda
◯ Bhutan
◯ Bolivia (Plurinational State of)
◯ Bonaire, Sint Eustatius and Saba
◯ Bosnia and Herzegovina
◯ Botswana
◯ Bouvet Island
◯ Brazil
◯ British Indian Ocean Territory
◯ Brunei Darussalam
◯ Bulgaria
◯ Burkina Faso
◯ Burundi
◯ Cabo Verde
◯ Cambodia
◯ Cameroon
◯ Canada
◯ Cayman Islands
◯ Central African Republic
◯ Chad
◯ Chile
◯ China
◯ Christmas Island
◯ Cocos (Keeling) Islands
◯ Colombia
◯ Comoros
◯ Congo, Republic of the
◯ Congo, Democratic Republic of the
◯ Cook Islands
◯ Costa Rica
◯ Côte d'Ivoire
◯ Croatia
◯ Cuba
◯ Curaçao
◯ Cyprus
◯ Czechia
◯ Denmark
◯ Djibouti
◯ Dominica
◯ Dominican Republic
◯ Ecuador
◯ Egypt
◯ El Salvador
◯ Equatorial Guinea
◯ Eritrea

◯ Estonia
◯ Eswatini
◯ Ethiopia
◯ Falkland Islands (Malvinas)
◯ Faroe Islands
◯ Fiji
◯ Finland
◯ France
◯ French Guiana
◯ French Polynesia
◯ French Southern Territories
◯ Gabon
◯ Gambia
◯ Georgia
◯ Germany
◯ Ghana
◯ Gibraltar
◯ Greece
◯ Greenland
◯ Grenada
◯ Guadeloupe
◯ Guam
◯ Guatemala
◯ Guernsey
◯ Guinea
◯ Guinea-Bissau
◯ Guyana
◯ Haiti
◯ Heard Island and McDonald Islands
◯ Holy See
◯ Honduras
◯ Hong Kong
◯ Hungary
◯ Iceland
◯ India
◯ Indonesia
◯ Iran (Islamic Republic of)
◯ Iraq
◯ Ireland
◯ Isle of Man
◯ Israel
◯ Italy
◯ Jamaica
◯ Japan
◯ Jersey
◯ Jordan
◯ Kazakhstan
◯ Kenya
◯ Kiribati
◯ Korea (Democratic People's Republic of)
◯ Korea, Republic of
◯ Kuwait
◯ Kyrgyzstan
◯ Lao People's Democratic Republic
◯ Latvia
◯ Lebanon
◯ Lesotho
◯ Liberia
◯ Libya
◯ Liechtenstein
◯ Lithuania
◯ Luxembourg
◯ Macao
◯ Madagascar
◯ Malawi
◯ Malaysia
◯ Maldives
◯ Mali
◯ Malta
◯ Marshall Islands
◯ Martinique

◯ Mauritania
◯ Mauritius
◯ Mayotte
◯ Mexico
◯ Micronesia (Federated States of)
◯ Moldova, Republic of
◯ Monaco
◯ Mongolia
◯ Montenegro
◯ Montserrat
◯ Morocco
◯ Mozambique
◯ Myanmar
◯ Namibia
◯ Nauru
◯ Nepal
◯ Netherlands
◯ New Caledonia
◯ New Zealand
◯ Nicaragua
◯ Niger
◯ Nigeria
◯ Niue
◯ Norfolk Island
◯ North Macedonia
◯ Northern Mariana Islands
◯ Norway
◯ Oman
◯ Pakistan
◯ Palau
◯ Palestine, State of
◯ Panama
◯ Papua New Guinea
◯ Paraguay
◯ Peru
◯ Philippines
◯ Pitcairn
◯ Poland
◯ Portugal
◯ Puerto Rico
◯ Qatar
◯ Réunion
◯ Romania
◯ Russian Federation
◯ Rwanda
◯ Saint Barthélemy
◯ Saint Helena, Ascension and Tristan da Cunha
◯ Saint Kitts and Nevis
◯ Saint Lucia
◯ Saint Martin (French part)
◯ Saint Pierre and Miquelon
◯ Saint Vincent and the Grenadines
◯ Samoa
◯ San Marino
◯ Sao Tome and Principe
◯ Saudi Arabia
◯ Senegal
◯ Serbia
◯ Seychelles
◯ Sierra Leone
◯ Singapore
◯ Sint Maarten (Dutch part)
◯ Slovakia
◯ Slovenia
◯ Solomon Islands
◯ Somalia
◯ South Africa
◯ South Georgia and the South Sandwich Islands
◯ South Sudan
◯ Spain
◯ Sri Lanka

◯ Sudan
◯ Suriname
◯ Svalbard and Jan Mayen
◯ Sweden
◯ Switzerland
◯ Syrian Arab Republic
◯ Taiwan
◯ Tajikistan
◯ Tanzania, United Republic of
◯ Thailand
◯ Timor-Leste
◯ Togo
◯ Tokelau
◯ Tonga
◯ Trinidad and Tobago
◯ Tunisia
◯ Turkey
◯ Turkmenistan
◯ Turks and Caicos Islands
◯ Tuvalu
◯ Uganda
◯ Ukraine
◯ United Arab Emirates
◯ United Kingdom of Great Britain and Northern Ireland
◯ United States of America
◯ United States Minor Outlying Islands
◯ Uruguay
◯ Uzbekistan
◯ Vanuatu
◯ Venezuela (Bolivarian Republic of)
◯ Viet Nam
◯ Virgin Islands (British)
◯ Virgin Islands (U.S.)
◯ Wallis and Futuna
◯ Western Sahara
◯ Yemen
◯ Zambia
◯ Zimbabwe

(Start typing and country will be suggested for you)

Dirección residencial: País

◯ Afganistán
◯ Islas Aland
◯ Albania
◯ Argelia
◯ Samoa Americana
◯ Andorra
◯ Angola
◯ Anguila
◯ Antártida
◯ Antigua y Barbuda
◯ Argentina
◯ Armenia
◯ Aruba
◯ Australia
◯ Austria
◯ Azerbaiyán
◯ Bahamas
◯ Barein
◯ Bangladesh
◯ Barbados
◯ Bielorrusia
◯ Bélgica
◯ Belice
◯ Benin
◯ Islas Bermudas
◯ Bután
◯ Bolivia (Estado Plurinacional de)
◯ Bonaire, San Eustaquio y Saba
◯ Bosnia y Herzegovina
◯ Botsuana
◯ Isla Bouvet
◯ Brasil
◯ Territorio Británico del Océano Índico
◯ Brunei Darussalam
◯ Bulgaria
◯ Burkina Faso
◯ Burundi
◯ Cabo Verde
◯ Camboya
◯ Camerún
◯ Canadá
◯ Islas Caimán
◯ República Centroafricana
◯ Chad
◯ Chile
◯ China
◯ Isla de Navidad
◯ Islas Cocos (Keeling)
◯ Colombia
◯ Comoras
◯ Congo, República del
◯ Congo, República Democrática del
◯ Islas Cook
◯ Costa Rica
◯ Costa de Marfil
◯ Croacia
◯ Cuba
◯ Curazao
◯ Chipre
◯ Chequia
◯ Dinamarca
◯ Djibouti
◯ Dominica
◯ República Dominicana
◯ Ecuador
◯ Egipto
◯ El Salvador
◯ Guinea Ecuatorial
◯ Eritrea

◯ Estonia
◯ Eswatini
◯ Etiopía
◯ Islas Malvinas (Falkland Islands)
◯ Islas Faroe
◯ Fiyi
◯ Finlandia
◯ Francia
◯ Guayana Francesa
◯ Polinesia francesa
◯ Territorios Franceses del Sur
◯ Gabón
◯ Gambia
◯ Georgia
◯ Alemania
◯ Ghana
◯ Gibraltar
◯ Grecia
◯ Groenlandia
◯ Granada
◯ Guadalupe
◯ Guam
◯ Guatemala
◯ Guernsey
◯ Guinea
◯ Guinea-Bissau
◯ Guyana
◯ Haití
◯ Islas Heard y McDonald
◯ Santa Sede
◯ Honduras
◯ Hong Kong
◯ Hungría
◯ Islandia
◯ India
◯ Indonesia
◯ Irán (República Islámica de)
◯ Irak
◯ Irlanda
◯ Isla de Man
◯ Israel
◯ Italia
◯ Jamaica
◯ Japón
◯ Jersey
◯ Jordán
◯ Kazajstán
◯ Kenia
◯ Kiribati
◯ Corea (República Popular Democrática de)
◯ Corea, República de
◯ Kuwait
◯ Kirguistán
◯ República Democrática Popular Lao
◯ Letonia
◯ Líbano
◯ Lesoto
◯ Liberia
◯ Libia
◯ Liechtenstein
◯ Lituania
◯ Luxemburgo
◯ Macao
◯ Madagascar
◯ Malawi
◯ Malasia
◯ Maldivas
◯ Mali
◯ Malta
◯ Islas Marshall
◯ Martinica

◯ Mauritania
◯ Mauricio
◯ Mayotte
◯ México
◯ Micronesia (Estados Federados de)
◯ Moldavia, República de
◯ Mónaco
◯ Mongolia
◯ Montenegro
◯ Montserrat
◯ Marruecos
◯ Mozambique
◯ Myanmar
◯ Namibia
◯ Nauru
◯ Nepal
◯ Países Bajos
◯ Nueva Caledonia
◯ Nueva Zelanda
◯ Nicaragua
◯ Níger
◯ Nigeria
◯ Niue
◯ Isla Norfolk
◯ Macedonia del Norte
◯ Islas Marianas del Norte
◯ Noruega
◯ Omán
◯ Pakistán
◯ Palau
◯ Palestina, Estado de
◯ Panamá
◯ Papúa Nueva Guinea
◯ Paraguay
◯ Perú
◯ Filipinas
◯ Pitcairn
◯ Polonia
◯ Portugal
◯ Puerto Rico
◯ Katar
◯ Reunión
◯ Rumania
◯ Federación Rusa
◯ Ruanda
◯ San Bartolomé
◯ Santa Helena, Ascensión y Tristan da Cunha
◯ San Cristóbal y Nieves
◯ Santa Lucía
◯ San Martín (parte francesa)
◯ San Pedro y Miquelón
◯ San Vicente y las Granadinas
◯ Samoa
◯ San Marino
◯ Santo Tomé y Príncipe
◯ Arabia Saudita
◯ Senegal
◯ Serbia
◯ Seychelles
◯ Sierra Leona
◯ Singapur
◯ San Martín (parte holandesa)
◯ Eslovaquia
◯ Eslovenia
◯ Islas Salomón
◯ Somalia
◯ Sudáfrica
◯ Georgia del Sur y las Islas Sandwich del Sur
◯ Sudán del Sur
◯ España
◯ Sri Lanka

◯ Sudán
◯ Surinam
◯ Svalbard y Jan Mayen
◯ Suecia
◯ Suiza
◯ República Árabe Siria
◯ Taiwán
◯ Tayikistán
◯ Tanzania, República Unida de
◯ Tailandia
◯ Timor-Leste
◯ Togo
◯ Tokelau
◯ Tonga
◯ Trinidad y Tobago
◯ Túnez
◯ Turquía
◯ Turkmenistán
◯ Islas Turcas y Caicos
◯ Tuvalu
◯ Uganda
◯ Ucrania
◯ Emiratos Árabes Unidos
◯ Reino Unido de Gran Bretaña e Irlanda del Norte
◯ Estados Unidos de América
◯ Islas Ultramarinas Menores de los Estados Unidos
◯ Uruguay
◯ Uzbekistán
◯ Vanuatu
◯ Venezuela (República Bolivariana de)
◯ Vietnam
◯ Islas Vírgenes (británicas)
◯ Islas Vírgenes (EE. UU.)
◯ Wallis y Futuna
◯ Sahara Occidental
◯ Yemen
◯ Zambia
◯ Zimbabwe
(Comience a escribir y aparecerán sugerencias del país.)

Home address: Zipcode

____
(Ex: 98105)

Dirección residencial: Código postal

____
(Ex: 98105)

Home address: Zipcode/postal code

____

Dirección residencial: Código postal

____

Including yourself, how many people share your kitchen or living space?

◯ I live by myself
◯ 2 people
◯ 3 people
◯ 4 people
◯ 5 people
◯ 6 or more people

Incluyendo usted, ¿cuántas personas comparten su cocina o espacio habitable?	◯ Vivo solo<br>◯ 2 personas<br>◯ 3 personas<br>◯ 4 personas<br>◯ 5 personas<br>◯ 6 o más personas
Including yourself, how many people are in the room you sleep in?	◯ Just myself<br>◯ 2 people<br>◯ 3 people<br>◯ 4 people<br>◯ 5 people<br>◯ 6 or more people
Incluido usted, ¿cuántas personas hay en la habitación donde duerme?	◯ Solo yo<br>◯ 2 personas<br>◯ 3 personas<br>◯ 4 personas<br>◯ 5 personas<br>◯ 6 o más personas
What age groups of children stay in your living space? Select all that apply.	☐ No children<br>☐ Age 0-5 years<br>☐ Age 6-12 years<br>☐ Age 13-18 years
¿A qué grupos de edad pertenecen los niños que se viven en el espacio donde usted vive? Seleccione todas las que correspondan.	☐ No hay niños<br>☐ 0-5 años<br>☐ 6-12 años<br>☐ 13-18 años
What age groups of children stay in the room that you sleep in? Select all that apply.	☐ No children<br>☐ Age 0-5 years<br>☐ Age 6-12 years<br>☐ Age 13-18 years
¿A qué grupos de edad pertenecen los niños que duermen en el cuarto donde usted duerme? Seleccione todas las que correspondan.	☐ No hay niños<br>☐ 0-5 años<br>☐ 6-12 años<br>☐ 13-18 años
Do these children attend daycare or a child care center?	◯ Yes<br>◯ No
Asisten estos niños a una guardería o un centro de cuidado de niños?	◯ Sí<br>◯ No
Approximately how many hours per week do these children attend daycare or childcare?	______________________________
Aproximadamente por cuántas horas cada semana asisten estos niños a la guardería?	______________________________
Your Health	
Su salud	

In the past 7 days, has someone you live with been diagnosed with the flu by a medical professional?	◯ Yes<br>◯ No<br>◯ Do not know
En los últimos 7 días, ¿alguien con quien vive ha sido diagnosticado con gripe por un profesional médico?	◯ Sí<br>◯ No<br>◯ No lo sé
Have you sought clinical care for your current illness since it started?	◯ Yes - Doctor's office or Urgent Care<br>◯ Yes - Pharmacy (drugstore)<br>◯ Yes - Hospital or Emergency Department<br>◯ Yes - Other<br>◯ No
¿Ha buscado atención clínica para su enfermedad actual desde que comenzó?	◯ Sí - Consultorio médico o atención de urgencia<br>◯ Sí - Farmacia<br>◯ Sí - Hospital o departamento de emergencias<br>◯ Si - Otro<br>◯ No
Are you receiving an antiviral prescribed by a medical professional for the treatment or prevention of flu?	◯ No<br>◯ Yes; Oseltamivir (Tamiflu)<br>◯ Yes; Zanamivir (Relenza)<br>◯ Yes; Peramivir (Rapivab)<br>◯ Yes; Baloxavir (Xofluza)<br>◯ Yes, but I don't know which medication<br>◯ Do not know
Are you receiving an antibiotic prescribed by a medical professional for your current illness?	◯ No<br>◯ Yes; Zithromycin (Z-pack or Zithromax)<br>◯ Yes; Amoxicillin (Moxatag)<br>◯ Yes; Amoxicillin/Clavulanate (Augmentin)<br>◯ Yes; Levofloxacin (Levaquin)<br>◯ Yes; Moxifloxacin (Avelox)<br>◯ Yes, but I don't remember which antibiotic<br>◯ Yes, but my antibiotic is not listed<br>◯ Do not know
¿Está recibiendo un medicamento antivírico recetado por un profesional médico para el tratamiento o la prevención de la gripe?	◯ No<br>◯ Sí; Oseltamivir (Tamiflu)<br>◯ Sí; Zanamivir (Relenza)<br>◯ Sí; Peramivir (Rapivab)<br>◯ Sí; Baloxavir (Xofluza)<br>◯ Sí, pero no sé qué medicamento<br>◯ No lo sé
At my place of work, employees are encouraged to take time off or work from home if they are sick.	◯ I am not currently employed<br>◯ Yes, and I would be paid for hours missed<br>◯ Yes, but I would not be paid for hours missed ◯ No
En mi lugar de trabajo, se alienta a los empleados a no ir a trabajar o trabajar desde casa si están enfermos.	◯ Actualmente no trabajo<br>◯ Sí, y me pagarían las horas faltadas<br>◯ Sí, pero no me pagarían las horas faltadas<br>◯ No

What is your primary reason for being at [site_identifier_workplace] today?	◯ I am an employee<br>◯ I am a patient<br>◯ I am visiting a patient<br>◯ Other
¿Cuál es la razón principal por la que está en [site_identifier_workplace] hoy?	◯ Soy empleado<br>◯ Soy paciente<br>◯ Estoy visitando a un paciente<br>◯ Otra
Are you considered "essential personnel" for the City of Seattle?<br><br>Note: "Essential personnel" means employees designated by their management because their work directly supports efforts to maintain or restore public safety. This includes, but is not limited to: utility workers, roadway maintenance workers, police officers and firefighters.	◯ I am not employed by the City of Seattle<br>◯ I am considered Essential Personnel<br>◯ I am not considered Essential Personnel<br>◯ I am employed by the City of Seattle, but I don't know if I am Essential Personnel
What floor do you primarily work on in the Seattle Municipal Tower?<br><br>If you do not work in the Seattle Municipal Tower, you can type "NA".	__________________________________
How has your current illness affected your ability to do your regular activities (work, school, etc.)?	◯ Not at all<br>◯ A little bit<br>◯ Somewhat<br>◯ Quite a bit<br>◯ Very much
¿Cómo ha afectado su enfermedad actual en su capacidad para hacer sus actividades habituales (trabajo, escuela, etc.)?	◯ Nada<br>◯ Un poco<br>◯ Algo<br>◯ Bastante<br>◯ Mucho
Other than work and/or school, which of the following daily activities have been impacted by your current illness? Select all that apply.	☐ Running errands<br>☐ Exercising<br>☐ Socializing<br>☐ Volunteering<br>☐ Ability to take care of myself and/or family<br>☐ None of the above/ my activities have not been impacted
Aparte del trabajo y/o la escuela, ¿cuál de las siguientes actividades diarias han sido afectadas por su enfermedad actual? Seleccione todas las que correspondan.	☐ Hacer mandados<br>☐ Hacer ejercicio<br>☐ Socializar<br>☐ Trabajar como voluntario<br>☐ Capacidad para cuidarme a mí mismo o a mi familia<br>☐ Ninguna de las anteriores/ mis actividades no se han visto afectadas
Has your current illness kept you from doing any of the following? Select all that apply.	☐ Attending class<br>☐ Going to work<br>☐ Studying<br>☐ Performing well on an exam or written assignment<br>☐ None of the above/ my activities have not been impacted

¿Su enfermedad actual le ha impedido hacer alguna de las siguientes cosas? Seleccione todas las que correspondan.	☐ Asistir a clases<br>☐ Ir a trabajar<br>☐ Estudiar<br>☐ Sacar buenas calificaciones en un examen o tarea de redacción<br>☐ Ninguna de las anteriores/ mis actividades no se han visto afectadas
Behavioral Questions	
Preguntas de comportamiento	
Do you use any of the following products (either indoors or outdoors)? Select all that apply.	☐ Tobacco products (e.g. cigarettes, cigars, pipes)<br>☐ Electronic cigarettes/vapor pens<br>☐ None of the above<br>☐ Prefer not to say
¿Utiliza alguno de los siguientes productos (en interiores o exteriores)? Seleccione todas las que correspondan.	☐ Productos de tabaco (p. ej. cigarrillos, puros, pipas)<br>☐ Cigarrillos electrónicos/bolígrafos de vapor<br>☐ Ninguna de las anteriores respuestas<br>☐ Prefiero no decir
Does anyone in your shared living space use any of the following products (either indoors or outdoors)?<br>Select all that apply.	☐ Tobacco products (e.g. cigarettes, cigars, pipes)<br>☐ Electronic cigarettes/vapor pens<br>☐ None of the above<br>☐ Do not know<br>☐ Prefer not to say
¿Alguien en su espacio compartido de vivienda utiliza alguno de los siguientes productos (ya sea en interiores o exteriores)? Seleccione todas las que correspondan.	☐ Productos de tabaco (p. ej. cigarrillos, puros, pipas)<br>☐ Cigarrillos electrónicos/bolígrafos de vapor<br>☐ Ninguna de las anteriores respuestas<br>☐ No lo sé<br>☐ Prefiero no decir
Have you received this season's influenza (flu) vaccine (since July 1, 2019) ? This includes both flu mist nasal spray and the flu shot.	◯ Yes ◯ No ◯ Do not know
¿Ha recibido la vacuna contra la influenza (gripe) de esta temporada (a partir del 1 de julio de 2019)? Esto incluye tanto el aerosol nasal contra la gripe como la vacuna contra la gripe.	◯ Sí ◯ No ◯ No lo sé
What year did you get the flu shot or flu mist nasal spray this season (since July 1, 2019)?	◯ 2019<br>◯ 2020<br>◯ Do not know
¿En qué año recibió la vacuna contra la gripe o el aerosol nasal contra la gripe esta temporada (a partir del 1 de julio de 2019)?	◯ 2019<br>◯ 2020<br>◯ No lo sé

What month did you get the flu shot or flu mist nasal spray this season (since July 1, 2019)?	◯ January<br>◯ February<br>◯ March<br>◯ April<br>◯ May<br>◯ June<br>◯ July<br>◯ August<br>◯ September<br>◯ October<br>◯ November<br>◯ December<br>◯ Do not know
¿En qué mes recibió la vacuna contra la gripe o el aerosol nasal contra la gripe esta temporada (a partir del 1 de julio de 2019)?	◯ Enero<br>◯ Febrero<br>◯ Marzo<br>◯ Abril<br>◯ Mayo<br>◯ Junio<br>◯ Julio<br>◯ Agosto<br>◯ Septiembre<br>◯ Octubre<br>◯ Noviembre<br>◯ Diciembre<br>◯ No lo sé
How did you receive the flu vaccine this season (since July 1, 2019)?	◯ Injection (flu shot) ◯ Nasal spray (flu mist)
¿Cómo recibió la vacuna contra la gripe esta temporada (a partir del 1 de julio de 2019)?	◯ Inyección (vacuna contra la gripe)<br>◯ Aerosol nasal (aerosol contra la gripe)
Where did you receive the flu vaccine this season (since July 1, 2019)?	◯ Medical clinic or hospital<br>◯ Pharmacy or drugstore<br>◯ Workplace<br>◯ School<br>◯ Other
¿Dónde recibió la vacuna contra la gripe esta temporada (a partir del 1 de julio de 2019)?	◯ Clínica médica o hospital<br>◯ Farmacia<br>◯ Lugar de trabajo<br>◯ Escuela<br>◯ Otro
What is the primary reason why you have not received the flu vaccine this season (since July 1, 2019)?	◯ I plan to get the flu vaccine<br>◯ Do not have time to get vaccinated<br>◯ Not required for work or school<br>◯ Not recommended by a doctor or healthcare worker<br>◯ Not covered by health insurance<br>◯ Not offered at a convenient location<br>◯ I am not worried about getting sick with the flu<br>◯ Concerns about vaccine safety or effectiveness<br>◯ None of the above<br>◯ Prefer not to say

¿Cuál es la razón principal por la que no ha recibido la vacuna contra la gripe esta temporada (a partir del 1 de julio de 2019)?	◯ Planeo vacunarme contra la gripe<br>◯ No tengo tiempo para vacunarme<br>◯ No es necesario para el trabajo o la escuela<br>◯ No es lo recomendado por un médico o trabajador de la salud<br>◯ No está cubierto por el seguro médico<br>◯ No la ofrecen en un lugar conveniente<br>◯ No me preocupa enfermarme de gripe<br>◯ Inquietudes sobre la seguridad o eficacia de la vacuna<br>◯ Ninguna de las anteriores respuestas<br>◯ Prefiero no decir
Did you receive the flu vaccine last season?<br>(July 1, 2018 - July 1, 2019)	◯ Yes ◯ No
¿Recibió la vacuna contra la gripe la temporada pasada?<br>(1 de julio de 2018 - 1 de julio de 2019)	◯ Sí ◯ No
Have you ever been told by a healthcare provider that you have one of the following medical conditions? Select all that apply.	☐ Asthma or reactive airway disease<br>☐ COPD/emphysema<br>☐ Chronic bronchitis<br>☐ Cancer<br>☐ Diabetes<br>☐ Heart disease (heart failure or heart attack)<br>☐ None of these conditions<br>☐ Do not know<br>☐ Prefer not to say
¿Alguna vez le ha dicho un proveedor de atención médica que tiene una de las siguientes afecciones médicas? Seleccione todas las que correspondan.	☐ Asma o enfermedad reactiva de las vías respiratorias<br>☐ COPD/enfisema<br>☐ Bronquitis crónica<br>☐ Cáncer<br>☐ Diabetes<br>☐ Enfermedad cardíaca (insuficiencia cardíaca o ataque cardíaco)<br>☐ Ninguna de estas afecciones<br>☐ No lo sé<br>☐ Prefiero no decir
Recent Travel	
Viajes recientes	
In the past 7 days, have you visited a country other than the US?	◯ Yes<br>◯ No - I have not traveled outside the US
En los últimos 7 días, ¿ha estado en un país aparte de los EE. UU.?	◯ Sí<br>◯ No - No he viajado fuera de los EE. UU.

Country 1 visited

- ◯ Afghanistan
- ◯ Åland Islands
- ◯ Albania
- ◯ Algeria
- ◯ American Samoa
- ◯ Andorra
- ◯ Angola
- ◯ Anguilla
- ◯ Antarctica
- ◯ Antigua and Barbuda
- ◯ Argentina
- ◯ Armenia
- ◯ Aruba
- ◯ Australia
- ◯ Austria
- ◯ Azerbaijan
- ◯ Bahamas
- ◯ Bahrain
- ◯ Bangladesh
- ◯ Barbados
- ◯ Belarus
- ◯ Belgium
- ◯ Belize
- ◯ Benin
- ◯ Bermuda
- ◯ Bhutan
- ◯ Bolivia (Plurinational State of)
- ◯ Bonaire, Sint Eustatius and Saba
- ◯ Bosnia and Herzegovina
- ◯ Botswana
- ◯ Bouvet Island
- ◯ Brazil
- ◯ British Indian Ocean Territory
- ◯ Brunei Darussalam
- ◯ Bulgaria
- ◯ Burkina Faso
- ◯ Burundi
- ◯ Cabo Verde
- ◯ Cambodia
- ◯ Cameroon
- ◯ Canada
- ◯ Cayman Islands
- ◯ Central African Republic
- ◯ Chad
- ◯ Chile
- ◯ China
- ◯ Christmas Island
- ◯ Cocos (Keeling) Islands
- ◯ Colombia
- ◯ Comoros
- ◯ Congo, Republic of the
- ◯ Congo, Democratic Republic of the
- ◯ Cook Islands
- ◯ Costa Rica
- ◯ Côte d'Ivoire
- ◯ Croatia
- ◯ Cuba
- ◯ Curaçao
- ◯ Cyprus
- ◯ Czechia
- ◯ Denmark
- ◯ Djibouti
- ◯ Dominica
- ◯ Dominican Republic
- ◯ Ecuador
- ◯ Egypt
- ◯ El Salvador
- ◯ Equatorial Guinea
- ◯ Eritrea

◯ Estonia
◯ Eswatini
◯ Ethiopia
◯ Falkland Islands (Malvinas)
◯ Faroe Islands
◯ Fiji
◯ Finland
◯ France
◯ French Guiana
◯ French Polynesia
◯ French Southern Territories
◯ Gabon
◯ Gambia
◯ Georgia
◯ Germany
◯ Ghana
◯ Gibraltar
◯ Greece
◯ Greenland
◯ Grenada
◯ Guadeloupe
◯ Guam
◯ Guatemala
◯ Guernsey
◯ Guinea
◯ Guinea-Bissau
◯ Guyana
◯ Haiti
◯ Heard Island and McDonald Islands
◯ Holy See
◯ Honduras
◯ Hong Kong
◯ Hungary
◯ Iceland
◯ India
◯ Indonesia
◯ Iran (Islamic Republic of)
◯ Iraq
◯ Ireland
◯ Isle of Man
◯ Israel
◯ Italy
◯ Jamaica
◯ Japan
◯ Jersey
◯ Jordan
◯ Kazakhstan
◯ Kenya
◯ Kiribati
◯ Korea (Democratic People's Republic of)
◯ Korea, Republic of
◯ Kuwait
◯ Kyrgyzstan
◯ Lao People's Democratic Republic
◯ Latvia
◯ Lebanon
◯ Lesotho
◯ Liberia
◯ Libya
◯ Liechtenstein
◯ Lithuania
◯ Luxembourg
◯ Macao
◯ Madagascar
◯ Malawi
◯ Malaysia
◯ Maldives
◯ Mali
◯ Malta
◯ Marshall Islands
◯ Martinique

◯ Mauritania
◯ Mauritius
◯ Mayotte
◯ Mexico
◯ Micronesia (Federated States of)
◯ Moldova, Republic of
◯ Monaco
◯ Mongolia
◯ Montenegro
◯ Montserrat
◯ Morocco
◯ Mozambique
◯ Myanmar
◯ Namibia
◯ Nauru
◯ Nepal
◯ Netherlands
◯ New Caledonia
◯ New Zealand
◯ Nicaragua
◯ Niger
◯ Nigeria
◯ Niue
◯ Norfolk Island
◯ North Macedonia
◯ Northern Mariana Islands
◯ Norway
◯ Oman
◯ Pakistan
◯ Palau
◯ Palestine, State of
◯ Panama
◯ Papua New Guinea
◯ Paraguay
◯ Peru
◯ Philippines
◯ Pitcairn
◯ Poland
◯ Portugal
◯ Puerto Rico
◯ Qatar
◯ Réunion
◯ Romania
◯ Russian Federation
◯ Rwanda
◯ Saint Barthélemy
◯ Saint Helena, Ascension and Tristan da Cunha
◯ Saint Kitts and Nevis
◯ Saint Lucia
◯ Saint Martin (French part)
◯ Saint Pierre and Miquelon
◯ Saint Vincent and the Grenadines
◯ Samoa
◯ San Marino
◯ Sao Tome and Principe
◯ Saudi Arabia
◯ Senegal
◯ Serbia
◯ Seychelles
◯ Sierra Leone
◯ Singapore
◯ Sint Maarten (Dutch part)
◯ Slovakia
◯ Slovenia
◯ Solomon Islands
◯ Somalia
◯ South Africa
◯ South Georgia and the South Sandwich Islands
◯ South Sudan
◯ Spain
◯ Sri Lanka

◯ Sudan
◯ Suriname
◯ Svalbard and Jan Mayen
◯ Sweden
◯ Switzerland
◯ Syrian Arab Republic
◯ Taiwan
◯ Tajikistan
◯ Tanzania, United Republic of
◯ Thailand
◯ Timor-Leste
◯ Togo
◯ Tokelau
◯ Tonga
◯ Trinidad and Tobago
◯ Tunisia
◯ Turkey
◯ Turkmenistan
◯ Turks and Caicos Islands
◯ Tuvalu
◯ Uganda
◯ Ukraine
◯ United Arab Emirates
◯ United Kingdom of Great Britain and Northern Ireland
◯ United States of America
◯ United States Minor Outlying Islands
◯ Uruguay
◯ Uzbekistan
◯ Vanuatu
◯ Venezuela (Bolivarian Republic of)
◯ Viet Nam
◯ Virgin Islands (British)
◯ Virgin Islands (U.S.)
◯ Wallis and Futuna
◯ Western Sahara
◯ Yemen
◯ Zambia
◯ Zimbabwe

(Start typing and the country will be suggested)

País 1 en el que estuvo

- ◯ Afganistán
- ◯ Islas Aland
- ◯ Albania
- ◯ Argelia
- ◯ Samoa Americana
- ◯ Andorra
- ◯ Angola
- ◯ Anguila
- ◯ Antártida
- ◯ Antigua y Barbuda
- ◯ Argentina
- ◯ Armenia
- ◯ Aruba
- ◯ Australia
- ◯ Austria
- ◯ Azerbaiyán
- ◯ Bahamas
- ◯ Barein
- ◯ Bangladesh
- ◯ Barbados
- ◯ Bielorrusia
- ◯ Bélgica
- ◯ Belice
- ◯ Benin
- ◯ Islas Bermudas
- ◯ Bután
- ◯ Bolivia (Estado Plurinacional de)
- ◯ Bonaire, San Eustaquio y Saba
- ◯ Bosnia y Herzegovina
- ◯ Botsuana
- ◯ Isla Bouvet
- ◯ Brasil
- ◯ Territorio Británico del Océano Índico
- ◯ Brunei Darussalam
- ◯ Bulgaria
- ◯ Burkina Faso
- ◯ Burundi
- ◯ Cabo Verde
- ◯ Camboya
- ◯ Camerún
- ◯ Canadá
- ◯ Islas Caimán
- ◯ República Centroafricana
- ◯ Chad
- ◯ Chile
- ◯ China
- ◯ Isla de Navidad
- ◯ Islas Cocos (Keeling)
- ◯ Colombia
- ◯ Comoras
- ◯ Congo, República del
- ◯ Congo, República Democrática del
- ◯ Islas Cook
- ◯ Costa Rica
- ◯ Costa de Marfil
- ◯ Croacia
- ◯ Cuba
- ◯ Curazao
- ◯ Chipre
- ◯ Chequia
- ◯ Dinamarca
- ◯ Djibouti
- ◯ Dominica
- ◯ República Dominicana
- ◯ Ecuador
- ◯ Egipto
- ◯ El Salvador
- ◯ Guinea Ecuatorial
- ◯ Eritrea

◯ Estonia
◯ Eswatini
◯ Etiopía
◯ Islas Malvinas (Falkland Islands)
◯ Islas Faroe
◯ Fiyi
◯ Finlandia
◯ Francia
◯ Guayana Francesa
◯ Polinesia francesa
◯ Territorios Franceses del Sur
◯ Gabón
◯ Gambia
◯ Georgia
◯ Alemania
◯ Ghana
◯ Gibraltar
◯ Grecia
◯ Groenlandia
◯ Granada
◯ Guadalupe
◯ Guam
◯ Guatemala
◯ Guernsey
◯ Guinea
◯ Guinea-Bissau
◯ Guyana
◯ Haití
◯ Islas Heard y McDonald
◯ Santa Sede
◯ Honduras
◯ Hong Kong
◯ Hungría
◯ Islandia
◯ India
◯ Indonesia
◯ Irán (República Islámica de)
◯ Irak
◯ Irlanda
◯ Isla de Man
◯ Israel
◯ Italia
◯ Jamaica
◯ Japón
◯ Jersey
◯ Jordán
◯ Kazajstán
◯ Kenia
◯ Kiribati
◯ Corea (República Popular Democrática de)
◯ Corea, República de
◯ Kuwait
◯ Kirguistán
◯ República Democrática Popular Lao
◯ Letonia
◯ Líbano
◯ Lesoto
◯ Liberia
◯ Libia
◯ Liechtenstein
◯ Lituania
◯ Luxemburgo
◯ Macao
◯ Madagascar
◯ Malawi
◯ Malasia
◯ Maldivas
◯ Mali
◯ Malta
◯ Islas Marshall
◯ Martinica

- ◯ Mauritania
- ◯ Mauricio
- ◯ Mayotte
- ◯ México
- ◯ Micronesia (Estados Federados de)
- ◯ Moldavia, República de
- ◯ Mónaco
- ◯ Mongolia
- ◯ Montenegro
- ◯ Montserrat
- ◯ Marruecos
- ◯ Mozambique
- ◯ Myanmar
- ◯ Namibia
- ◯ Nauru
- ◯ Nepal
- ◯ Países Bajos
- ◯ Nueva Caledonia
- ◯ Nueva Zelanda
- ◯ Nicaragua
- ◯ Níger
- ◯ Nigeria
- ◯ Niue
- ◯ Isla Norfolk
- ◯ Macedonia del Norte
- ◯ Islas Marianas del Norte
- ◯ Noruega
- ◯ Omán
- ◯ Pakistán
- ◯ Palau
- ◯ Palestina, Estado de
- ◯ Panamá
- ◯ Papúa Nueva Guinea
- ◯ Paraguay
- ◯ Perú
- ◯ Filipinas
- ◯ Pitcairn
- ◯ Polonia
- ◯ Portugal
- ◯ Puerto Rico
- ◯ Katar
- ◯ Reunión
- ◯ Rumania
- ◯ Federación Rusa
- ◯ Ruanda
- ◯ San Bartolomé
- ◯ Santa Helena, Ascensión y Tristan da Cunha
- ◯ San Cristóbal y Nieves
- ◯ Santa Lucía
- ◯ San Martín (parte francesa)
- ◯ San Pedro y Miquelón
- ◯ San Vicente y las Granadinas
- ◯ Samoa
- ◯ San Marino
- ◯ Santo Tomé y Príncipe
- ◯ Arabia Saudita
- ◯ Senegal
- ◯ Serbia
- ◯ Seychelles
- ◯ Sierra Leona
- ◯ Singapur
- ◯ San Martín (parte holandesa)
- ◯ Eslovaquia
- ◯ Eslovenia
- ◯ Islas Salomón
- ◯ Somalia
- ◯ Sudáfrica
- ◯ Georgia del Sur y las Islas Sandwich del Sur
- ◯ Sudán del Sur
- ◯ España
- ◯ Sri Lanka

◯ Sudán
◯ Surinam
◯ Svalbard y Jan Mayen
◯ Suecia
◯ Suiza
◯ República Árabe Siria
◯ Taiwán
◯ Tayikistán
◯ Tanzania, República Unida de
◯ Tailandia
◯ Timor-Leste
◯ Togo
◯ Tokelau
◯ Tonga
◯ Trinidad y Tobago
◯ Túnez
◯ Turquía
◯ Turkmenistán
◯ Islas Turcas y Caicos
◯ Tuvalu
◯ Uganda
◯ Ucrania
◯ Emiratos Árabes Unidos
◯ Reino Unido de Gran Bretaña e Irlanda del Norte
◯ Estados Unidos de América
◯ Islas Ultramarinas Menores de los Estados Unidos
◯ Uruguay
◯ Uzbekistán
◯ Vanuatu
◯ Venezuela (República Bolivariana de)
◯ Vietnam
◯ Islas Vírgenes (británicas)
◯ Islas Vírgenes (EE. UU.)
◯ Wallis y Futuna
◯ Sahara Occidental
◯ Yemen
◯ Zambia
◯ Zimbabwe

(Comience a escribir y aparecerán sugerencias del país.)

Country 2 visited

◯ Afghanistan
◯ Åland Islands
◯ Albania
◯ Algeria
◯ American Samoa
◯ Andorra
◯ Angola
◯ Anguilla
◯ Antarctica
◯ Antigua and Barbuda
◯ Argentina
◯ Armenia
◯ Aruba
◯ Australia
◯ Austria
◯ Azerbaijan
◯ Bahamas
◯ Bahrain
◯ Bangladesh
◯ Barbados
◯ Belarus
◯ Belgium
◯ Belize
◯ Benin
◯ Bermuda
◯ Bhutan
◯ Bolivia (Plurinational State of)
◯ Bonaire, Sint Eustatius and Saba
◯ Bosnia and Herzegovina
◯ Botswana
◯ Bouvet Island
◯ Brazil
◯ British Indian Ocean Territory
◯ Brunei Darussalam
◯ Bulgaria
◯ Burkina Faso
◯ Burundi
◯ Cabo Verde
◯ Cambodia
◯ Cameroon
◯ Canada
◯ Cayman Islands
◯ Central African Republic
◯ Chad
◯ Chile
◯ China
◯ Christmas Island
◯ Cocos (Keeling) Islands
◯ Colombia
◯ Comoros
◯ Congo, Republic of the
◯ Congo, Democratic Republic of the
◯ Cook Islands
◯ Costa Rica
◯ Côte d'Ivoire
◯ Croatia
◯ Cuba
◯ Curaçao
◯ Cyprus
◯ Czechia
◯ Denmark
◯ Djibouti
◯ Dominica
◯ Dominican Republic
◯ Ecuador
◯ Egypt
◯ El Salvador
◯ Equatorial Guinea
◯ Eritrea

- ◯ Estonia
- ◯ Eswatini
- ◯ Ethiopia
- ◯ Falkland Islands (Malvinas)
- ◯ Faroe Islands
- ◯ Fiji
- ◯ Finland
- ◯ France
- ◯ French Guiana
- ◯ French Polynesia
- ◯ French Southern Territories
- ◯ Gabon
- ◯ Gambia
- ◯ Georgia
- ◯ Germany
- ◯ Ghana
- ◯ Gibraltar
- ◯ Greece
- ◯ Greenland
- ◯ Grenada
- ◯ Guadeloupe
- ◯ Guam
- ◯ Guatemala
- ◯ Guernsey
- ◯ Guinea
- ◯ Guinea-Bissau
- ◯ Guyana
- ◯ Haiti
- ◯ Heard Island and McDonald Islands
- ◯ Holy See
- ◯ Honduras
- ◯ Hong Kong
- ◯ Hungary
- ◯ Iceland
- ◯ India
- ◯ Indonesia
- ◯ Iran (Islamic Republic of)
- ◯ Iraq
- ◯ Ireland
- ◯ Isle of Man
- ◯ Israel
- ◯ Italy
- ◯ Jamaica
- ◯ Japan
- ◯ Jersey
- ◯ Jordan
- ◯ Kazakhstan
- ◯ Kenya
- ◯ Kiribati
- ◯ Korea (Democratic People's Republic of)
- ◯ Korea, Republic of
- ◯ Kuwait
- ◯ Kyrgyzstan
- ◯ Lao People's Democratic Republic
- ◯ Latvia
- ◯ Lebanon
- ◯ Lesotho
- ◯ Liberia
- ◯ Libya
- ◯ Liechtenstein
- ◯ Lithuania
- ◯ Luxembourg
- ◯ Macao
- ◯ Madagascar
- ◯ Malawi
- ◯ Malaysia
- ◯ Maldives
- ◯ Mali
- ◯ Malta
- ◯ Marshall Islands
- ◯ Martinique

◯ Mauritania
◯ Mauritius
◯ Mayotte
◯ Mexico
◯ Micronesia (Federated States of)
◯ Moldova, Republic of
◯ Monaco
◯ Mongolia
◯ Montenegro
◯ Montserrat
◯ Morocco
◯ Mozambique
◯ Myanmar
◯ Namibia
◯ Nauru
◯ Nepal
◯ Netherlands
◯ New Caledonia
◯ New Zealand
◯ Nicaragua
◯ Niger
◯ Nigeria
◯ Niue
◯ Norfolk Island
◯ North Macedonia
◯ Northern Mariana Islands
◯ Norway
◯ Oman
◯ Pakistan
◯ Palau
◯ Palestine, State of
◯ Panama
◯ Papua New Guinea
◯ Paraguay
◯ Peru
◯ Philippines
◯ Pitcairn
◯ Poland
◯ Portugal
◯ Puerto Rico
◯ Qatar
◯ Réunion
◯ Romania
◯ Russian Federation
◯ Rwanda
◯ Saint Barthélemy
◯ Saint Helena, Ascension and Tristan da Cunha
◯ Saint Kitts and Nevis
◯ Saint Lucia
◯ Saint Martin (French part)
◯ Saint Pierre and Miquelon
◯ Saint Vincent and the Grenadines
◯ Samoa
◯ San Marino
◯ Sao Tome and Principe
◯ Saudi Arabia
◯ Senegal
◯ Serbia
◯ Seychelles
◯ Sierra Leone
◯ Singapore
◯ Sint Maarten (Dutch part)
◯ Slovakia
◯ Slovenia
◯ Solomon Islands
◯ Somalia
◯ South Africa
◯ South Georgia and the South Sandwich Islands
◯ South Sudan
◯ Spain
◯ Sri Lanka

◯ Sudan
◯ Suriname
◯ Svalbard and Jan Mayen
◯ Sweden
◯ Switzerland
◯ Syrian Arab Republic
◯ Taiwan
◯ Tajikistan
◯ Tanzania, United Republic of
◯ Thailand
◯ Timor-Leste
◯ Togo
◯ Tokelau
◯ Tonga
◯ Trinidad and Tobago
◯ Tunisia
◯ Turkey
◯ Turkmenistan
◯ Turks and Caicos Islands
◯ Tuvalu
◯ Uganda
◯ Ukraine
◯ United Arab Emirates
◯ United Kingdom of Great Britain and Northern Ireland
◯ United States of America
◯ United States Minor Outlying Islands
◯ Uruguay
◯ Uzbekistan
◯ Vanuatu
◯ Venezuela (Bolivarian Republic of)
◯ Viet Nam
◯ Virgin Islands (British)
◯ Virgin Islands (U.S.)
◯ Wallis and Futuna
◯ Western Sahara
◯ Yemen
◯ Zambia
◯ Zimbabwe

(Start typing and the country will be suggested. If you only visited 1 country, move to the next question)

País 2 en el que estuvo

◯ Afganistán
◯ Islas Aland
◯ Albania
◯ Argelia
◯ Samoa Americana
◯ Andorra
◯ Angola
◯ Anguila
◯ Antártida
◯ Antigua y Barbuda
◯ Argentina
◯ Armenia
◯ Aruba
◯ Australia
◯ Austria
◯ Azerbaiyán
◯ Bahamas
◯ Barein
◯ Bangladesh
◯ Barbados
◯ Bielorrusia
◯ Bélgica
◯ Belice
◯ Benin
◯ Islas Bermudas
◯ Bután
◯ Bolivia (Estado Plurinacional de)
◯ Bonaire, San Eustaquio y Saba
◯ Bosnia y Herzegovina
◯ Botsuana
◯ Isla Bouvet
◯ Brasil
◯ Territorio Británico del Océano Índico
◯ Brunei Darussalam
◯ Bulgaria
◯ Burkina Faso
◯ Burundi
◯ Cabo Verde
◯ Camboya
◯ Camerún
◯ Canadá
◯ Islas Caimán
◯ República Centroafricana
◯ Chad
◯ Chile
◯ China
◯ Isla de Navidad
◯ Islas Cocos (Keeling)
◯ Colombia
◯ Comoras
◯ Congo, República del
◯ Congo, República Democrática del
◯ Islas Cook
◯ Costa Rica
◯ Costa de Marfil
◯ Croacia
◯ Cuba
◯ Curazao
◯ Chipre
◯ Chequia
◯ Dinamarca
◯ Djibouti
◯ Dominica
◯ República Dominicana
◯ Ecuador
◯ Egipto
◯ El Salvador
◯ Guinea Ecuatorial
◯ Eritrea

◯ Estonia
◯ Eswatini
◯ Etiopía
◯ Islas Malvinas (Falkland Islands)
◯ Islas Faroe
◯ Fiyi
◯ Finlandia
◯ Francia
◯ Guayana Francesa
◯ Polinesia francesa
◯ Territorios Franceses del Sur
◯ Gabón
◯ Gambia
◯ Georgia
◯ Alemania
◯ Ghana
◯ Gibraltar
◯ Grecia
◯ Groenlandia
◯ Granada
◯ Guadalupe
◯ Guam
◯ Guatemala
◯ Guernsey
◯ Guinea
◯ Guinea-Bissau
◯ Guyana
◯ Haití
◯ Islas Heard y McDonald
◯ Santa Sede
◯ Honduras
◯ Hong Kong
◯ Hungría
◯ Islandia
◯ India
◯ Indonesia
◯ Irán (República Islámica de)
◯ Irak
◯ Irlanda
◯ Isla de Man
◯ Israel
◯ Italia
◯ Jamaica
◯ Japón
◯ Jersey
◯ Jordán
◯ Kazajstán
◯ Kenia
◯ Kiribati
◯ Corea (República Popular Democrática de)
◯ Corea, República de
◯ Kuwait
◯ Kirguistán
◯ República Democrática Popular Lao
◯ Letonia
◯ Líbano
◯ Lesoto
◯ Liberia
◯ Libia
◯ Liechtenstein
◯ Lituania
◯ Luxemburgo
◯ Macao
◯ Madagascar
◯ Malawi
◯ Malasia
◯ Maldivas
◯ Mali
◯ Malta
◯ Islas Marshall
◯ Martinica

◯ Mauritania
◯ Mauricio
◯ Mayotte
◯ México
◯ Micronesia (Estados Federados de)
◯ Moldavia, República de
◯ Mónaco
◯ Mongolia
◯ Montenegro
◯ Montserrat
◯ Marruecos
◯ Mozambique
◯ Myanmar
◯ Namibia
◯ Nauru
◯ Nepal
◯ Países Bajos
◯ Nueva Caledonia
◯ Nueva Zelanda
◯ Nicaragua
◯ Níger
◯ Nigeria
◯ Niue
◯ Isla Norfolk
◯ Macedonia del Norte
◯ Islas Marianas del Norte
◯ Noruega
◯ Omán
◯ Pakistán
◯ Palau
◯ Palestina, Estado de
◯ Panamá
◯ Papúa Nueva Guinea
◯ Paraguay
◯ Perú
◯ Filipinas
◯ Pitcairn
◯ Polonia
◯ Portugal
◯ Puerto Rico
◯ Katar
◯ Reunión
◯ Rumania
◯ Federación Rusa
◯ Ruanda
◯ San Bartolomé
◯ Santa Helena, Ascensión y Tristan da Cunha
◯ San Cristóbal y Nieves
◯ Santa Lucía
◯ San Martín (parte francesa)
◯ San Pedro y Miquelón
◯ San Vicente y las Granadinas
◯ Samoa
◯ San Marino
◯ Santo Tomé y Príncipe
◯ Arabia Saudita
◯ Senegal
◯ Serbia
◯ Seychelles
◯ Sierra Leona
◯ Singapur
◯ San Martín (parte holandesa)
◯ Eslovaquia
◯ Eslovenia
◯ Islas Salomón
◯ Somalia
◯ Sudáfrica
◯ Georgia del Sur y las Islas Sandwich del Sur
◯ Sudán del Sur
◯ España
◯ Sri Lanka

◯ Sudán
◯ Surinam
◯ Svalbard y Jan Mayen
◯ Suecia
◯ Suiza
◯ República Árabe Siria
◯ Taiwán
◯ Tayikistán
◯ Tanzania, República Unida de
◯ Tailandia
◯ Timor-Leste
◯ Togo
◯ Tokelau
◯ Tonga
◯ Trinidad y Tobago
◯ Túnez
◯ Turquía
◯ Turkmenistán
◯ Islas Turcas y Caicos
◯ Tuvalu
◯ Uganda
◯ Ucrania
◯ Emiratos Árabes Unidos
◯ Reino Unido de Gran Bretaña e Irlanda del Norte
◯ Estados Unidos de América
◯ Islas Ultramarinas Menores de los Estados Unidos
◯ Uruguay
◯ Uzbekistán
◯ Vanuatu
◯ Venezuela (República Bolivariana de)
◯ Vietnam
◯ Islas Vírgenes (británicas)
◯ Islas Vírgenes (EE. UU.)
◯ Wallis y Futuna
◯ Sahara Occidental
◯ Yemen
◯ Zambia
◯ Zimbabwe

(Comience a escribir y aparecerán sugerencias del país. Si solo fue a 1 país, pase a la siguiente pregunta.)

Country 3 visited

- ◯ Afghanistan
- ◯ Åland Islands
- ◯ Albania
- ◯ Algeria
- ◯ American Samoa
- ◯ Andorra
- ◯ Angola
- ◯ Anguilla
- ◯ Antarctica
- ◯ Antigua and Barbuda
- ◯ Argentina
- ◯ Armenia
- ◯ Aruba
- ◯ Australia
- ◯ Austria
- ◯ Azerbaijan
- ◯ Bahamas
- ◯ Bahrain
- ◯ Bangladesh
- ◯ Barbados
- ◯ Belarus
- ◯ Belgium
- ◯ Belize
- ◯ Benin
- ◯ Bermuda
- ◯ Bhutan
- ◯ Bolivia (Plurinational State of)
- ◯ Bonaire, Sint Eustatius and Saba
- ◯ Bosnia and Herzegovina
- ◯ Botswana
- ◯ Bouvet Island
- ◯ Brazil
- ◯ British Indian Ocean Territory
- ◯ Brunei Darussalam
- ◯ Bulgaria
- ◯ Burkina Faso
- ◯ Burundi
- ◯ Cabo Verde
- ◯ Cambodia
- ◯ Cameroon
- ◯ Canada
- ◯ Cayman Islands
- ◯ Central African Republic
- ◯ Chad
- ◯ Chile
- ◯ China
- ◯ Christmas Island
- ◯ Cocos (Keeling) Islands
- ◯ Colombia
- ◯ Comoros
- ◯ Congo, Republic of the
- ◯ Congo, Democratic Republic of the
- ◯ Cook Islands
- ◯ Costa Rica
- ◯ Côte d'Ivoire
- ◯ Croatia
- ◯ Cuba
- ◯ Curaçao
- ◯ Cyprus
- ◯ Czechia
- ◯ Denmark
- ◯ Djibouti
- ◯ Dominica
- ◯ Dominican Republic
- ◯ Ecuador
- ◯ Egypt
- ◯ El Salvador
- ◯ Equatorial Guinea
- ◯ Eritrea

◯ Estonia
◯ Eswatini
◯ Ethiopia
◯ Falkland Islands (Malvinas)
◯ Faroe Islands
◯ Fiji
◯ Finland
◯ France
◯ French Guiana
◯ French Polynesia
◯ French Southern Territories
◯ Gabon
◯ Gambia
◯ Georgia
◯ Germany
◯ Ghana
◯ Gibraltar
◯ Greece
◯ Greenland
◯ Grenada
◯ Guadeloupe
◯ Guam
◯ Guatemala
◯ Guernsey
◯ Guinea
◯ Guinea-Bissau
◯ Guyana
◯ Haiti
◯ Heard Island and McDonald Islands
◯ Holy See
◯ Honduras
◯ Hong Kong
◯ Hungary
◯ Iceland
◯ India
◯ Indonesia
◯ Iran (Islamic Republic of)
◯ Iraq
◯ Ireland
◯ Isle of Man
◯ Israel
◯ Italy
◯ Jamaica
◯ Japan
◯ Jersey
◯ Jordan
◯ Kazakhstan
◯ Kenya
◯ Kiribati
◯ Korea (Democratic People's Republic of)
◯ Korea, Republic of
◯ Kuwait
◯ Kyrgyzstan
◯ Lao People's Democratic Republic
◯ Latvia
◯ Lebanon
◯ Lesotho
◯ Liberia
◯ Libya
◯ Liechtenstein
◯ Lithuania
◯ Luxembourg
◯ Macao
◯ Madagascar
◯ Malawi
◯ Malaysia
◯ Maldives
◯ Mali
◯ Malta
◯ Marshall Islands
◯ Martinique

◯ Mauritania
◯ Mauritius
◯ Mayotte
◯ Mexico
◯ Micronesia (Federated States of)
◯ Moldova, Republic of
◯ Monaco
◯ Mongolia
◯ Montenegro
◯ Montserrat
◯ Morocco
◯ Mozambique
◯ Myanmar
◯ Namibia
◯ Nauru
◯ Nepal
◯ Netherlands
◯ New Caledonia
◯ New Zealand
◯ Nicaragua
◯ Niger
◯ Nigeria
◯ Niue
◯ Norfolk Island
◯ North Macedonia
◯ Northern Mariana Islands
◯ Norway
◯ Oman
◯ Pakistan
◯ Palau
◯ Palestine, State of
◯ Panama
◯ Papua New Guinea
◯ Paraguay
◯ Peru
◯ Philippines
◯ Pitcairn
◯ Poland
◯ Portugal
◯ Puerto Rico
◯ Qatar
◯ Réunion
◯ Romania
◯ Russian Federation
◯ Rwanda
◯ Saint Barthélemy
◯ Saint Helena, Ascension and Tristan da Cunha
◯ Saint Kitts and Nevis
◯ Saint Lucia
◯ Saint Martin (French part)
◯ Saint Pierre and Miquelon
◯ Saint Vincent and the Grenadines
◯ Samoa
◯ San Marino
◯ Sao Tome and Principe
◯ Saudi Arabia
◯ Senegal
◯ Serbia
◯ Seychelles
◯ Sierra Leone
◯ Singapore
◯ Sint Maarten (Dutch part)
◯ Slovakia
◯ Slovenia
◯ Solomon Islands
◯ Somalia
◯ South Africa
◯ South Georgia and the South Sandwich Islands
◯ South Sudan
◯ Spain
◯ Sri Lanka

◯ Sudan
◯ Suriname
◯ Svalbard and Jan Mayen
◯ Sweden
◯ Switzerland
◯ Syrian Arab Republic
◯ Taiwan
◯ Tajikistan
◯ Tanzania, United Republic of
◯ Thailand
◯ Timor-Leste
◯ Togo
◯ Tokelau
◯ Tonga
◯ Trinidad and Tobago
◯ Tunisia
◯ Turkey
◯ Turkmenistan
◯ Turks and Caicos Islands
◯ Tuvalu
◯ Uganda
◯ Ukraine
◯ United Arab Emirates
◯ United Kingdom of Great Britain and Northern Ireland
◯ United States of America
◯ United States Minor Outlying Islands
◯ Uruguay
◯ Uzbekistan
◯ Vanuatu
◯ Venezuela (Bolivarian Republic of)
◯ Viet Nam
◯ Virgin Islands (British)
◯ Virgin Islands (U.S.)
◯ Wallis and Futuna
◯ Western Sahara
◯ Yemen
◯ Zambia
◯ Zimbabwe

(Start typing and the country will be suggested. If you only visited 2 countries, move to the next question.)

País 3 en el que estuvo

- ◯ Afganistán
- ◯ Islas Aland
- ◯ Albania
- ◯ Argelia
- ◯ Samoa Americana
- ◯ Andorra
- ◯ Angola
- ◯ Anguila
- ◯ Antártida
- ◯ Antigua y Barbuda
- ◯ Argentina
- ◯ Armenia
- ◯ Aruba
- ◯ Australia
- ◯ Austria
- ◯ Azerbaiyán
- ◯ Bahamas
- ◯ Barein
- ◯ Bangladesh
- ◯ Barbados
- ◯ Bielorrusia
- ◯ Bélgica
- ◯ Belice
- ◯ Benin
- ◯ Islas Bermudas
- ◯ Bután
- ◯ Bolivia (Estado Plurinacional de)
- ◯ Bonaire, San Eustaquio y Saba
- ◯ Bosnia y Herzegovina
- ◯ Botsuana
- ◯ Isla Bouvet
- ◯ Brasil
- ◯ Territorio Británico del Océano Índico
- ◯ Brunei Darussalam
- ◯ Bulgaria
- ◯ Burkina Faso
- ◯ Burundi
- ◯ Cabo Verde
- ◯ Camboya
- ◯ Camerún
- ◯ Canadá
- ◯ Islas Caimán
- ◯ República Centroafricana
- ◯ Chad
- ◯ Chile
- ◯ China
- ◯ Isla de Navidad
- ◯ Islas Cocos (Keeling)
- ◯ Colombia
- ◯ Comoras
- ◯ Congo, República del
- ◯ Congo, República Democrática del
- ◯ Islas Cook
- ◯ Costa Rica
- ◯ Costa de Marfil
- ◯ Croacia
- ◯ Cuba
- ◯ Curazao
- ◯ Chipre
- ◯ Chequia
- ◯ Dinamarca
- ◯ Djibouti
- ◯ Dominica
- ◯ República Dominicana
- ◯ Ecuador
- ◯ Egipto
- ◯ El Salvador
- ◯ Guinea Ecuatorial
- ◯ Eritrea

◯ Estonia
◯ Eswatini
◯ Etiopía
◯ Islas Malvinas (Falkland Islands)
◯ Islas Faroe
◯ Fiyi
◯ Finlandia
◯ Francia
◯ Guayana Francesa
◯ Polinesia francesa
◯ Territorios Franceses del Sur
◯ Gabón
◯ Gambia
◯ Georgia
◯ Alemania
◯ Ghana
◯ Gibraltar
◯ Grecia
◯ Groenlandia
◯ Granada
◯ Guadalupe
◯ Guam
◯ Guatemala
◯ Guernsey
◯ Guinea
◯ Guinea-Bissau
◯ Guyana
◯ Haití
◯ Islas Heard y McDonald
◯ Santa Sede
◯ Honduras
◯ Hong Kong
◯ Hungría
◯ Islandia
◯ India
◯ Indonesia
◯ Irán (República Islámica de)
◯ Irak
◯ Irlanda
◯ Isla de Man
◯ Israel
◯ Italia
◯ Jamaica
◯ Japón
◯ Jersey
◯ Jordán
◯ Kazajstán
◯ Kenia
◯ Kiribati
◯ Corea (República Popular Democrática de)
◯ Corea, República de
◯ Kuwait
◯ Kirguistán
◯ República Democrática Popular Lao
◯ Letonia
◯ Líbano
◯ Lesoto
◯ Liberia
◯ Libia
◯ Liechtenstein
◯ Lituania
◯ Luxemburgo
◯ Macao
◯ Madagascar
◯ Malawi
◯ Malasia
◯ Maldivas
◯ Mali
◯ Malta
◯ Islas Marshall
◯ Martinica

◯ Mauritania
◯ Mauricio
◯ Mayotte
◯ México
◯ Micronesia (Estados Federados de)
◯ Moldavia, República de
◯ Mónaco
◯ Mongolia
◯ Montenegro
◯ Montserrat
◯ Marruecos
◯ Mozambique
◯ Myanmar
◯ Namibia
◯ Nauru
◯ Nepal
◯ Países Bajos
◯ Nueva Caledonia
◯ Nueva Zelanda
◯ Nicaragua
◯ Níger
◯ Nigeria
◯ Niue
◯ Isla Norfolk
◯ Macedonia del Norte
◯ Islas Marianas del Norte
◯ Noruega
◯ Omán
◯ Pakistán
◯ Palau
◯ Palestina, Estado de
◯ Panamá
◯ Papúa Nueva Guinea
◯ Paraguay
◯ Perú
◯ Filipinas
◯ Pitcairn
◯ Polonia
◯ Portugal
◯ Puerto Rico
◯ Katar
◯ Reunión
◯ Rumania
◯ Federación Rusa
◯ Ruanda
◯ San Bartolomé
◯ Santa Helena, Ascensión y Tristan da Cunha
◯ San Cristóbal y Nieves
◯ Santa Lucía
◯ San Martín (parte francesa)
◯ San Pedro y Miquelón
◯ San Vicente y las Granadinas
◯ Samoa
◯ San Marino
◯ Santo Tomé y Príncipe
◯ Arabia Saudita
◯ Senegal
◯ Serbia
◯ Seychelles
◯ Sierra Leona
◯ Singapur
◯ San Martín (parte holandesa)
◯ Eslovaquia
◯ Eslovenia
◯ Islas Salomón
◯ Somalia
◯ Sudáfrica
◯ Georgia del Sur y las Islas Sandwich del Sur
◯ Sudán del Sur
◯ España
◯ Sri Lanka

◯ Sudán
◯ Surinam
◯ Svalbard y Jan Mayen
◯ Suecia
◯ Suiza
◯ República Árabe Siria
◯ Taiwán
◯ Tayikistán
◯ Tanzania, República Unida de
◯ Tailandia
◯ Timor-Leste
◯ Togo
◯ Tokelau
◯ Tonga
◯ Trinidad y Tobago
◯ Túnez
◯ Turquía
◯ Turkmenistán
◯ Islas Turcas y Caicos
◯ Tuvalu
◯ Uganda
◯ Ucrania
◯ Emiratos Árabes Unidos
◯ Reino Unido de Gran Bretaña e Irlanda del Norte
◯ Estados Unidos de América
◯ Islas Ultramarinas Menores de los Estados Unidos
◯ Uruguay
◯ Uzbekistán
◯ Vanuatu
◯ Venezuela (República Bolivariana de)
◯ Vietnam
◯ Islas Vírgenes (británicas)
◯ Islas Vírgenes (EE. UU.)
◯ Wallis y Futuna
◯ Sahara Occidental
◯ Yemen
◯ Zambia
◯ Zimbabwe

(Comience a escribir y aparecerán sugerencias del país. Si solo fue a 2 países, pase a la siguiente pregunta.)

Country 4 visited

- ◯ Afghanistan
- ◯ Åland Islands
- ◯ Albania
- ◯ Algeria
- ◯ American Samoa
- ◯ Andorra
- ◯ Angola
- ◯ Anguilla
- ◯ Antarctica
- ◯ Antigua and Barbuda
- ◯ Argentina
- ◯ Armenia
- ◯ Aruba
- ◯ Australia
- ◯ Austria
- ◯ Azerbaijan
- ◯ Bahamas
- ◯ Bahrain
- ◯ Bangladesh
- ◯ Barbados
- ◯ Belarus
- ◯ Belgium
- ◯ Belize
- ◯ Benin
- ◯ Bermuda
- ◯ Bhutan
- ◯ Bolivia (Plurinational State of)
- ◯ Bonaire, Sint Eustatius and Saba
- ◯ Bosnia and Herzegovina
- ◯ Botswana
- ◯ Bouvet Island
- ◯ Brazil
- ◯ British Indian Ocean Territory
- ◯ Brunei Darussalam
- ◯ Bulgaria
- ◯ Burkina Faso
- ◯ Burundi
- ◯ Cabo Verde
- ◯ Cambodia
- ◯ Cameroon
- ◯ Canada
- ◯ Cayman Islands
- ◯ Central African Republic
- ◯ Chad
- ◯ Chile
- ◯ China
- ◯ Christmas Island
- ◯ Cocos (Keeling) Islands
- ◯ Colombia
- ◯ Comoros
- ◯ Congo, Republic of the
- ◯ Congo, Democratic Republic of the
- ◯ Cook Islands
- ◯ Costa Rica
- ◯ Côte d'Ivoire
- ◯ Croatia
- ◯ Cuba
- ◯ Curaçao
- ◯ Cyprus
- ◯ Czechia
- ◯ Denmark
- ◯ Djibouti
- ◯ Dominica
- ◯ Dominican Republic
- ◯ Ecuador
- ◯ Egypt
- ◯ El Salvador
- ◯ Equatorial Guinea
- ◯ Eritrea

◯ Estonia
◯ Eswatini
◯ Ethiopia
◯ Falkland Islands (Malvinas)
◯ Faroe Islands
◯ Fiji
◯ Finland
◯ France
◯ French Guiana
◯ French Polynesia
◯ French Southern Territories
◯ Gabon
◯ Gambia
◯ Georgia
◯ Germany
◯ Ghana
◯ Gibraltar
◯ Greece
◯ Greenland
◯ Grenada
◯ Guadeloupe
◯ Guam
◯ Guatemala
◯ Guernsey
◯ Guinea
◯ Guinea-Bissau
◯ Guyana
◯ Haiti
◯ Heard Island and McDonald Islands
◯ Holy See
◯ Honduras
◯ Hong Kong
◯ Hungary
◯ Iceland
◯ India
◯ Indonesia
◯ Iran (Islamic Republic of)
◯ Iraq
◯ Ireland
◯ Isle of Man
◯ Israel
◯ Italy
◯ Jamaica
◯ Japan
◯ Jersey
◯ Jordan
◯ Kazakhstan
◯ Kenya
◯ Kiribati
◯ Korea (Democratic People's Republic of)
◯ Korea, Republic of
◯ Kuwait
◯ Kyrgyzstan
◯ Lao People's Democratic Republic
◯ Latvia
◯ Lebanon
◯ Lesotho
◯ Liberia
◯ Libya
◯ Liechtenstein
◯ Lithuania
◯ Luxembourg
◯ Macao
◯ Madagascar
◯ Malawi
◯ Malaysia
◯ Maldives
◯ Mali
◯ Malta
◯ Marshall Islands
◯ Martinique

◯ Mauritania
◯ Mauritius
◯ Mayotte
◯ Mexico
◯ Micronesia (Federated States of)
◯ Moldova, Republic of
◯ Monaco
◯ Mongolia
◯ Montenegro
◯ Montserrat
◯ Morocco
◯ Mozambique
◯ Myanmar
◯ Namibia
◯ Nauru
◯ Nepal
◯ Netherlands
◯ New Caledonia
◯ New Zealand
◯ Nicaragua
◯ Niger
◯ Nigeria
◯ Niue
◯ Norfolk Island
◯ North Macedonia
◯ Northern Mariana Islands
◯ Norway
◯ Oman
◯ Pakistan
◯ Palau
◯ Palestine, State of
◯ Panama
◯ Papua New Guinea
◯ Paraguay
◯ Peru
◯ Philippines
◯ Pitcairn
◯ Poland
◯ Portugal
◯ Puerto Rico
◯ Qatar
◯ Réunion
◯ Romania
◯ Russian Federation
◯ Rwanda
◯ Saint Barthélemy
◯ Saint Helena, Ascension and Tristan da Cunha
◯ Saint Kitts and Nevis
◯ Saint Lucia
◯ Saint Martin (French part)
◯ Saint Pierre and Miquelon
◯ Saint Vincent and the Grenadines
◯ Samoa
◯ San Marino
◯ Sao Tome and Principe
◯ Saudi Arabia
◯ Senegal
◯ Serbia
◯ Seychelles
◯ Sierra Leone
◯ Singapore
◯ Sint Maarten (Dutch part)
◯ Slovakia
◯ Slovenia
◯ Solomon Islands
◯ Somalia
◯ South Africa
◯ South Georgia and the South Sandwich Islands
◯ South Sudan
◯ Spain
◯ Sri Lanka

◯ Sudan
◯ Suriname
◯ Svalbard and Jan Mayen
◯ Sweden
◯ Switzerland
◯ Syrian Arab Republic
◯ Taiwan
◯ Tajikistan
◯ Tanzania, United Republic of
◯ Thailand
◯ Timor-Leste
◯ Togo
◯ Tokelau
◯ Tonga
◯ Trinidad and Tobago
◯ Tunisia
◯ Turkey
◯ Turkmenistan
◯ Turks and Caicos Islands
◯ Tuvalu
◯ Uganda
◯ Ukraine
◯ United Arab Emirates
◯ United Kingdom of Great Britain and Northern Ireland
◯ United States of America
◯ United States Minor Outlying Islands
◯ Uruguay
◯ Uzbekistan
◯ Vanuatu
◯ Venezuela (Bolivarian Republic of)
◯ Viet Nam
◯ Virgin Islands (British)
◯ Virgin Islands (U.S.)
◯ Wallis and Futuna
◯ Western Sahara
◯ Yemen
◯ Zambia
◯ Zimbabwe

(Start typing and the country will be suggested. If you only visited 3 countries, move to the next question.)

País 4 en el que estuvo

◯ Afganistán
◯ Islas Aland
◯ Albania
◯ Argelia
◯ Samoa Americana
◯ Andorra
◯ Angola
◯ Anguila
◯ Antártida
◯ Antigua y Barbuda
◯ Argentina
◯ Armenia
◯ Aruba
◯ Australia
◯ Austria
◯ Azerbaiyán
◯ Bahamas
◯ Barein
◯ Bangladesh
◯ Barbados
◯ Bielorrusia
◯ Bélgica
◯ Belice
◯ Benin
◯ Islas Bermudas
◯ Bután
◯ Bolivia (Estado Plurinacional de)
◯ Bonaire, San Eustaquio y Saba
◯ Bosnia y Herzegovina
◯ Botsuana
◯ Isla Bouvet
◯ Brasil
◯ Territorio Británico del Océano Índico
◯ Brunei Darussalam
◯ Bulgaria
◯ Burkina Faso
◯ Burundi
◯ Cabo Verde
◯ Camboya
◯ Camerún
◯ Canadá
◯ Islas Caimán
◯ República Centroafricana
◯ Chad
◯ Chile
◯ China
◯ Isla de Navidad
◯ Islas Cocos (Keeling)
◯ Colombia
◯ Comoras
◯ Congo, República del
◯ Congo, República Democrática del
◯ Islas Cook
◯ Costa Rica
◯ Costa de Marfil
◯ Croacia
◯ Cuba
◯ Curazao
◯ Chipre
◯ Chequia
◯ Dinamarca
◯ Djibouti
◯ Dominica
◯ República Dominicana
◯ Ecuador
◯ Egipto
◯ El Salvador
◯ Guinea Ecuatorial
◯ Eritrea

◯ Estonia
◯ Eswatini
◯ Etiopía
◯ Islas Malvinas (Falkland Islands)
◯ Islas Faroe
◯ Fiyi
◯ Finlandia
◯ Francia
◯ Guayana Francesa
◯ Polinesia francesa
◯ Territorios Franceses del Sur
◯ Gabón
◯ Gambia
◯ Georgia
◯ Alemania
◯ Ghana
◯ Gibraltar
◯ Grecia
◯ Groenlandia
◯ Granada
◯ Guadalupe
◯ Guam
◯ Guatemala
◯ Guernsey
◯ Guinea
◯ Guinea-Bissau
◯ Guyana
◯ Haití
◯ Islas Heard y McDonald
◯ Santa Sede
◯ Honduras
◯ Hong Kong
◯ Hungría
◯ Islandia
◯ India
◯ Indonesia
◯ Irán (República Islámica de)
◯ Irak
◯ Irlanda
◯ Isla de Man
◯ Israel
◯ Italia
◯ Jamaica
◯ Japón
◯ Jersey
◯ Jordán
◯ Kazajstán
◯ Kenia
◯ Kiribati
◯ Corea (República Popular Democrática de)
◯ Corea, República de
◯ Kuwait
◯ Kirguistán
◯ República Democrática Popular Lao
◯ Letonia
◯ Líbano
◯ Lesoto
◯ Liberia
◯ Libia
◯ Liechtenstein
◯ Lituania
◯ Luxemburgo
◯ Macao
◯ Madagascar
◯ Malawi
◯ Malasia
◯ Maldivas
◯ Mali
◯ Malta
◯ Islas Marshall
◯ Martinica

◯ Mauritania
◯ Mauricio
◯ Mayotte
◯ México
◯ Micronesia (Estados Federados de)
◯ Moldavia, República de
◯ Mónaco
◯ Mongolia
◯ Montenegro
◯ Montserrat
◯ Marruecos
◯ Mozambique
◯ Myanmar
◯ Namibia
◯ Nauru
◯ Nepal
◯ Países Bajos
◯ Nueva Caledonia
◯ Nueva Zelanda
◯ Nicaragua
◯ Níger
◯ Nigeria
◯ Niue
◯ Isla Norfolk
◯ Macedonia del Norte
◯ Islas Marianas del Norte
◯ Noruega
◯ Omán
◯ Pakistán
◯ Palau
◯ Palestina, Estado de
◯ Panamá
◯ Papúa Nueva Guinea
◯ Paraguay
◯ Perú
◯ Filipinas
◯ Pitcairn
◯ Polonia
◯ Portugal
◯ Puerto Rico
◯ Katar
◯ Reunión
◯ Rumania
◯ Federación Rusa
◯ Ruanda
◯ San Bartolomé
◯ Santa Helena, Ascensión y Tristan da Cunha
◯ San Cristóbal y Nieves
◯ Santa Lucía
◯ San Martín (parte francesa)
◯ San Pedro y Miquelón
◯ San Vicente y las Granadinas
◯ Samoa
◯ San Marino
◯ Santo Tomé y Príncipe
◯ Arabia Saudita
◯ Senegal
◯ Serbia
◯ Seychelles
◯ Sierra Leona
◯ Singapur
◯ San Martín (parte holandesa)
◯ Eslovaquia
◯ Eslovenia
◯ Islas Salomón
◯ Somalia
◯ Sudáfrica
◯ Georgia del Sur y las Islas Sandwich del Sur
◯ Sudán del Sur
◯ España
◯ Sri Lanka

◯ Sudán
◯ Surinam
◯ Svalbard y Jan Mayen
◯ Suecia
◯ Suiza
◯ República Árabe Siria
◯ Taiwán
◯ Tayikistán
◯ Tanzania, República Unida de
◯ Tailandia
◯ Timor-Leste
◯ Togo
◯ Tokelau
◯ Tonga
◯ Trinidad y Tobago
◯ Túnez
◯ Turquía
◯ Turkmenistán
◯ Islas Turcas y Caicos
◯ Tuvalu
◯ Uganda
◯ Ucrania
◯ Emiratos Árabes Unidos
◯ Reino Unido de Gran Bretaña e Irlanda del Norte
◯ Estados Unidos de América
◯ Islas Ultramarinas Menores de los Estados Unidos
◯ Uruguay
◯ Uzbekistán
◯ Vanuatu
◯ Venezuela (República Bolivariana de)
◯ Vietnam
◯ Islas Vírgenes (británicas)
◯ Islas Vírgenes (EE. UU.)
◯ Wallis y Futuna
◯ Sahara Occidental
◯ Yemen
◯ Zambia
◯ Zimbabwe

(Comience a escribir y aparecerán sugerencias del país. Si solo fue a 3 países, pase a la siguiente pregunta.)

Country 5 visited

◯ Afghanistan
◯ Åland Islands
◯ Albania
◯ Algeria
◯ American Samoa
◯ Andorra
◯ Angola
◯ Anguilla
◯ Antarctica
◯ Antigua and Barbuda
◯ Argentina
◯ Armenia
◯ Aruba
◯ Australia
◯ Austria
◯ Azerbaijan
◯ Bahamas
◯ Bahrain
◯ Bangladesh
◯ Barbados
◯ Belarus
◯ Belgium
◯ Belize
◯ Benin
◯ Bermuda
◯ Bhutan
◯ Bolivia (Plurinational State of)
◯ Bonaire, Sint Eustatius and Saba
◯ Bosnia and Herzegovina
◯ Botswana
◯ Bouvet Island
◯ Brazil
◯ British Indian Ocean Territory
◯ Brunei Darussalam
◯ Bulgaria
◯ Burkina Faso
◯ Burundi
◯ Cabo Verde
◯ Cambodia
◯ Cameroon
◯ Canada
◯ Cayman Islands
◯ Central African Republic
◯ Chad
◯ Chile
◯ China
◯ Christmas Island
◯ Cocos (Keeling) Islands
◯ Colombia
◯ Comoros
◯ Congo, Republic of the
◯ Congo, Democratic Republic of the
◯ Cook Islands
◯ Costa Rica
◯ Côte d'Ivoire
◯ Croatia
◯ Cuba
◯ Curaçao
◯ Cyprus
◯ Czechia
◯ Denmark
◯ Djibouti
◯ Dominica
◯ Dominican Republic
◯ Ecuador
◯ Egypt
◯ El Salvador
◯ Equatorial Guinea
◯ Eritrea

◯ Estonia
◯ Eswatini
◯ Ethiopia
◯ Falkland Islands (Malvinas)
◯ Faroe Islands
◯ Fiji
◯ Finland
◯ France
◯ French Guiana
◯ French Polynesia
◯ French Southern Territories
◯ Gabon
◯ Gambia
◯ Georgia
◯ Germany
◯ Ghana
◯ Gibraltar
◯ Greece
◯ Greenland
◯ Grenada
◯ Guadeloupe
◯ Guam
◯ Guatemala
◯ Guernsey
◯ Guinea
◯ Guinea-Bissau
◯ Guyana
◯ Haiti
◯ Heard Island and McDonald Islands
◯ Holy See
◯ Honduras
◯ Hong Kong
◯ Hungary
◯ Iceland
◯ India
◯ Indonesia
◯ Iran (Islamic Republic of)
◯ Iraq
◯ Ireland
◯ Isle of Man
◯ Israel
◯ Italy
◯ Jamaica
◯ Japan
◯ Jersey
◯ Jordan
◯ Kazakhstan
◯ Kenya
◯ Kiribati
◯ Korea (Democratic People's Republic of)
◯ Korea, Republic of
◯ Kuwait
◯ Kyrgyzstan
◯ Lao People's Democratic Republic
◯ Latvia
◯ Lebanon
◯ Lesotho
◯ Liberia
◯ Libya
◯ Liechtenstein
◯ Lithuania
◯ Luxembourg
◯ Macao
◯ Madagascar
◯ Malawi
◯ Malaysia
◯ Maldives
◯ Mali
◯ Malta
◯ Marshall Islands
◯ Martinique

◯ Mauritania
◯ Mauritius
◯ Mayotte
◯ Mexico
◯ Micronesia (Federated States of)
◯ Moldova, Republic of
◯ Monaco
◯ Mongolia
◯ Montenegro
◯ Montserrat
◯ Morocco
◯ Mozambique
◯ Myanmar
◯ Namibia
◯ Nauru
◯ Nepal
◯ Netherlands
◯ New Caledonia
◯ New Zealand
◯ Nicaragua
◯ Niger
◯ Nigeria
◯ Niue
◯ Norfolk Island
◯ North Macedonia
◯ Northern Mariana Islands
◯ Norway
◯ Oman
◯ Pakistan
◯ Palau
◯ Palestine, State of
◯ Panama
◯ Papua New Guinea
◯ Paraguay
◯ Peru
◯ Philippines
◯ Pitcairn
◯ Poland
◯ Portugal
◯ Puerto Rico
◯ Qatar
◯ Réunion
◯ Romania
◯ Russian Federation
◯ Rwanda
◯ Saint Barthélemy
◯ Saint Helena, Ascension and Tristan da Cunha
◯ Saint Kitts and Nevis
◯ Saint Lucia
◯ Saint Martin (French part)
◯ Saint Pierre and Miquelon
◯ Saint Vincent and the Grenadines
◯ Samoa
◯ San Marino
◯ Sao Tome and Principe
◯ Saudi Arabia
◯ Senegal
◯ Serbia
◯ Seychelles
◯ Sierra Leone
◯ Singapore
◯ Sint Maarten (Dutch part)
◯ Slovakia
◯ Slovenia
◯ Solomon Islands
◯ Somalia
◯ South Africa
◯ South Georgia and the South Sandwich Islands
◯ South Sudan
◯ Spain
◯ Sri Lanka

◯ Sudan
◯ Suriname
◯ Svalbard and Jan Mayen
◯ Sweden
◯ Switzerland
◯ Syrian Arab Republic
◯ Taiwan
◯ Tajikistan
◯ Tanzania, United Republic of
◯ Thailand
◯ Timor-Leste
◯ Togo
◯ Tokelau
◯ Tonga
◯ Trinidad and Tobago
◯ Tunisia
◯ Turkey
◯ Turkmenistan
◯ Turks and Caicos Islands
◯ Tuvalu
◯ Uganda
◯ Ukraine
◯ United Arab Emirates
◯ United Kingdom of Great Britain and Northern Ireland
◯ United States of America
◯ United States Minor Outlying Islands
◯ Uruguay
◯ Uzbekistan
◯ Vanuatu
◯ Venezuela (Bolivarian Republic of)
◯ Viet Nam
◯ Virgin Islands (British)
◯ Virgin Islands (U.S.)
◯ Wallis and Futuna
◯ Western Sahara
◯ Yemen
◯ Zambia
◯ Zimbabwe

(Start typing and the country will be suggested. If you only visited 4 countries, move to the next question.)

País 5 en el que estuvo

◯ Afganistán
◯ Islas Aland
◯ Albania
◯ Argelia
◯ Samoa Americana
◯ Andorra
◯ Angola
◯ Anguila
◯ Antártida
◯ Antigua y Barbuda
◯ Argentina
◯ Armenia
◯ Aruba
◯ Australia
◯ Austria
◯ Azerbaiyán
◯ Bahamas
◯ Barein
◯ Bangladesh
◯ Barbados
◯ Bielorrusia
◯ Bélgica
◯ Belice
◯ Benin
◯ Islas Bermudas
◯ Bután
◯ Bolivia (Estado Plurinacional de)
◯ Bonaire, San Eustaquio y Saba
◯ Bosnia y Herzegovina
◯ Botsuana
◯ Isla Bouvet
◯ Brasil
◯ Territorio Británico del Océano Índico
◯ Brunei Darussalam
◯ Bulgaria
◯ Burkina Faso
◯ Burundi
◯ Cabo Verde
◯ Camboya
◯ Camerún
◯ Canadá
◯ Islas Caimán
◯ República Centroafricana
◯ Chad
◯ Chile
◯ China
◯ Isla de Navidad
◯ Islas Cocos (Keeling)
◯ Colombia
◯ Comoras
◯ Congo, República del
◯ Congo, República Democrática del
◯ Islas Cook
◯ Costa Rica
◯ Costa de Marfil
◯ Croacia
◯ Cuba
◯ Curazao
◯ Chipre
◯ Chequia
◯ Dinamarca
◯ Djibouti
◯ Dominica
◯ República Dominicana
◯ Ecuador
◯ Egipto
◯ El Salvador
◯ Guinea Ecuatorial
◯ Eritrea

◯ Estonia
◯ Eswatini
◯ Etiopía
◯ Islas Malvinas (Falkland Islands)
◯ Islas Faroe
◯ Fiyi
◯ Finlandia
◯ Francia
◯ Guayana Francesa
◯ Polinesia francesa
◯ Territorios Franceses del Sur
◯ Gabón
◯ Gambia
◯ Georgia
◯ Alemania
◯ Ghana
◯ Gibraltar
◯ Grecia
◯ Groenlandia
◯ Granada
◯ Guadalupe
◯ Guam
◯ Guatemala
◯ Guernsey
◯ Guinea
◯ Guinea-Bissau
◯ Guyana
◯ Haití
◯ Islas Heard y McDonald
◯ Santa Sede
◯ Honduras
◯ Hong Kong
◯ Hungría
◯ Islandia
◯ India
◯ Indonesia
◯ Irán (República Islámica de)
◯ Irak
◯ Irlanda
◯ Isla de Man
◯ Israel
◯ Italia
◯ Jamaica
◯ Japón
◯ Jersey
◯ Jordán
◯ Kazajstán
◯ Kenia
◯ Kiribati
◯ Corea (República Popular Democrática de)
◯ Corea, República de
◯ Kuwait
◯ Kirguistán
◯ República Democrática Popular Lao
◯ Letonia
◯ Líbano
◯ Lesoto
◯ Liberia
◯ Libia
◯ Liechtenstein
◯ Lituania
◯ Luxemburgo
◯ Macao
◯ Madagascar
◯ Malawi
◯ Malasia
◯ Maldivas
◯ Mali
◯ Malta
◯ Islas Marshall
◯ Martinica

◯ Mauritania
◯ Mauricio
◯ Mayotte
◯ México
◯ Micronesia (Estados Federados de)
◯ Moldavia, República de
◯ Mónaco
◯ Mongolia
◯ Montenegro
◯ Montserrat
◯ Marruecos
◯ Mozambique
◯ Myanmar
◯ Namibia
◯ Nauru
◯ Nepal
◯ Países Bajos
◯ Nueva Caledonia
◯ Nueva Zelanda
◯ Nicaragua
◯ Níger
◯ Nigeria
◯ Niue
◯ Isla Norfolk
◯ Macedonia del Norte
◯ Islas Marianas del Norte
◯ Noruega
◯ Omán
◯ Pakistán
◯ Palau
◯ Palestina, Estado de
◯ Panamá
◯ Papúa Nueva Guinea
◯ Paraguay
◯ Perú
◯ Filipinas
◯ Pitcairn
◯ Polonia
◯ Portugal
◯ Puerto Rico
◯ Katar
◯ Reunión
◯ Rumania
◯ Federación Rusa
◯ Ruanda
◯ San Bartolomé
◯ Santa Helena, Ascensión y Tristan da Cunha
◯ San Cristóbal y Nieves
◯ Santa Lucía
◯ San Martín (parte francesa)
◯ San Pedro y Miquelón
◯ San Vicente y las Granadinas
◯ Samoa
◯ San Marino
◯ Santo Tomé y Príncipe
◯ Arabia Saudita
◯ Senegal
◯ Serbia
◯ Seychelles
◯ Sierra Leona
◯ Singapur
◯ San Martín (parte holandesa)
◯ Eslovaquia
◯ Eslovenia
◯ Islas Salomón
◯ Somalia
◯ Sudáfrica
◯ Georgia del Sur y las Islas Sandwich del Sur
◯ Sudán del Sur
◯ España
◯ Sri Lanka

	◯ Sudán<br>◯ Surinam<br>◯ Svalbard y Jan Mayen<br>◯ Suecia<br>◯ Suiza<br>◯ República Árabe Siria<br>◯ Taiwán<br>◯ Tayikistán<br>◯ Tanzania, República Unida de<br>◯ Tailandia<br>◯ Timor-Leste<br>◯ Togo<br>◯ Tokelau<br>◯ Tonga<br>◯ Trinidad y Tobago<br>◯ Túnez<br>◯ Turquía<br>◯ Turkmenistán<br>◯ Islas Turcas y Caicos<br>◯ Tuvalu<br>◯ Uganda<br>◯ Ucrania<br>◯ Emiratos Árabes Unidos<br>◯ Reino Unido de Gran Bretaña e Irlanda del Norte<br>◯ Estados Unidos de América<br>◯ Islas Ultramarinas Menores de los Estados Unidos<br>◯ Uruguay<br>◯ Uzbekistán<br>◯ Vanuatu<br>◯ Venezuela (República Bolivariana de)<br>◯ Vietnam<br>◯ Islas Vírgenes (británicas)<br>◯ Islas Vírgenes (EE. UU.)<br>◯ Wallis y Futuna<br>◯ Sahara Occidental<br>◯ Yemen<br>◯ Zambia<br>◯ Zimbabwe<br>(Comience a escribir y aparecerán sugerencias del país. Si solo fue a 4 países, pase a la siguiente pregunta.)
In the past 7 days, have you visited a state other than Washington?	◯ Yes<br>◯ No - I have not traveled to a state outside Washington
En los últimos 7 días, ¿ha estado en un estado aparte de Washington?	◯ Sí<br>◯ No - No he viajado fuera del estado de Washington

State 1

◯ Alabama
◯ Alaska
◯ Arizona
◯ Arkansas
◯ California
◯ Colorado
◯ Connecticut
◯ Delaware
◯ District of Columbia
◯ Florida
◯ Georgia
◯ Hawaii
◯ Idaho
◯ Illinois
◯ Indiana
◯ Iowa
◯ Kansas
◯ Kentucky
◯ Louisiana
◯ Maine
◯ Maryland
◯ Massachusetts
◯ Michigan
◯ Minnesota
◯ Mississippi
◯ Missouri
◯ Montana
◯ Nebraska
◯ Nevada
◯ New Hampshire
◯ New Jersey
◯ New Mexico
◯ New York
◯ North Carolina
◯ North Dakota
◯ Ohio
◯ Oklahoma
◯ Oregon
◯ Pennsylvania
◯ Rhode Island
◯ South Carolina
◯ South Dakota
◯ Tennessee
◯ Texas
◯ Utah
◯ Vermont
◯ Virginia
◯ Washington
◯ West Virginia
◯ Wisconsin
◯ Wyoming
◯ American Samoa
◯ Guam
◯ Northern Mariana Islands
◯ Puerto Rico
◯ U.S. Minor Outlying Islands
◯ U.S. Virgin Islands
(Start typing and the state will be suggested.)

Estado 1

◯ Alabama
◯ Alaska
◯ Arizona
◯ Arkansas
◯ California
◯ Colorado
◯ Connecticut
◯ Delaware
◯ Distrito de Columbia
◯ Florida
◯ Georgia
◯ Hawaii
◯ Idaho
◯ Illinois
◯ Indiana
◯ Iowa
◯ Kansas
◯ Kentucky
◯ Luisiana
◯ Maine
◯ MD Maryland
◯ Massachusetts
◯ Michigan
◯ Minnesota
◯ Mississippi
◯ Missouri
◯ Montana
◯ Nebraska
◯ Nevada
◯ New Hampshire
◯ New Jersey
◯ New Mexico
◯ New York
◯ Carolina del Norte
◯ Dakota del Norte
◯ Ohio
◯ Oklahoma
◯ Oregon
◯ Pensilvania
◯ Rhode Island
◯ Carolina del Sur
◯ Dakota del Sur
◯ Tennessee
◯ Texas
◯ Utah
◯ Vermont
◯ Virginia
◯ Washington
◯ Virginia Occidental
◯ Wisconsin
◯ Wyoming
◯ Samoa Americana
◯ Guam
◯ Islas Marianas del Norte
◯ Puerto Rico
◯ Islas Ultramarinas Menores de EE. UU.
◯ Islas Vírgenes de EE.UU
◯ Ninguna de las anteriores respuestas
(Comience a escribir y aparecerán sugerencias del estado.)

State 2

◯ Alabama
◯ Alaska
◯ Arizona
◯ Arkansas
◯ California
◯ Colorado
◯ Connecticut
◯ Delaware
◯ District of Columbia
◯ Florida
◯ Georgia
◯ Hawaii
◯ Idaho
◯ Illinois
◯ Indiana
◯ Iowa
◯ Kansas
◯ Kentucky
◯ Louisiana
◯ Maine
◯ Maryland
◯ Massachusetts
◯ Michigan
◯ Minnesota
◯ Mississippi
◯ Missouri
◯ Montana
◯ Nebraska
◯ Nevada
◯ New Hampshire
◯ New Jersey
◯ New Mexico
◯ New York
◯ North Carolina
◯ North Dakota
◯ Ohio
◯ Oklahoma
◯ Oregon
◯ Pennsylvania
◯ Rhode Island
◯ South Carolina
◯ South Dakota
◯ Tennessee
◯ Texas
◯ Utah
◯ Vermont
◯ Virginia
◯ Washington
◯ West Virginia
◯ Wisconsin
◯ Wyoming
◯ American Samoa
◯ Guam
◯ Northern Mariana Islands
◯ Puerto Rico
◯ U.S. Minor Outlying Islands
◯ U.S. Virgin Islands

(Start typing and the state will be suggested. If you only visited 1 state, move to the next question.)

Estado 2

◯ Alabama
◯ Alaska
◯ Arizona
◯ Arkansas
◯ California
◯ Colorado
◯ Connecticut
◯ Delaware
◯ Distrito de Columbia
◯ Florida
◯ Georgia
◯ Hawaii
◯ Idaho
◯ Illinois
◯ Indiana
◯ Iowa
◯ Kansas
◯ Kentucky
◯ Luisiana
◯ Maine
◯ MD Maryland
◯ Massachusetts
◯ Michigan
◯ Minnesota
◯ Mississippi
◯ Missouri
◯ Montana
◯ Nebraska
◯ Nevada
◯ New Hampshire
◯ New Jersey
◯ New Mexico
◯ New York
◯ Carolina del Norte
◯ Dakota del Norte
◯ Ohio
◯ Oklahoma
◯ Oregon
◯ Pensilvania
◯ Rhode Island
◯ Carolina del Sur
◯ Dakota del Sur
◯ Tennessee
◯ Texas
◯ Utah
◯ Vermont
◯ Virginia
◯ Washington
◯ Virginia Occidental
◯ Wisconsin
◯ Wyoming
◯ Samoa Americana
◯ Guam
◯ Islas Marianas del Norte
◯ Puerto Rico
◯ Islas Ultramarinas Menores de EE. UU.
◯ Islas Vírgenes de EE.UU
◯ Ninguna de las anteriores respuestas
(Comience a escribir y aparecerán sugerencias del estado. Si solo fue a 1 estado, pase a la siguiente pregunta.)

State 3

◯ Alabama
◯ Alaska
◯ Arizona
◯ Arkansas
◯ California
◯ Colorado
◯ Connecticut
◯ Delaware
◯ District of Columbia
◯ Florida
◯ Georgia
◯ Hawaii
◯ Idaho
◯ Illinois
◯ Indiana
◯ Iowa
◯ Kansas
◯ Kentucky
◯ Louisiana
◯ Maine
◯ Maryland
◯ Massachusetts
◯ Michigan
◯ Minnesota
◯ Mississippi
◯ Missouri
◯ Montana
◯ Nebraska
◯ Nevada
◯ New Hampshire
◯ New Jersey
◯ New Mexico
◯ New York
◯ North Carolina
◯ North Dakota
◯ Ohio
◯ Oklahoma
◯ Oregon
◯ Pennsylvania
◯ Rhode Island
◯ South Carolina
◯ South Dakota
◯ Tennessee
◯ Texas
◯ Utah
◯ Vermont
◯ Virginia
◯ Washington
◯ West Virginia
◯ Wisconsin
◯ Wyoming
◯ American Samoa
◯ Guam
◯ Northern Mariana Islands
◯ Puerto Rico
◯ U.S. Minor Outlying Islands
◯ U.S. Virgin Islands

(Start typing and the state will be suggested. If you only visited 2 states, move to the next question.)

Estado 3

◯ Alabama
◯ Alaska
◯ Arizona
◯ Arkansas
◯ California
◯ Colorado
◯ Connecticut
◯ Delaware
◯ Distrito de Columbia
◯ Florida
◯ Georgia
◯ Hawaii
◯ Idaho
◯ Illinois
◯ Indiana
◯ Iowa
◯ Kansas
◯ Kentucky
◯ Luisiana
◯ Maine
◯ MD Maryland
◯ Massachusetts
◯ Michigan
◯ Minnesota
◯ Mississippi
◯ Missouri
◯ Montana
◯ Nebraska
◯ Nevada
◯ New Hampshire
◯ New Jersey
◯ New Mexico
◯ New York
◯ Carolina del Norte
◯ Dakota del Norte
◯ Ohio
◯ Oklahoma
◯ Oregon
◯ Pensilvania
◯ Rhode Island
◯ Carolina del Sur
◯ Dakota del Sur
◯ Tennessee
◯ Texas
◯ Utah
◯ Vermont
◯ Virginia
◯ Washington
◯ Virginia Occidental
◯ Wisconsin
◯ Wyoming
◯ Samoa Americana
◯ Guam
◯ Islas Marianas del Norte
◯ Puerto Rico
◯ Islas Ultramarinas Menores de EE. UU.
◯ Islas Vírgenes de EE.UU
◯ Ninguna de las anteriores respuestas

(Comience a escribir y aparecerán sugerencias del estado. Si solo fue a 2 estados, pase a la siguiente pregunta.)

State 4

◯ Alabama
◯ Alaska
◯ Arizona
◯ Arkansas
◯ California
◯ Colorado
◯ Connecticut
◯ Delaware
◯ District of Columbia
◯ Florida
◯ Georgia
◯ Hawaii
◯ Idaho
◯ Illinois
◯ Indiana
◯ Iowa
◯ Kansas
◯ Kentucky
◯ Louisiana
◯ Maine
◯ Maryland
◯ Massachusetts
◯ Michigan
◯ Minnesota
◯ Mississippi
◯ Missouri
◯ Montana
◯ Nebraska
◯ Nevada
◯ New Hampshire
◯ New Jersey
◯ New Mexico
◯ New York
◯ North Carolina
◯ North Dakota
◯ Ohio
◯ Oklahoma
◯ Oregon
◯ Pennsylvania
◯ Rhode Island
◯ South Carolina
◯ South Dakota
◯ Tennessee
◯ Texas
◯ Utah
◯ Vermont
◯ Virginia
◯ Washington
◯ West Virginia
◯ Wisconsin
◯ Wyoming
◯ American Samoa
◯ Guam
◯ Northern Mariana Islands
◯ Puerto Rico
◯ U.S. Minor Outlying Islands
◯ U.S. Virgin Islands

(Start typing and the state will be suggested. If you only visited 3 states, move to the next question.)

Estado 4

◯ Alabama
◯ Alaska
◯ Arizona
◯ Arkansas
◯ California
◯ Colorado
◯ Connecticut
◯ Delaware
◯ Distrito de Columbia
◯ Florida
◯ Georgia
◯ Hawaii
◯ Idaho
◯ Illinois
◯ Indiana
◯ Iowa
◯ Kansas
◯ Kentucky
◯ Luisiana
◯ Maine
◯ MD Maryland
◯ Massachusetts
◯ Michigan
◯ Minnesota
◯ Mississippi
◯ Missouri
◯ Montana
◯ Nebraska
◯ Nevada
◯ New Hampshire
◯ New Jersey
◯ New Mexico
◯ New York
◯ Carolina del Norte
◯ Dakota del Norte
◯ Ohio
◯ Oklahoma
◯ Oregon
◯ Pensilvania
◯ Rhode Island
◯ Carolina del Sur
◯ Dakota del Sur
◯ Tennessee
◯ Texas
◯ Utah
◯ Vermont
◯ Virginia
◯ Washington
◯ Virginia Occidental
◯ Wisconsin
◯ Wyoming
◯ Samoa Americana
◯ Guam
◯ Islas Marianas del Norte
◯ Puerto Rico
◯ Islas Ultramarinas Menores de EE. UU.
◯ Islas Vírgenes de EE.UU
◯ Ninguna de las anteriores respuestas

(Comience a escribir y aparecerán sugerencias del estado. Si solo fue a 3 estados, pase a la siguiente pregunta.)

State 5

◯ Alabama
◯ Alaska
◯ Arizona
◯ Arkansas
◯ California
◯ Colorado
◯ Connecticut
◯ Delaware
◯ District of Columbia
◯ Florida
◯ Georgia
◯ Hawaii
◯ Idaho
◯ Illinois
◯ Indiana
◯ Iowa
◯ Kansas
◯ Kentucky
◯ Louisiana
◯ Maine
◯ Maryland
◯ Massachusetts
◯ Michigan
◯ Minnesota
◯ Mississippi
◯ Missouri
◯ Montana
◯ Nebraska
◯ Nevada
◯ New Hampshire
◯ New Jersey
◯ New Mexico
◯ New York
◯ North Carolina
◯ North Dakota
◯ Ohio
◯ Oklahoma
◯ Oregon
◯ Pennsylvania
◯ Rhode Island
◯ South Carolina
◯ South Dakota
◯ Tennessee
◯ Texas
◯ Utah
◯ Vermont
◯ Virginia
◯ Washington
◯ West Virginia
◯ Wisconsin
◯ Wyoming
◯ American Samoa
◯ Guam
◯ Northern Mariana Islands
◯ Puerto Rico
◯ U.S. Minor Outlying Islands
◯ U.S. Virgin Islands

(Start typing and the state will be suggested. If you only visited 4 states, move to the next question.)

Estado 5

◯ Alabama
◯ Alaska
◯ Arizona
◯ Arkansas
◯ California
◯ Colorado
◯ Connecticut
◯ Delaware
◯ Distrito de Columbia
◯ Florida
◯ Georgia
◯ Hawaii
◯ Idaho
◯ Illinois
◯ Indiana
◯ Iowa
◯ Kansas
◯ Kentucky
◯ Luisiana
◯ Maine
◯ MD Maryland
◯ Massachusetts
◯ Michigan
◯ Minnesota
◯ Mississippi
◯ Missouri
◯ Montana
◯ Nebraska
◯ Nevada
◯ New Hampshire
◯ New Jersey
◯ New Mexico
◯ New York
◯ Carolina del Norte
◯ Dakota del Norte
◯ Ohio
◯ Oklahoma
◯ Oregon
◯ Pensilvania
◯ Rhode Island
◯ Carolina del Sur
◯ Dakota del Sur
◯ Tennessee
◯ Texas
◯ Utah
◯ Vermont
◯ Virginia
◯ Washington
◯ Virginia Occidental
◯ Wisconsin
◯ Wyoming
◯ Samoa Americana
◯ Guam
◯ Islas Marianas del Norte
◯ Puerto Rico
◯ Islas Ultramarinas Menores de EE. UU.
◯ Islas Vírgenes de EE.UU
◯ Ninguna de las anteriores respuestas

(Comience a escribir y aparecerán sugerencias del estado. Si solo fue a 4 estados, pase a la siguiente pregunta.)

What was the purpose of your recent travel? Select all that apply.	☐ Tourism<br>☐ Business<br>☐ Visiting family and/or friends<br>☐ I just moved here from another US state<br>☐ I just moved here from another country<br>☐ Other
¿Cuál fue el propósito de su viaje reciente? Seleccione todas las que correspondan.	☐ Turismo<br>☐ Negocios<br>☐ Visitar a familiares o amigos<br>☐ Me acabo de mudar aquí desde otro estado de los EE. UU.<br>☐ Me acabo de mudar aquí de otro país<br>☐ Otra
How or where did you hear about the Seattle Flu Study? Select all that apply.	☐ Saw a kiosk<br>☐ Saw an ad on Facebook or Instagram or Twitter<br>☐ Saw an ad on Google<br>☐ Email<br>☐ Other online source<br>☐ Heard about it from my healthcare provider<br>☐ Heard about it at a travel clinic or immigrant/refugee health screening<br>☐ Heard about it from a friend or family member<br>☐ Heard about it from my child's school<br>☐ Heard about it from my place of work<br>☐ Other<br>☐ I don't remember / Prefer not to say
¿Cómo o dónde se enteró del Estudio de la Gripe de Seattle? Seleccione todas las que correspondan.	☐ Lo vi en un quiosco<br>☐ Lo vi en un anuncio en Facebook o Instagram o Twitter<br>☐ Lo vi en un anuncio en Google<br>☐ Correo electrónico<br>☐ Otra fuente en línea<br>☐ Me lo dijo mi proveedor de atención médica<br>☐ Me enteré en una clínica para viajeros o en una evaluación de salud de inmigrantes/refugiados<br>☐ Me dijo un amigo o familiar<br>☐ Me enteré a través de la escuela de mi hijo<br>☐ Me enteré en mi trabajo<br>☐ Otra<br>☐ No recuerdo / Prefiero no decir

Thanks for completing the questionnaire. Please give the iPad back to a researcher, who will help you with the next steps.

Gracias por contestar el cuestionario. Devuélvale el iPad a un investigador que le ayudara con los proximos pasos.

Scan the yellow SFS sample barcode by clicking the "scan barcode" button. If the scanner isn't working, leave this field blank and enter the barcode manually.	__________________________________
Please enter the yellow sample barcode manually or rescan the barcode above. You cannot proceed until this has been entered.	__________________________________
Scan the red CLIA barcode by clicking the "scan barcode" button. If the scanner isn't working, leave this field blank and enter the barcode manually.	__________________________________

Please enter the red CLIA barcode manually or rescan the barcode above. You cannot proceed until this has been entered.	______________________
Place a blue dot on the tube to indicate that the sample is being tested with Cepheid.	
Specimen collection date	______________________
Gift Card Type	◯ Amazon<br>◯ Starbucks<br>◯ Target
Scan the [giftcard] giftcard barcode by clicking the "scan barcode" button. If the scanner isn't working, leave this field blank and enter the barcode manually.	______________________
Please manually enter the last 4 digits of the [giftcard] giftcard barcode/ID. You cannot proceed until this has been done.	______________________
Scan the Cepheid cartridge QR code by clicking the "scan barcode" button. If the scanner isn't working, leave this field blank and enter the barcode manually.	______________________
Please select the door that you are placing sample [sfs_barcode] [sfs_barcode_manual] in.	◯ Module A1<br>◯ Module A2<br>◯ Module A3<br>◯ Module A4
This is the questionnaire for: Name: [participant_first_name] [part_name_sp]<br><br>Barcode: [sfs_barcode] [sfs_barcode_manual]<br><br>Cepheid Door: [cepheid_door]<br><br>Please confirm that this information is correct before you enter the results.	◯ I confirm that this is the correct survey for the sample.
Cepheid Test Results - Select all that apply.	☐ Not positive for anything<br>☐ Influenza A +<br>☐ Influenza B +<br>☐ RSV +<br>☐ Inconclusive
Please confirm the following is correct. By selecting yes, you will automatically trigger an email to the participant with their results.<br><br>Participant Name: [participant_first_name] [part_name_sp]<br><br>Barcode: [sfs_barcode] [sfs_barcode_manual]<br><br>Cepheid Door: [cepheid_door]<br><br>Results: [cepheid_results:checked]	◯ Yes, this is correct.

FLAG: This participant is considered high-risk because they have one of the following conditions: __________________________________

Participant age: [age] years old
Participant pregnant? [pregnant_yesno]
Chronic illnesses: [chronic_illness:checked]
Living situation: [housing_type:checked]
Race: [race:checked]
