# Supplementary material for: “I probably shouldn’t go in today”: Inequitable access to paid sick leave and its impacts on health behaviors during the emergence of COVID-19 in the Seattle area
Source: PLoS One. 2024 Sep 10;19(9):e0307734. doi: 10.1371/journal.pone.0307734 (PMC11386467; doi:10.1371/journal.pone.0307734)
Supplement: S2 File — (ZIP) [file pone.0307734.s002.zip › Copy of AppendixC_SFS_Illness_Questionnaire.pdf]

Illness Questionnaire & Nasal Swab Collection

Hello [first_name_1]!

Please complete this two-part survey.

Part 1: Illness Questionnaire Part 2: Nasal Swab Collection

Part 1: Illness Questionnaire

How long ago did you first notice your current illness?
Select the date on the calendar.

Note: Today's date is highlighted in yellow on the calendar.

____
(MM-DD-YYYY)

Around when did your symptoms start?

- ◯ Half a day ago
- ◯ Half a day - 1 day ago
- ◯ 1 - 1.5 days ago
- ◯ 1.5 - 2 days ago
- ◯ 3 days ago
- ◯ 4 days ago
- ◯ 5 or more days ago

How long did it take you to go from feeling not sick at all to feeling the sickest you have felt?

- ◯ Half a day
- ◯ Half a day - 1 day
- ◯ 1 - 1.5 days
- ◯ 1.5 - 2 days
- ◯ 3 days
- ◯ 4 days
- ◯ 5 or more days

Which new or worsening symptoms have you experienced in the last 7 days? Select all that apply.

- ☐ Feeling feverish
- ☐ Headache
- ☐ Cough
- ☐ Chills or shivering
- ☐ Sweats
- ☐ Sore throat or itchy/scratchy throat
- ☐ Nausea or vomiting
- ☐ Runny or stuffy nose
- ☐ Feeling more tired than usual
- ☐ Muscle or body aches
- ☐ Increased trouble with breathing
- ☐ Diarrhea
- ☐ Rash
- ☐ Ear pain or ear discharge
- ☐ None of the above

How severe are your symptoms? (Select the level of discomfort you have felt at the worst point so far).

	Mild	Moderate	Severe
Feeling feverish	◯	◯	◯
Cough	◯	◯	◯
Muscle or body aches	◯	◯	◯
Feeling more tired than usual	◯	◯	◯
Sore throat or itchy/scratchy throat	◯	◯	◯

Demographics

What is the sex on your medical records?

◯ Male
◯ Female
◯ Indeterminate/other
◯ Prefer not to say

What is your sex?

◯ Male
◯ Female
◯ Other (please specify)
◯ Prefer not to say

What is your sex? Please specify.

____

Are you Hispanic or Latino?

◯ Yes
◯ No
◯ Prefer not to say

How would you describe your race? (Select all that apply)

☐ American Indian or Alaska Native
☐ Asian
☐ Native Hawaiian or other Pacific Islander
☐ Black or African American
☐ White
☐ Other
☐ Prefer not to say
(Select all that apply.)

What is the highest level of education you have completed?

◯ Less than high school graduate
◯ Graduated high school/obtained GED
◯ Some college (including vocational training, associate's degree)
◯ Bachelor's degree
◯ Advanced degree
◯ Prefer not to say

Please choose the range that best represents your household income last year (before taxes). If you are still considered a "dependent" for tax purposes, choose the range that describes your parent/legal guardian's household income.	◯ Less than or equal to $25,000<br>◯ Between $25 and 50 thousand ($25,001 to $50,000)<br>◯ Between $50 and 75 thousand ($50,001 to $75,000)<br>◯ Between $75 and 100 thousand ($75,001 to $100,000)<br>◯ Between $100 and 125 thousand ($100,001 to $125,000)<br>◯ Between $125 and 150 thousand ($125,001 to $150,000)<br>◯ Over $150,000<br>◯ Don't know<br>◯ Prefer not to say
What type of health insurance do you have?	☐ Private (provided by employer and/or purchased)<br>☐ Government (Medicare/Medicaid)<br>☐ Other<br>☐ None<br>☐ Prefer not to say<br>(Select all that apply.)
Are you currently pregnant?	◯ Yes<br>◯ No<br>◯ Prefer not to say

Next, we will ask you some questions about your living arrangement.

Are you affiliated with the University of Washington?	◯ Yes, I am an undergraduate student<br>◯ Yes, I am a graduate/professional student<br>◯ Yes, I am a faculty member<br>◯ Yes, I am a staff member/university employee<br>◯ No
What kind of residence do you live in?	◯ On-campus residence hall<br>◯ On-campus apartment<br>◯ Greek life housing<br>◯ Off-campus house<br>◯ Off-campus apartment<br>◯ Other
Where do you live?	◯ House/condo/townhouse<br>◯ Shelter<br>◯ Apartment<br>◯ Dormitory<br>◯ Assisted living facility<br>◯ Skilled nursing center<br>◯ No consistent primary residence<br>◯ Other
What is your usual nighttime accommodation?	◯ Shelter<br>◯ Transitional housing/safe haven<br>◯ Street/outside/tent/encampment<br>◯ Abandoned building/squat<br>◯ Vehicle (car, van, RV, camper)<br>◯ Hotel or motel<br>◯ Prefer not to say<br>◯ None of the above

Which shelter has served as your primary residence over the last 7 days?	◯ Aloha Inn<br>◯ Blaine Center Homeless Ministry<br>◯ Bread of Life Mission<br>◯ Compass Housing Alliance<br>◯ DESC (Downtown Emergency Service Center)<br>◯ Elizabeth Gregory House<br>◯ Hammond House Women's Shelter<br>◯ Jubilee Women's Center<br>◯ King County Men's Winter Shelter<br>◯ Mary's Place<br>◯ Noel House Women's Referral Center<br>◯ Pike Market Senior Center<br>◯ Roots Young Adult Shelter<br>◯ Sacred Heart Shelter<br>◯ Saint Martin de Porres Shelter<br>◯ Salvation Army Women's Shelter<br>◯ Seattle City Hall Shelter<br>◯ Seattle Union Gospel Mission for Men<br>◯ YMCA Emergency Shelter<br>◯ Other/none of the above
Including yourself, how many people share your kitchen or living space?	◯ I live by myself<br>◯ 2 people<br>◯ 3 people<br>◯ 4 people<br>◯ 5 people<br>◯ 6 or more people
Including yourself, how many people are in the room you sleep in?	◯ Just myself<br>◯ 2 people<br>◯ 3 people<br>◯ 4 people<br>◯ 5 people<br>◯ 6 or more people
What age groups of children are in your house?<br>Select all that apply.	☐ No children<br>☐ Age 0-5 years<br>☐ Age 6-12 years<br>☐ Age 13-18 years
What age groups of children stay in the room that you sleep in?<br>Select all that apply.	☐ No children<br>☐ Age 0-5 years<br>☐ Age 6-12 years<br>☐ Age 13-18 years
Do these children attend daycare or a child care center?	◯ Yes<br>◯ No
Approximately how many hours per week do these children attend daycare or childcare?	__________________________________

Your Health

In the past 7 days, has someone you live with been diagnosed with the flu by a medical professional?	◯ Yes<br>◯ No<br>◯ Do not know
Have you sought clinical care for your current illness since it started?	◯ No<br>◯ Yes; Doctor's office or Urgent Care<br>◯ Yes; Pharmacy (drugstore)<br>◯ Yes; Hospital or Emergency Department<br>◯ Yes; Other
Are you receiving an antiviral prescribed by a medical professional for the treatment or prevention of flu?	◯ No<br>◯ Yes; Oseltamivir (Tamiflu)<br>◯ Yes; Zanamivir (Relenza)<br>◯ Yes; Peramivir (Rapivab)<br>◯ Yes; Baloxavir (Xofluza)<br>◯ Yes, but I don't know which medication<br>◯ Do not know
Are you receiving an antibiotic prescribed by a medical professional for your illness?	◯ No<br>◯ Yes; Zithromycin (Z-pack or Zithromax)<br>◯ Yes; Amoxicillin (Moxatag)<br>◯ Yes; Amoxicillin/Clavulanate (Augmentin)<br>◯ Yes; Levofloxacin (Levaquin)<br>◯ Yes; Moxifloxacin (Avelox)<br>◯ Yes, but I don't remember which antibiotic<br>◯ Yes, but my antibiotic is not listed<br>◯ Do not know
At my place of work, employees are encouraged to take time off or work from home if they are sick.	◯ Yes, and I would be paid for hours missed ◯ Yes, but I would not be paid for hours missed ◯ No ◯ I am not currently employed
How has your current illness affected your ability to do your regular activities (work, school, etc.)?	◯ Not at all<br>◯ A little bit<br>◯ Somewhat<br>◯ Quite a bit<br>◯ Very much
Other than work and/or school, which of the following daily activities have been impacted by your current illness?	☐ Running errands<br>☐ Exercising<br>☐ Socializing<br>☐ Volunteering<br>☐ Ability to take care of myself and/or family<br>☐ None of the above/ my activities have not been impacted<br>(Select all that apply.)
Has your current illness kept you from doing any of the following?	☐ Attending class<br>☐ Going to work<br>☐ Studying<br>☐ Performing well on an exam or written assignment<br>☐ None of the above/ my activities have not been impacted<br>(Select all that apply.)

Behavioral Questions

Do you use any of the following products (either indoors or outdoors)?	☐ Tobacco products (e.g. cigarettes, cigars, pipes)<br>☐ Electronic cigarettes/vapor pens<br>☐ None of the above<br>☐ Prefer not to say<br>(Select all that apply.)
Does anyone in your shared living space use any of the following products (either indoors or outdoors)?	☐ Tobacco products (e.g. cigarettes, cigars, pipes)<br>☐ Electronic cigarettes/vapor pens<br>☐ None of the above<br>☐ Do not know<br>(Select all that apply.)
Have you received this season's influenza (flu) vaccine (since July 1, 2019) ? This includes both flu mist nasal spray and the flu shot.	◯ Yes<br>◯ No<br>◯ Do not know
What year did you get the flu shot or flu mist nasal spray this season (since July 1, 2019)?	◯ 2019<br>◯ 2020<br>◯ Do not know
What month did you get the flu shot or flu mist nasal spray this season (since July 1, 2019)?	◯ January<br>◯ February<br>◯ March<br>◯ April<br>◯ May<br>◯ June<br>◯ July<br>◯ August<br>◯ September<br>◯ October<br>◯ November<br>◯ December<br>◯ Do not know
How did you receive the flu vaccine this season (since July 1, 2019)?	◯ Injection (flu shot)<br>◯ Nasal spray (flu mist)
Where did you receive the flu vaccine this season (since July 1, 2019)?	◯ Medical clinic or hospital<br>◯ Pharmacy or drugstore<br>◯ Workplace<br>◯ School<br>◯ Other
What is the primary reason why you have not received the flu vaccine this season (since July 1, 2019)?	◯ Do not have time to get vaccinated<br>◯ Not required for work or school<br>◯ Not recommended by a doctor or healthcare worker<br>◯ Not covered by health insurance<br>◯ Not offered at a convenient location<br>◯ I am not worried about getting sick with the flu<br>◯ Concerns about vaccine safety or effectiveness<br>◯ I plan to get the flu vaccine<br>◯ None of the above
Did you receive the flu vaccine last season? (July 1, 2018 - July 1, 2019)	◯ Yes<br>◯ No

Have you ever been told by a healthcare provider that you have one of the following medical conditions?	☐ Asthma or reactive airway disease<br>☐ COPD/emphysema<br>☐ Chronic bronchitis<br>☐ Cancer<br>☐ Diabetes<br>☐ Heart disease (heart failure or heart attack)<br>☐ None of the above<br>☐ Do not know<br>☐ Prefer not to say<br>(Select all that apply.)

Recent Travel

In the past 7 days, have you visited a country other than the US?	◯ No, I have not traveled outside the US<br>◯ Yes

Country 1 visited

- ◯ Afghanistan
- ◯ Åland Islands
- ◯ Albania
- ◯ Algeria
- ◯ American Samoa
- ◯ Andorra
- ◯ Angola
- ◯ Anguilla
- ◯ Antarctica
- ◯ Antigua and Barbuda
- ◯ Argentina
- ◯ Armenia
- ◯ Aruba
- ◯ Australia
- ◯ Austria
- ◯ Azerbaijan
- ◯ Bahamas
- ◯ Bahrain
- ◯ Bangladesh
- ◯ Barbados
- ◯ Belarus
- ◯ Belgium
- ◯ Belize
- ◯ Benin
- ◯ Bermuda
- ◯ Bhutan
- ◯ Bolivia (Plurinational State of)
- ◯ Bonaire, Sint Eustatius and Saba
- ◯ Bosnia and Herzegovina
- ◯ Botswana
- ◯ Bouvet Island
- ◯ Brazil
- ◯ British Indian Ocean Territory
- ◯ Brunei Darussalam
- ◯ Bulgaria
- ◯ Burkina Faso
- ◯ Burundi
- ◯ Cabo Verde
- ◯ Cambodia
- ◯ Cameroon
- ◯ Canada
- ◯ Cayman Islands
- ◯ Central African Republic
- ◯ Chad
- ◯ Chile
- ◯ China
- ◯ Christmas Island
- ◯ Cocos (Keeling) Islands
- ◯ Colombia
- ◯ Comoros
- ◯ Congo, Republic of the
- ◯ Congo, Democratic Republic of the
- ◯ Cook Islands
- ◯ Costa Rica
- ◯ Côte d'Ivoire
- ◯ Croatia
- ◯ Cuba
- ◯ Curaçao
- ◯ Cyprus
- ◯ Czechia
- ◯ Denmark
- ◯ Djibouti
- ◯ Dominica
- ◯ Dominican Republic
- ◯ Ecuador
- ◯ Egypt
- ◯ El Salvador
- ◯ Equatorial Guinea
- ◯ Eritrea

◯ Estonia
◯ Eswatini
◯ Ethiopia
◯ Falkland Islands (Malvinas)
◯ Faroe Islands
◯ Fiji
◯ Finland
◯ France
◯ French Guiana
◯ French Polynesia
◯ French Southern Territories
◯ Gabon
◯ Gambia
◯ Georgia
◯ Germany
◯ Ghana
◯ Gibraltar
◯ Greece
◯ Greenland
◯ Grenada
◯ Guadeloupe
◯ Guam
◯ Guatemala
◯ Guernsey
◯ Guinea
◯ Guinea-Bissau
◯ Guyana
◯ Haiti
◯ Heard Island and McDonald Islands
◯ Holy See
◯ Honduras
◯ Hong Kong
◯ Hungary
◯ Iceland
◯ India
◯ Indonesia
◯ Iran (Islamic Republic of)
◯ Iraq
◯ Ireland
◯ Isle of Man
◯ Israel
◯ Italy
◯ Jamaica
◯ Japan
◯ Jersey
◯ Jordan
◯ Kazakhstan
◯ Kenya
◯ Kiribati
◯ Korea (Democratic People's Republic of)
◯ Korea, Republic of
◯ Kuwait
◯ Kyrgyzstan
◯ Lao People's Democratic Republic
◯ Latvia
◯ Lebanon
◯ Lesotho
◯ Liberia
◯ Libya
◯ Liechtenstein
◯ Lithuania
◯ Luxembourg
◯ Macao
◯ Madagascar
◯ Malawi
◯ Malaysia
◯ Maldives
◯ Mali
◯ Malta
◯ Marshall Islands
◯ Martinique

◯ Mauritania
◯ Mauritius
◯ Mayotte
◯ Mexico
◯ Micronesia (Federated States of)
◯ Moldova, Republic of
◯ Monaco
◯ Mongolia
◯ Montenegro
◯ Montserrat
◯ Morocco
◯ Mozambique
◯ Myanmar
◯ Namibia
◯ Nauru
◯ Nepal
◯ Netherlands
◯ New Caledonia
◯ New Zealand
◯ Nicaragua
◯ Niger
◯ Nigeria
◯ Niue
◯ Norfolk Island
◯ North Macedonia
◯ Northern Mariana Islands
◯ Norway
◯ Oman
◯ Pakistan
◯ Palau
◯ Palestine, State of
◯ Panama
◯ Papua New Guinea
◯ Paraguay
◯ Peru
◯ Philippines
◯ Pitcairn
◯ Poland
◯ Portugal
◯ Puerto Rico
◯ Qatar
◯ Réunion
◯ Romania
◯ Russian Federation
◯ Rwanda
◯ Saint Barthélemy
◯ Saint Helena, Ascension and Tristan da Cunha
◯ Saint Kitts and Nevis
◯ Saint Lucia
◯ Saint Martin (French part)
◯ Saint Pierre and Miquelon
◯ Saint Vincent and the Grenadines
◯ Samoa
◯ San Marino
◯ Sao Tome and Principe
◯ Saudi Arabia
◯ Senegal
◯ Serbia
◯ Seychelles
◯ Sierra Leone
◯ Singapore
◯ Sint Maarten (Dutch part)
◯ Slovakia
◯ Slovenia
◯ Solomon Islands
◯ Somalia
◯ South Africa
◯ South Georgia and the South Sandwich Islands
◯ South Sudan
◯ Spain
◯ Sri Lanka

◯ Sudan
◯ Suriname
◯ Svalbard and Jan Mayen
◯ Sweden
◯ Switzerland
◯ Syrian Arab Republic
◯ Taiwan
◯ Tajikistan
◯ Tanzania, United Republic of
◯ Thailand
◯ Timor-Leste
◯ Togo
◯ Tokelau
◯ Tonga
◯ Trinidad and Tobago
◯ Tunisia
◯ Turkey
◯ Turkmenistan
◯ Turks and Caicos Islands
◯ Tuvalu
◯ Uganda
◯ Ukraine
◯ United Arab Emirates
◯ United Kingdom of Great Britain and Northern Ireland
◯ United States of America
◯ United States Minor Outlying Islands
◯ Uruguay
◯ Uzbekistan
◯ Vanuatu
◯ Venezuela (Bolivarian Republic of)
◯ Viet Nam
◯ Virgin Islands (British)
◯ Virgin Islands (U.S.)
◯ Wallis and Futuna
◯ Western Sahara
◯ Yemen
◯ Zambia
◯ Zimbabwe

(Start typing and the country will be suggested)

Country 2 visited

◯ Afghanistan
◯ Åland Islands
◯ Albania
◯ Algeria
◯ American Samoa
◯ Andorra
◯ Angola
◯ Anguilla
◯ Antarctica
◯ Antigua and Barbuda
◯ Argentina
◯ Armenia
◯ Aruba
◯ Australia
◯ Austria
◯ Azerbaijan
◯ Bahamas
◯ Bahrain
◯ Bangladesh
◯ Barbados
◯ Belarus
◯ Belgium
◯ Belize
◯ Benin
◯ Bermuda
◯ Bhutan
◯ Bolivia (Plurinational State of)
◯ Bonaire, Sint Eustatius and Saba
◯ Bosnia and Herzegovina
◯ Botswana
◯ Bouvet Island
◯ Brazil
◯ British Indian Ocean Territory
◯ Brunei Darussalam
◯ Bulgaria
◯ Burkina Faso
◯ Burundi
◯ Cabo Verde
◯ Cambodia
◯ Cameroon
◯ Canada
◯ Cayman Islands
◯ Central African Republic
◯ Chad
◯ Chile
◯ China
◯ Christmas Island
◯ Cocos (Keeling) Islands
◯ Colombia
◯ Comoros
◯ Congo, Republic of the
◯ Congo, Democratic Republic of the
◯ Cook Islands
◯ Costa Rica
◯ Côte d'Ivoire
◯ Croatia
◯ Cuba
◯ Curaçao
◯ Cyprus
◯ Czechia
◯ Denmark
◯ Djibouti
◯ Dominica
◯ Dominican Republic
◯ Ecuador
◯ Egypt
◯ El Salvador
◯ Equatorial Guinea
◯ Eritrea

◯ Estonia
◯ Eswatini
◯ Ethiopia
◯ Falkland Islands (Malvinas)
◯ Faroe Islands
◯ Fiji
◯ Finland
◯ France
◯ French Guiana
◯ French Polynesia
◯ French Southern Territories
◯ Gabon
◯ Gambia
◯ Georgia
◯ Germany
◯ Ghana
◯ Gibraltar
◯ Greece
◯ Greenland
◯ Grenada
◯ Guadeloupe
◯ Guam
◯ Guatemala
◯ Guernsey
◯ Guinea
◯ Guinea-Bissau
◯ Guyana
◯ Haiti
◯ Heard Island and McDonald Islands
◯ Holy See
◯ Honduras
◯ Hong Kong
◯ Hungary
◯ Iceland
◯ India
◯ Indonesia
◯ Iran (Islamic Republic of)
◯ Iraq
◯ Ireland
◯ Isle of Man
◯ Israel
◯ Italy
◯ Jamaica
◯ Japan
◯ Jersey
◯ Jordan
◯ Kazakhstan
◯ Kenya
◯ Kiribati
◯ Korea (Democratic People's Republic of)
◯ Korea, Republic of
◯ Kuwait
◯ Kyrgyzstan
◯ Lao People's Democratic Republic
◯ Latvia
◯ Lebanon
◯ Lesotho
◯ Liberia
◯ Libya
◯ Liechtenstein
◯ Lithuania
◯ Luxembourg
◯ Macao
◯ Madagascar
◯ Malawi
◯ Malaysia
◯ Maldives
◯ Mali
◯ Malta
◯ Marshall Islands
◯ Martinique

◯ Mauritania
◯ Mauritius
◯ Mayotte
◯ Mexico
◯ Micronesia (Federated States of)
◯ Moldova, Republic of
◯ Monaco
◯ Mongolia
◯ Montenegro
◯ Montserrat
◯ Morocco
◯ Mozambique
◯ Myanmar
◯ Namibia
◯ Nauru
◯ Nepal
◯ Netherlands
◯ New Caledonia
◯ New Zealand
◯ Nicaragua
◯ Niger
◯ Nigeria
◯ Niue
◯ Norfolk Island
◯ North Macedonia
◯ Northern Mariana Islands
◯ Norway
◯ Oman
◯ Pakistan
◯ Palau
◯ Palestine, State of
◯ Panama
◯ Papua New Guinea
◯ Paraguay
◯ Peru
◯ Philippines
◯ Pitcairn
◯ Poland
◯ Portugal
◯ Puerto Rico
◯ Qatar
◯ Réunion
◯ Romania
◯ Russian Federation
◯ Rwanda
◯ Saint Barthélemy
◯ Saint Helena, Ascension and Tristan da Cunha
◯ Saint Kitts and Nevis
◯ Saint Lucia
◯ Saint Martin (French part)
◯ Saint Pierre and Miquelon
◯ Saint Vincent and the Grenadines
◯ Samoa
◯ San Marino
◯ Sao Tome and Principe
◯ Saudi Arabia
◯ Senegal
◯ Serbia
◯ Seychelles
◯ Sierra Leone
◯ Singapore
◯ Sint Maarten (Dutch part)
◯ Slovakia
◯ Slovenia
◯ Solomon Islands
◯ Somalia
◯ South Africa
◯ South Georgia and the South Sandwich Islands
◯ South Sudan
◯ Spain
◯ Sri Lanka

◯ Sudan
◯ Suriname
◯ Svalbard and Jan Mayen
◯ Sweden
◯ Switzerland
◯ Syrian Arab Republic
◯ Taiwan
◯ Tajikistan
◯ Tanzania, United Republic of
◯ Thailand
◯ Timor-Leste
◯ Togo
◯ Tokelau
◯ Tonga
◯ Trinidad and Tobago
◯ Tunisia
◯ Turkey
◯ Turkmenistan
◯ Turks and Caicos Islands
◯ Tuvalu
◯ Uganda
◯ Ukraine
◯ United Arab Emirates
◯ United Kingdom of Great Britain and Northern Ireland
◯ United States of America
◯ United States Minor Outlying Islands
◯ Uruguay
◯ Uzbekistan
◯ Vanuatu
◯ Venezuela (Bolivarian Republic of)
◯ Viet Nam
◯ Virgin Islands (British)
◯ Virgin Islands (U.S.)
◯ Wallis and Futuna
◯ Western Sahara
◯ Yemen
◯ Zambia
◯ Zimbabwe

(Start typing and the country will be suggested. If you only visited 1 country move to the next question)

Country 3 visited	◯ Afghanistan<br>◯ Åland Islands<br>◯ Albania<br>◯ Algeria<br>◯ American Samoa<br>◯ Andorra<br>◯ Angola<br>◯ Anguilla<br>◯ Antarctica<br>◯ Antigua and Barbuda<br>◯ Argentina<br>◯ Armenia<br>◯ Aruba<br>◯ Australia<br>◯ Austria<br>◯ Azerbaijan<br>◯ Bahamas<br>◯ Bahrain<br>◯ Bangladesh<br>◯ Barbados<br>◯ Belarus<br>◯ Belgium<br>◯ Belize<br>◯ Benin<br>◯ Bermuda<br>◯ Bhutan<br>◯ Bolivia (Plurinational State of)<br>◯ Bonaire, Sint Eustatius and Saba<br>◯ Bosnia and Herzegovina<br>◯ Botswana<br>◯ Bouvet Island<br>◯ Brazil<br>◯ British Indian Ocean Territory<br>◯ Brunei Darussalam<br>◯ Bulgaria<br>◯ Burkina Faso<br>◯ Burundi<br>◯ Cabo Verde<br>◯ Cambodia<br>◯ Cameroon<br>◯ Canada<br>◯ Cayman Islands<br>◯ Central African Republic<br>◯ Chad<br>◯ Chile<br>◯ China<br>◯ Christmas Island<br>◯ Cocos (Keeling) Islands<br>◯ Colombia<br>◯ Comoros<br>◯ Congo, Republic of the<br>◯ Congo, Democratic Republic of the<br>◯ Cook Islands<br>◯ Costa Rica<br>◯ Côte d'Ivoire<br>◯ Croatia<br>◯ Cuba<br>◯ Curaçao<br>◯ Cyprus<br>◯ Czechia<br>◯ Denmark<br>◯ Djibouti<br>◯ Dominica<br>◯ Dominican Republic<br>◯ Ecuador<br>◯ Egypt<br>◯ El Salvador<br>◯ Equatorial Guinea<br>◯ Eritrea

◯ Estonia
◯ Eswatini
◯ Ethiopia
◯ Falkland Islands (Malvinas)
◯ Faroe Islands
◯ Fiji
◯ Finland
◯ France
◯ French Guiana
◯ French Polynesia
◯ French Southern Territories
◯ Gabon
◯ Gambia
◯ Georgia
◯ Germany
◯ Ghana
◯ Gibraltar
◯ Greece
◯ Greenland
◯ Grenada
◯ Guadeloupe
◯ Guam
◯ Guatemala
◯ Guernsey
◯ Guinea
◯ Guinea-Bissau
◯ Guyana
◯ Haiti
◯ Heard Island and McDonald Islands
◯ Holy See
◯ Honduras
◯ Hong Kong
◯ Hungary
◯ Iceland
◯ India
◯ Indonesia
◯ Iran (Islamic Republic of)
◯ Iraq
◯ Ireland
◯ Isle of Man
◯ Israel
◯ Italy
◯ Jamaica
◯ Japan
◯ Jersey
◯ Jordan
◯ Kazakhstan
◯ Kenya
◯ Kiribati
◯ Korea (Democratic People's Republic of)
◯ Korea, Republic of
◯ Kuwait
◯ Kyrgyzstan
◯ Lao People's Democratic Republic
◯ Latvia
◯ Lebanon
◯ Lesotho
◯ Liberia
◯ Libya
◯ Liechtenstein
◯ Lithuania
◯ Luxembourg
◯ Macao
◯ Madagascar
◯ Malawi
◯ Malaysia
◯ Maldives
◯ Mali
◯ Malta
◯ Marshall Islands
◯ Martinique

◯ Mauritania
◯ Mauritius
◯ Mayotte
◯ Mexico
◯ Micronesia (Federated States of)
◯ Moldova, Republic of
◯ Monaco
◯ Mongolia
◯ Montenegro
◯ Montserrat
◯ Morocco
◯ Mozambique
◯ Myanmar
◯ Namibia
◯ Nauru
◯ Nepal
◯ Netherlands
◯ New Caledonia
◯ New Zealand
◯ Nicaragua
◯ Niger
◯ Nigeria
◯ Niue
◯ Norfolk Island
◯ North Macedonia
◯ Northern Mariana Islands
◯ Norway
◯ Oman
◯ Pakistan
◯ Palau
◯ Palestine, State of
◯ Panama
◯ Papua New Guinea
◯ Paraguay
◯ Peru
◯ Philippines
◯ Pitcairn
◯ Poland
◯ Portugal
◯ Puerto Rico
◯ Qatar
◯ Réunion
◯ Romania
◯ Russian Federation
◯ Rwanda
◯ Saint Barthélemy
◯ Saint Helena, Ascension and Tristan da Cunha
◯ Saint Kitts and Nevis
◯ Saint Lucia
◯ Saint Martin (French part)
◯ Saint Pierre and Miquelon
◯ Saint Vincent and the Grenadines
◯ Samoa
◯ San Marino
◯ Sao Tome and Principe
◯ Saudi Arabia
◯ Senegal
◯ Serbia
◯ Seychelles
◯ Sierra Leone
◯ Singapore
◯ Sint Maarten (Dutch part)
◯ Slovakia
◯ Slovenia
◯ Solomon Islands
◯ Somalia
◯ South Africa
◯ South Georgia and the South Sandwich Islands
◯ South Sudan
◯ Spain
◯ Sri Lanka

◯ Sudan
◯ Suriname
◯ Svalbard and Jan Mayen
◯ Sweden
◯ Switzerland
◯ Syrian Arab Republic
◯ Taiwan
◯ Tajikistan
◯ Tanzania, United Republic of
◯ Thailand
◯ Timor-Leste
◯ Togo
◯ Tokelau
◯ Tonga
◯ Trinidad and Tobago
◯ Tunisia
◯ Turkey
◯ Turkmenistan
◯ Turks and Caicos Islands
◯ Tuvalu
◯ Uganda
◯ Ukraine
◯ United Arab Emirates
◯ United Kingdom of Great Britain and Northern Ireland
◯ United States of America
◯ United States Minor Outlying Islands
◯ Uruguay
◯ Uzbekistan
◯ Vanuatu
◯ Venezuela (Bolivarian Republic of)
◯ Viet Nam
◯ Virgin Islands (British)
◯ Virgin Islands (U.S.)
◯ Wallis and Futuna
◯ Western Sahara
◯ Yemen
◯ Zambia
◯ Zimbabwe

(Start typing and the country will be suggested. If you only visited 2 countries move to the next question.)

Country 4 visited

- ◯ Afghanistan
- ◯ Åland Islands
- ◯ Albania
- ◯ Algeria
- ◯ American Samoa
- ◯ Andorra
- ◯ Angola
- ◯ Anguilla
- ◯ Antarctica
- ◯ Antigua and Barbuda
- ◯ Argentina
- ◯ Armenia
- ◯ Aruba
- ◯ Australia
- ◯ Austria
- ◯ Azerbaijan
- ◯ Bahamas
- ◯ Bahrain
- ◯ Bangladesh
- ◯ Barbados
- ◯ Belarus
- ◯ Belgium
- ◯ Belize
- ◯ Benin
- ◯ Bermuda
- ◯ Bhutan
- ◯ Bolivia (Plurinational State of)
- ◯ Bonaire, Sint Eustatius and Saba
- ◯ Bosnia and Herzegovina
- ◯ Botswana
- ◯ Bouvet Island
- ◯ Brazil
- ◯ British Indian Ocean Territory
- ◯ Brunei Darussalam
- ◯ Bulgaria
- ◯ Burkina Faso
- ◯ Burundi
- ◯ Cabo Verde
- ◯ Cambodia
- ◯ Cameroon
- ◯ Canada
- ◯ Cayman Islands
- ◯ Central African Republic
- ◯ Chad
- ◯ Chile
- ◯ China
- ◯ Christmas Island
- ◯ Cocos (Keeling) Islands
- ◯ Colombia
- ◯ Comoros
- ◯ Congo, Republic of the
- ◯ Congo, Democratic Republic of the
- ◯ Cook Islands
- ◯ Costa Rica
- ◯ Côte d'Ivoire
- ◯ Croatia
- ◯ Cuba
- ◯ Curaçao
- ◯ Cyprus
- ◯ Czechia
- ◯ Denmark
- ◯ Djibouti
- ◯ Dominica
- ◯ Dominican Republic
- ◯ Ecuador
- ◯ Egypt
- ◯ El Salvador
- ◯ Equatorial Guinea
- ◯ Eritrea

◯ Estonia
◯ Eswatini
◯ Ethiopia
◯ Falkland Islands (Malvinas)
◯ Faroe Islands
◯ Fiji
◯ Finland
◯ France
◯ French Guiana
◯ French Polynesia
◯ French Southern Territories
◯ Gabon
◯ Gambia
◯ Georgia
◯ Germany
◯ Ghana
◯ Gibraltar
◯ Greece
◯ Greenland
◯ Grenada
◯ Guadeloupe
◯ Guam
◯ Guatemala
◯ Guernsey
◯ Guinea
◯ Guinea-Bissau
◯ Guyana
◯ Haiti
◯ Heard Island and McDonald Islands
◯ Holy See
◯ Honduras
◯ Hong Kong
◯ Hungary
◯ Iceland
◯ India
◯ Indonesia
◯ Iran (Islamic Republic of)
◯ Iraq
◯ Ireland
◯ Isle of Man
◯ Israel
◯ Italy
◯ Jamaica
◯ Japan
◯ Jersey
◯ Jordan
◯ Kazakhstan
◯ Kenya
◯ Kiribati
◯ Korea (Democratic People's Republic of)
◯ Korea, Republic of
◯ Kuwait
◯ Kyrgyzstan
◯ Lao People's Democratic Republic
◯ Latvia
◯ Lebanon
◯ Lesotho
◯ Liberia
◯ Libya
◯ Liechtenstein
◯ Lithuania
◯ Luxembourg
◯ Macao
◯ Madagascar
◯ Malawi
◯ Malaysia
◯ Maldives
◯ Mali
◯ Malta
◯ Marshall Islands
◯ Martinique

◯ Mauritania
◯ Mauritius
◯ Mayotte
◯ Mexico
◯ Micronesia (Federated States of)
◯ Moldova, Republic of
◯ Monaco
◯ Mongolia
◯ Montenegro
◯ Montserrat
◯ Morocco
◯ Mozambique
◯ Myanmar
◯ Namibia
◯ Nauru
◯ Nepal
◯ Netherlands
◯ New Caledonia
◯ New Zealand
◯ Nicaragua
◯ Niger
◯ Nigeria
◯ Niue
◯ Norfolk Island
◯ North Macedonia
◯ Northern Mariana Islands
◯ Norway
◯ Oman
◯ Pakistan
◯ Palau
◯ Palestine, State of
◯ Panama
◯ Papua New Guinea
◯ Paraguay
◯ Peru
◯ Philippines
◯ Pitcairn
◯ Poland
◯ Portugal
◯ Puerto Rico
◯ Qatar
◯ Réunion
◯ Romania
◯ Russian Federation
◯ Rwanda
◯ Saint Barthélemy
◯ Saint Helena, Ascension and Tristan da Cunha
◯ Saint Kitts and Nevis
◯ Saint Lucia
◯ Saint Martin (French part)
◯ Saint Pierre and Miquelon
◯ Saint Vincent and the Grenadines
◯ Samoa
◯ San Marino
◯ Sao Tome and Principe
◯ Saudi Arabia
◯ Senegal
◯ Serbia
◯ Seychelles
◯ Sierra Leone
◯ Singapore
◯ Sint Maarten (Dutch part)
◯ Slovakia
◯ Slovenia
◯ Solomon Islands
◯ Somalia
◯ South Africa
◯ South Georgia and the South Sandwich Islands
◯ South Sudan
◯ Spain
◯ Sri Lanka

◯ Sudan
◯ Suriname
◯ Svalbard and Jan Mayen
◯ Sweden
◯ Switzerland
◯ Syrian Arab Republic
◯ Taiwan
◯ Tajikistan
◯ Tanzania, United Republic of
◯ Thailand
◯ Timor-Leste
◯ Togo
◯ Tokelau
◯ Tonga
◯ Trinidad and Tobago
◯ Tunisia
◯ Turkey
◯ Turkmenistan
◯ Turks and Caicos Islands
◯ Tuvalu
◯ Uganda
◯ Ukraine
◯ United Arab Emirates
◯ United Kingdom of Great Britain and Northern Ireland
◯ United States of America
◯ United States Minor Outlying Islands
◯ Uruguay
◯ Uzbekistan
◯ Vanuatu
◯ Venezuela (Bolivarian Republic of)
◯ Viet Nam
◯ Virgin Islands (British)
◯ Virgin Islands (U.S.)
◯ Wallis and Futuna
◯ Western Sahara
◯ Yemen
◯ Zambia
◯ Zimbabwe

(Start typing and the country will be suggested. If you only visited 3 countries move to the next question.)

Country 5 visited

- ◯ Afghanistan
- ◯ Åland Islands
- ◯ Albania
- ◯ Algeria
- ◯ American Samoa
- ◯ Andorra
- ◯ Angola
- ◯ Anguilla
- ◯ Antarctica
- ◯ Antigua and Barbuda
- ◯ Argentina
- ◯ Armenia
- ◯ Aruba
- ◯ Australia
- ◯ Austria
- ◯ Azerbaijan
- ◯ Bahamas
- ◯ Bahrain
- ◯ Bangladesh
- ◯ Barbados
- ◯ Belarus
- ◯ Belgium
- ◯ Belize
- ◯ Benin
- ◯ Bermuda
- ◯ Bhutan
- ◯ Bolivia (Plurinational State of)
- ◯ Bonaire, Sint Eustatius and Saba
- ◯ Bosnia and Herzegovina
- ◯ Botswana
- ◯ Bouvet Island
- ◯ Brazil
- ◯ British Indian Ocean Territory
- ◯ Brunei Darussalam
- ◯ Bulgaria
- ◯ Burkina Faso
- ◯ Burundi
- ◯ Cabo Verde
- ◯ Cambodia
- ◯ Cameroon
- ◯ Canada
- ◯ Cayman Islands
- ◯ Central African Republic
- ◯ Chad
- ◯ Chile
- ◯ China
- ◯ Christmas Island
- ◯ Cocos (Keeling) Islands
- ◯ Colombia
- ◯ Comoros
- ◯ Congo, Republic of the
- ◯ Congo, Democratic Republic of the
- ◯ Cook Islands
- ◯ Costa Rica
- ◯ Côte d'Ivoire
- ◯ Croatia
- ◯ Cuba
- ◯ Curaçao
- ◯ Cyprus
- ◯ Czechia
- ◯ Denmark
- ◯ Djibouti
- ◯ Dominica
- ◯ Dominican Republic
- ◯ Ecuador
- ◯ Egypt
- ◯ El Salvador
- ◯ Equatorial Guinea
- ◯ Eritrea

◯ Estonia
◯ Eswatini
◯ Ethiopia
◯ Falkland Islands (Malvinas)
◯ Faroe Islands
◯ Fiji
◯ Finland
◯ France
◯ French Guiana
◯ French Polynesia
◯ French Southern Territories
◯ Gabon
◯ Gambia
◯ Georgia
◯ Germany
◯ Ghana
◯ Gibraltar
◯ Greece
◯ Greenland
◯ Grenada
◯ Guadeloupe
◯ Guam
◯ Guatemala
◯ Guernsey
◯ Guinea
◯ Guinea-Bissau
◯ Guyana
◯ Haiti
◯ Heard Island and McDonald Islands
◯ Holy See
◯ Honduras
◯ Hong Kong
◯ Hungary
◯ Iceland
◯ India
◯ Indonesia
◯ Iran (Islamic Republic of)
◯ Iraq
◯ Ireland
◯ Isle of Man
◯ Israel
◯ Italy
◯ Jamaica
◯ Japan
◯ Jersey
◯ Jordan
◯ Kazakhstan
◯ Kenya
◯ Kiribati
◯ Korea (Democratic People's Republic of)
◯ Korea, Republic of
◯ Kuwait
◯ Kyrgyzstan
◯ Lao People's Democratic Republic
◯ Latvia
◯ Lebanon
◯ Lesotho
◯ Liberia
◯ Libya
◯ Liechtenstein
◯ Lithuania
◯ Luxembourg
◯ Macao
◯ Madagascar
◯ Malawi
◯ Malaysia
◯ Maldives
◯ Mali
◯ Malta
◯ Marshall Islands
◯ Martinique

◯ Mauritania
◯ Mauritius
◯ Mayotte
◯ Mexico
◯ Micronesia (Federated States of)
◯ Moldova, Republic of
◯ Monaco
◯ Mongolia
◯ Montenegro
◯ Montserrat
◯ Morocco
◯ Mozambique
◯ Myanmar
◯ Namibia
◯ Nauru
◯ Nepal
◯ Netherlands
◯ New Caledonia
◯ New Zealand
◯ Nicaragua
◯ Niger
◯ Nigeria
◯ Niue
◯ Norfolk Island
◯ North Macedonia
◯ Northern Mariana Islands
◯ Norway
◯ Oman
◯ Pakistan
◯ Palau
◯ Palestine, State of
◯ Panama
◯ Papua New Guinea
◯ Paraguay
◯ Peru
◯ Philippines
◯ Pitcairn
◯ Poland
◯ Portugal
◯ Puerto Rico
◯ Qatar
◯ Réunion
◯ Romania
◯ Russian Federation
◯ Rwanda
◯ Saint Barthélemy
◯ Saint Helena, Ascension and Tristan da Cunha
◯ Saint Kitts and Nevis
◯ Saint Lucia
◯ Saint Martin (French part)
◯ Saint Pierre and Miquelon
◯ Saint Vincent and the Grenadines
◯ Samoa
◯ San Marino
◯ Sao Tome and Principe
◯ Saudi Arabia
◯ Senegal
◯ Serbia
◯ Seychelles
◯ Sierra Leone
◯ Singapore
◯ Sint Maarten (Dutch part)
◯ Slovakia
◯ Slovenia
◯ Solomon Islands
◯ Somalia
◯ South Africa
◯ South Georgia and the South Sandwich Islands
◯ South Sudan
◯ Spain
◯ Sri Lanka

◯ Sudan
◯ Suriname
◯ Svalbard and Jan Mayen
◯ Sweden
◯ Switzerland
◯ Syrian Arab Republic
◯ Taiwan
◯ Tajikistan
◯ Tanzania, United Republic of
◯ Thailand
◯ Timor-Leste
◯ Togo
◯ Tokelau
◯ Tonga
◯ Trinidad and Tobago
◯ Tunisia
◯ Turkey
◯ Turkmenistan
◯ Turks and Caicos Islands
◯ Tuvalu
◯ Uganda
◯ Ukraine
◯ United Arab Emirates
◯ United Kingdom of Great Britain and Northern Ireland
◯ United States of America
◯ United States Minor Outlying Islands
◯ Uruguay
◯ Uzbekistan
◯ Vanuatu
◯ Venezuela (Bolivarian Republic of)
◯ Viet Nam
◯ Virgin Islands (British)
◯ Virgin Islands (U.S.)
◯ Wallis and Futuna
◯ Western Sahara
◯ Yemen
◯ Zambia
◯ Zimbabwe

(Start typing and the country will be suggested. If you only visited 4 countries move to the next question.)

In the past 7 days have you traveled outside the state of Washington?

◯ No, I have not traveled outside Washington
◯ Yes

State 1

◯ Alabama
◯ Alaska
◯ Arizona
◯ Arkansas
◯ California
◯ Colorado
◯ Connecticut
◯ Delaware
◯ District of Columbia
◯ Florida
◯ Georgia
◯ Hawaii
◯ Idaho
◯ Illinois
◯ Indiana
◯ Iowa
◯ Kansas
◯ Kentucky
◯ Louisiana
◯ Maine
◯ Maryland
◯ Massachusetts
◯ Michigan
◯ Minnesota
◯ Mississippi
◯ Missouri
◯ Montana
◯ Nebraska
◯ Nevada
◯ New Hampshire
◯ New Jersey
◯ New Mexico
◯ New York
◯ North Carolina
◯ North Dakota
◯ Ohio
◯ Oklahoma
◯ Oregon
◯ Pennsylvania
◯ Rhode Island
◯ South Carolina
◯ South Dakota
◯ Tennessee
◯ Texas
◯ Utah
◯ Vermont
◯ Virginia
◯ Washington
◯ West Virginia
◯ Wisconsin
◯ Wyoming
◯ American Samoa
◯ Guam
◯ Northern Mariana Islands
◯ Puerto Rico
◯ U.S. Minor Outlying Islands
◯ U.S. Virgin Islands
(Start typing and the state will be suggested.)

State 2

◯ Alabama
◯ Alaska
◯ Arizona
◯ Arkansas
◯ California
◯ Colorado
◯ Connecticut
◯ Delaware
◯ District of Columbia
◯ Florida
◯ Georgia
◯ Hawaii
◯ Idaho
◯ Illinois
◯ Indiana
◯ Iowa
◯ Kansas
◯ Kentucky
◯ Louisiana
◯ Maine
◯ Maryland
◯ Massachusetts
◯ Michigan
◯ Minnesota
◯ Mississippi
◯ Missouri
◯ Montana
◯ Nebraska
◯ Nevada
◯ New Hampshire
◯ New Jersey
◯ New Mexico
◯ New York
◯ North Carolina
◯ North Dakota
◯ Ohio
◯ Oklahoma
◯ Oregon
◯ Pennsylvania
◯ Rhode Island
◯ South Carolina
◯ South Dakota
◯ Tennessee
◯ Texas
◯ Utah
◯ Vermont
◯ Virginia
◯ Washington
◯ West Virginia
◯ Wisconsin
◯ Wyoming
◯ American Samoa
◯ Guam
◯ Northern Mariana Islands
◯ Puerto Rico
◯ U.S. Minor Outlying Islands
◯ U.S. Virgin Islands

(Start typing and the state will be suggested. If you only visited 1 state move to the next question.)

State 3

◯ Alabama
◯ Alaska
◯ Arizona
◯ Arkansas
◯ California
◯ Colorado
◯ Connecticut
◯ Delaware
◯ District of Columbia
◯ Florida
◯ Georgia
◯ Hawaii
◯ Idaho
◯ Illinois
◯ Indiana
◯ Iowa
◯ Kansas
◯ Kentucky
◯ Louisiana
◯ Maine
◯ Maryland
◯ Massachusetts
◯ Michigan
◯ Minnesota
◯ Mississippi
◯ Missouri
◯ Montana
◯ Nebraska
◯ Nevada
◯ New Hampshire
◯ New Jersey
◯ New Mexico
◯ New York
◯ North Carolina
◯ North Dakota
◯ Ohio
◯ Oklahoma
◯ Oregon
◯ Pennsylvania
◯ Rhode Island
◯ South Carolina
◯ South Dakota
◯ Tennessee
◯ Texas
◯ Utah
◯ Vermont
◯ Virginia
◯ Washington
◯ West Virginia
◯ Wisconsin
◯ Wyoming
◯ American Samoa
◯ Guam
◯ Northern Mariana Islands
◯ Puerto Rico
◯ U.S. Minor Outlying Islands
◯ U.S. Virgin Islands

(Start typing and the state will be suggested. If you only visited 2 states move to the next question.)

State 4

◯ Alabama
◯ Alaska
◯ Arizona
◯ Arkansas
◯ California
◯ Colorado
◯ Connecticut
◯ Delaware
◯ District of Columbia
◯ Florida
◯ Georgia
◯ Hawaii
◯ Idaho
◯ Illinois
◯ Indiana
◯ Iowa
◯ Kansas
◯ Kentucky
◯ Louisiana
◯ Maine
◯ Maryland
◯ Massachusetts
◯ Michigan
◯ Minnesota
◯ Mississippi
◯ Missouri
◯ Montana
◯ Nebraska
◯ Nevada
◯ New Hampshire
◯ New Jersey
◯ New Mexico
◯ New York
◯ North Carolina
◯ North Dakota
◯ Ohio
◯ Oklahoma
◯ Oregon
◯ Pennsylvania
◯ Rhode Island
◯ South Carolina
◯ South Dakota
◯ Tennessee
◯ Texas
◯ Utah
◯ Vermont
◯ Virginia
◯ Washington
◯ West Virginia
◯ Wisconsin
◯ Wyoming
◯ American Samoa
◯ Guam
◯ Northern Mariana Islands
◯ Puerto Rico
◯ U.S. Minor Outlying Islands
◯ U.S. Virgin Islands

(Start typing and the state will be suggested. If you only visited 3 states move to the next question.)

State 5

◯ Alabama
◯ Alaska
◯ Arizona
◯ Arkansas
◯ California
◯ Colorado
◯ Connecticut
◯ Delaware
◯ District of Columbia
◯ Florida
◯ Georgia
◯ Hawaii
◯ Idaho
◯ Illinois
◯ Indiana
◯ Iowa
◯ Kansas
◯ Kentucky
◯ Louisiana
◯ Maine
◯ Maryland
◯ Massachusetts
◯ Michigan
◯ Minnesota
◯ Mississippi
◯ Missouri
◯ Montana
◯ Nebraska
◯ Nevada
◯ New Hampshire
◯ New Jersey
◯ New Mexico
◯ New York
◯ North Carolina
◯ North Dakota
◯ Ohio
◯ Oklahoma
◯ Oregon
◯ Pennsylvania
◯ Rhode Island
◯ South Carolina
◯ South Dakota
◯ Tennessee
◯ Texas
◯ Utah
◯ Vermont
◯ Virginia
◯ Washington
◯ West Virginia
◯ Wisconsin
◯ Wyoming
◯ American Samoa
◯ Guam
◯ Northern Mariana Islands
◯ Puerto Rico
◯ U.S. Minor Outlying Islands
◯ U.S. Virgin Islands

(Start typing and the state will be suggested. If you only visited 4 states move to the next question.)

Part 2: Nasal Swab Collection

Follow the instructions on the card inside of your swab kit to collect a nasal swab.

Tips: If you have a lot of snot, make sure to blow your nose first. This helps us get a better sample from your nasal swab! Insert the tip of the swab about half-way up your nose. Swirl the swab around in your nasal passage for about 5 seconds. Once you have taken the nasal swab, put the swab in the red top sample tube. Break off the top of the nasal swab by bending the handle back-and-forth a couple of times. After collecting your nasal swab, tightly screw the red top back onto the tube. Write today's date on the label on the tube. Next, answer the following set of questions about your barcode.

Is [pre_scan_barcode] the barcode on your sample tube?

Your barcode is an 8 chararcter code which can be found on the GREEN label on your sample tube, return envelope, or 'Quick Start Instruction' card.

All 3 labels are identical and contain your unique participant barcode.

◯ Yes
◯ No

Please enter your barcode.

Your barcode is an 8 chararcter code which can be found on the GREEN label on your sample tube, box, or 'Return of Results' card.

All 3 labels are identical and contain your unique participant barcode.

____

Please reenter your barcode.

____

Important! Please double-check: are [utm_tube_barcode_2] and [reenter_barcode] the same barcode?

◯ Yes
◯ No

You previously entered an incorrect barcode. Please go back and enter the correct barcode found on the GREEN label.

Send your nasal swab back to us:

Place the sample tube in the plastic bag provided and tightly seal the bag. Next, place the plastic bag containing your tube in the Seattle Flu Study box and close it. Lastly, place the box in the mailer envelope with the return postage on it and seal the envelope. After the envelope containing your nasal swab is all ready to go, return it to us by one of the following options:

1) Place it in your mailbox 2) Drop it in a USPS blue collection box (Link: USPS Blue Box Locations) 3) Schedule a USPS home pick-up (Link: USPS Schedule a Pickup) 4) Drop it off at a US Post Office

---

Thank you! If you have any questions, please email us at SeattleFlu@uw.edu or call us (206) 221-4588 during normal business hours.

Illness Questionnaire Date

____
