# Supplementary material for: “I probably shouldn’t go in today”: Inequitable access to paid sick leave and its impacts on health behaviors during the emergence of COVID-19 in the Seattle area
Source: PLoS One. 2024 Sep 10;19(9):e0307734. doi: 10.1371/journal.pone.0307734 (PMC11386467; doi:10.1371/journal.pone.0307734)
Supplement: S2 File — (ZIP) [file pone.0307734.s002.zip › Copy of AppendixD_SFS_Week_FollowUp_Survey.pdf]

One Week Follow-up Survey

The following questions refer to your illness from [enrollment_date].

Are you fully recovered from your illness?	◯ Yes<br>◯ No<br>◯ Do Not Know
How many days did your illness last?	______________________
Have you visited a country other than the US since [enrollment_date]?	◯ Yes<br>◯ No - I have not traveled outside the US

Country 1 visited

- ◯ Afghanistan
- ◯ Åland Islands
- ◯ Albania
- ◯ Algeria
- ◯ American Samoa
- ◯ Andorra
- ◯ Angola
- ◯ Anguilla
- ◯ Antarctica
- ◯ Antigua and Barbuda
- ◯ Argentina
- ◯ Armenia
- ◯ Aruba
- ◯ Australia
- ◯ Austria
- ◯ Azerbaijan
- ◯ Bahamas
- ◯ Bahrain
- ◯ Bangladesh
- ◯ Barbados
- ◯ Belarus
- ◯ Belgium
- ◯ Belize
- ◯ Benin
- ◯ Bermuda
- ◯ Bhutan
- ◯ Bolivia (Plurinational State of)
- ◯ Bonaire, Sint Eustatius and Saba
- ◯ Bosnia and Herzegovina
- ◯ Botswana
- ◯ Bouvet Island
- ◯ Brazil
- ◯ British Indian Ocean Territory
- ◯ Brunei Darussalam
- ◯ Bulgaria
- ◯ Burkina Faso
- ◯ Burundi
- ◯ Cabo Verde
- ◯ Cambodia
- ◯ Cameroon
- ◯ Canada
- ◯ Cayman Islands
- ◯ Central African Republic
- ◯ Chad
- ◯ Chile
- ◯ China
- ◯ Christmas Island
- ◯ Cocos (Keeling) Islands
- ◯ Colombia
- ◯ Comoros
- ◯ Congo, Republic of the
- ◯ Congo, Democratic Republic of the
- ◯ Cook Islands
- ◯ Costa Rica
- ◯ Côte d'Ivoire
- ◯ Croatia
- ◯ Cuba
- ◯ Curaçao
- ◯ Cyprus
- ◯ Czechia
- ◯ Denmark
- ◯ Djibouti
- ◯ Dominica
- ◯ Dominican Republic
- ◯ Ecuador
- ◯ Egypt
- ◯ El Salvador
- ◯ Equatorial Guinea
- ◯ Eritrea

◯ Estonia
◯ Eswatini
◯ Ethiopia
◯ Falkland Islands (Malvinas)
◯ Faroe Islands
◯ Fiji
◯ Finland
◯ France
◯ French Guiana
◯ French Polynesia
◯ French Southern Territories
◯ Gabon
◯ Gambia
◯ Georgia
◯ Germany
◯ Ghana
◯ Gibraltar
◯ Greece
◯ Greenland
◯ Grenada
◯ Guadeloupe
◯ Guam
◯ Guatemala
◯ Guernsey
◯ Guinea
◯ Guinea-Bissau
◯ Guyana
◯ Haiti
◯ Heard Island and McDonald Islands
◯ Holy See
◯ Honduras
◯ Hong Kong
◯ Hungary
◯ Iceland
◯ India
◯ Indonesia
◯ Iran (Islamic Republic of)
◯ Iraq
◯ Ireland
◯ Isle of Man
◯ Israel
◯ Italy
◯ Jamaica
◯ Japan
◯ Jersey
◯ Jordan
◯ Kazakhstan
◯ Kenya
◯ Kiribati
◯ Korea (Democratic People's Republic of)
◯ Korea, Republic of
◯ Kuwait
◯ Kyrgyzstan
◯ Lao People's Democratic Republic
◯ Latvia
◯ Lebanon
◯ Lesotho
◯ Liberia
◯ Libya
◯ Liechtenstein
◯ Lithuania
◯ Luxembourg
◯ Macao
◯ Madagascar
◯ Malawi
◯ Malaysia
◯ Maldives
◯ Mali
◯ Malta
◯ Marshall Islands
◯ Martinique

◯ Mauritania
◯ Mauritius
◯ Mayotte
◯ Mexico
◯ Micronesia (Federated States of)
◯ Moldova, Republic of
◯ Monaco
◯ Mongolia
◯ Montenegro
◯ Montserrat
◯ Morocco
◯ Mozambique
◯ Myanmar
◯ Namibia
◯ Nauru
◯ Nepal
◯ Netherlands
◯ New Caledonia
◯ New Zealand
◯ Nicaragua
◯ Niger
◯ Nigeria
◯ Niue
◯ Norfolk Island
◯ North Macedonia
◯ Northern Mariana Islands
◯ Norway
◯ Oman
◯ Pakistan
◯ Palau
◯ Palestine, State of
◯ Panama
◯ Papua New Guinea
◯ Paraguay
◯ Peru
◯ Philippines
◯ Pitcairn
◯ Poland
◯ Portugal
◯ Puerto Rico
◯ Qatar
◯ Réunion
◯ Romania
◯ Russian Federation
◯ Rwanda
◯ Saint Barthélemy
◯ Saint Helena, Ascension and Tristan da Cunha
◯ Saint Kitts and Nevis
◯ Saint Lucia
◯ Saint Martin (French part)
◯ Saint Pierre and Miquelon
◯ Saint Vincent and the Grenadines
◯ Samoa
◯ San Marino
◯ Sao Tome and Principe
◯ Saudi Arabia
◯ Senegal
◯ Serbia
◯ Seychelles
◯ Sierra Leone
◯ Singapore
◯ Sint Maarten (Dutch part)
◯ Slovakia
◯ Slovenia
◯ Solomon Islands
◯ Somalia
◯ South Africa
◯ South Georgia and the South Sandwich Islands
◯ South Sudan
◯ Spain
◯ Sri Lanka

◯ Sudan
◯ Suriname
◯ Svalbard and Jan Mayen
◯ Sweden
◯ Switzerland
◯ Syrian Arab Republic
◯ Taiwan
◯ Tajikistan
◯ Tanzania, United Republic of
◯ Thailand
◯ Timor-Leste
◯ Togo
◯ Tokelau
◯ Tonga
◯ Trinidad and Tobago
◯ Tunisia
◯ Turkey
◯ Turkmenistan
◯ Turks and Caicos Islands
◯ Tuvalu
◯ Uganda
◯ Ukraine
◯ United Arab Emirates
◯ United Kingdom of Great Britain and Northern Ireland
◯ United States of America
◯ United States Minor Outlying Islands
◯ Uruguay
◯ Uzbekistan
◯ Vanuatu
◯ Venezuela (Bolivarian Republic of)
◯ Viet Nam
◯ Virgin Islands (British)
◯ Virgin Islands (U.S.)
◯ Wallis and Futuna
◯ Western Sahara
◯ Yemen
◯ Zambia
◯ Zimbabwe

(Start typing and the country will be suggested)

Country 2 visited

- ◯ Afghanistan
- ◯ Åland Islands
- ◯ Albania
- ◯ Algeria
- ◯ American Samoa
- ◯ Andorra
- ◯ Angola
- ◯ Anguilla
- ◯ Antarctica
- ◯ Antigua and Barbuda
- ◯ Argentina
- ◯ Armenia
- ◯ Aruba
- ◯ Australia
- ◯ Austria
- ◯ Azerbaijan
- ◯ Bahamas
- ◯ Bahrain
- ◯ Bangladesh
- ◯ Barbados
- ◯ Belarus
- ◯ Belgium
- ◯ Belize
- ◯ Benin
- ◯ Bermuda
- ◯ Bhutan
- ◯ Bolivia (Plurinational State of)
- ◯ Bonaire, Sint Eustatius and Saba
- ◯ Bosnia and Herzegovina
- ◯ Botswana
- ◯ Bouvet Island
- ◯ Brazil
- ◯ British Indian Ocean Territory
- ◯ Brunei Darussalam
- ◯ Bulgaria
- ◯ Burkina Faso
- ◯ Burundi
- ◯ Cabo Verde
- ◯ Cambodia
- ◯ Cameroon
- ◯ Canada
- ◯ Cayman Islands
- ◯ Central African Republic
- ◯ Chad
- ◯ Chile
- ◯ China
- ◯ Christmas Island
- ◯ Cocos (Keeling) Islands
- ◯ Colombia
- ◯ Comoros
- ◯ Congo, Republic of the
- ◯ Congo, Democratic Republic of the
- ◯ Cook Islands
- ◯ Costa Rica
- ◯ Côte d'Ivoire
- ◯ Croatia
- ◯ Cuba
- ◯ Curaçao
- ◯ Cyprus
- ◯ Czechia
- ◯ Denmark
- ◯ Djibouti
- ◯ Dominica
- ◯ Dominican Republic
- ◯ Ecuador
- ◯ Egypt
- ◯ El Salvador
- ◯ Equatorial Guinea
- ◯ Eritrea

◯ Estonia
◯ Eswatini
◯ Ethiopia
◯ Falkland Islands (Malvinas)
◯ Faroe Islands
◯ Fiji
◯ Finland
◯ France
◯ French Guiana
◯ French Polynesia
◯ French Southern Territories
◯ Gabon
◯ Gambia
◯ Georgia
◯ Germany
◯ Ghana
◯ Gibraltar
◯ Greece
◯ Greenland
◯ Grenada
◯ Guadeloupe
◯ Guam
◯ Guatemala
◯ Guernsey
◯ Guinea
◯ Guinea-Bissau
◯ Guyana
◯ Haiti
◯ Heard Island and McDonald Islands
◯ Holy See
◯ Honduras
◯ Hong Kong
◯ Hungary
◯ Iceland
◯ India
◯ Indonesia
◯ Iran (Islamic Republic of)
◯ Iraq
◯ Ireland
◯ Isle of Man
◯ Israel
◯ Italy
◯ Jamaica
◯ Japan
◯ Jersey
◯ Jordan
◯ Kazakhstan
◯ Kenya
◯ Kiribati
◯ Korea (Democratic People's Republic of)
◯ Korea, Republic of
◯ Kuwait
◯ Kyrgyzstan
◯ Lao People's Democratic Republic
◯ Latvia
◯ Lebanon
◯ Lesotho
◯ Liberia
◯ Libya
◯ Liechtenstein
◯ Lithuania
◯ Luxembourg
◯ Macao
◯ Madagascar
◯ Malawi
◯ Malaysia
◯ Maldives
◯ Mali
◯ Malta
◯ Marshall Islands
◯ Martinique

◯ Mauritania
◯ Mauritius
◯ Mayotte
◯ Mexico
◯ Micronesia (Federated States of)
◯ Moldova, Republic of
◯ Monaco
◯ Mongolia
◯ Montenegro
◯ Montserrat
◯ Morocco
◯ Mozambique
◯ Myanmar
◯ Namibia
◯ Nauru
◯ Nepal
◯ Netherlands
◯ New Caledonia
◯ New Zealand
◯ Nicaragua
◯ Niger
◯ Nigeria
◯ Niue
◯ Norfolk Island
◯ North Macedonia
◯ Northern Mariana Islands
◯ Norway
◯ Oman
◯ Pakistan
◯ Palau
◯ Palestine, State of
◯ Panama
◯ Papua New Guinea
◯ Paraguay
◯ Peru
◯ Philippines
◯ Pitcairn
◯ Poland
◯ Portugal
◯ Puerto Rico
◯ Qatar
◯ Réunion
◯ Romania
◯ Russian Federation
◯ Rwanda
◯ Saint Barthélemy
◯ Saint Helena, Ascension and Tristan da Cunha
◯ Saint Kitts and Nevis
◯ Saint Lucia
◯ Saint Martin (French part)
◯ Saint Pierre and Miquelon
◯ Saint Vincent and the Grenadines
◯ Samoa
◯ San Marino
◯ Sao Tome and Principe
◯ Saudi Arabia
◯ Senegal
◯ Serbia
◯ Seychelles
◯ Sierra Leone
◯ Singapore
◯ Sint Maarten (Dutch part)
◯ Slovakia
◯ Slovenia
◯ Solomon Islands
◯ Somalia
◯ South Africa
◯ South Georgia and the South Sandwich Islands
◯ South Sudan
◯ Spain
◯ Sri Lanka

◯ Sudan
◯ Suriname
◯ Svalbard and Jan Mayen
◯ Sweden
◯ Switzerland
◯ Syrian Arab Republic
◯ Taiwan
◯ Tajikistan
◯ Tanzania, United Republic of
◯ Thailand
◯ Timor-Leste
◯ Togo
◯ Tokelau
◯ Tonga
◯ Trinidad and Tobago
◯ Tunisia
◯ Turkey
◯ Turkmenistan
◯ Turks and Caicos Islands
◯ Tuvalu
◯ Uganda
◯ Ukraine
◯ United Arab Emirates
◯ United Kingdom of Great Britain and Northern Ireland
◯ United States of America
◯ United States Minor Outlying Islands
◯ Uruguay
◯ Uzbekistan
◯ Vanuatu
◯ Venezuela (Bolivarian Republic of)
◯ Viet Nam
◯ Virgin Islands (British)
◯ Virgin Islands (U.S.)
◯ Wallis and Futuna
◯ Western Sahara
◯ Yemen
◯ Zambia
◯ Zimbabwe

(Start typing and the country will be suggested. If you only visited 1 country move to the next question)

Country 3 visited

◯ Afghanistan
◯ Åland Islands
◯ Albania
◯ Algeria
◯ American Samoa
◯ Andorra
◯ Angola
◯ Anguilla
◯ Antarctica
◯ Antigua and Barbuda
◯ Argentina
◯ Armenia
◯ Aruba
◯ Australia
◯ Austria
◯ Azerbaijan
◯ Bahamas
◯ Bahrain
◯ Bangladesh
◯ Barbados
◯ Belarus
◯ Belgium
◯ Belize
◯ Benin
◯ Bermuda
◯ Bhutan
◯ Bolivia (Plurinational State of)
◯ Bonaire, Sint Eustatius and Saba
◯ Bosnia and Herzegovina
◯ Botswana
◯ Bouvet Island
◯ Brazil
◯ British Indian Ocean Territory
◯ Brunei Darussalam
◯ Bulgaria
◯ Burkina Faso
◯ Burundi
◯ Cabo Verde
◯ Cambodia
◯ Cameroon
◯ Canada
◯ Cayman Islands
◯ Central African Republic
◯ Chad
◯ Chile
◯ China
◯ Christmas Island
◯ Cocos (Keeling) Islands
◯ Colombia
◯ Comoros
◯ Congo, Republic of the
◯ Congo, Democratic Republic of the
◯ Cook Islands
◯ Costa Rica
◯ Côte d'Ivoire
◯ Croatia
◯ Cuba
◯ Curaçao
◯ Cyprus
◯ Czechia
◯ Denmark
◯ Djibouti
◯ Dominica
◯ Dominican Republic
◯ Ecuador
◯ Egypt
◯ El Salvador
◯ Equatorial Guinea
◯ Eritrea

◯ Estonia
◯ Eswatini
◯ Ethiopia
◯ Falkland Islands (Malvinas)
◯ Faroe Islands
◯ Fiji
◯ Finland
◯ France
◯ French Guiana
◯ French Polynesia
◯ French Southern Territories
◯ Gabon
◯ Gambia
◯ Georgia
◯ Germany
◯ Ghana
◯ Gibraltar
◯ Greece
◯ Greenland
◯ Grenada
◯ Guadeloupe
◯ Guam
◯ Guatemala
◯ Guernsey
◯ Guinea
◯ Guinea-Bissau
◯ Guyana
◯ Haiti
◯ Heard Island and McDonald Islands
◯ Holy See
◯ Honduras
◯ Hong Kong
◯ Hungary
◯ Iceland
◯ India
◯ Indonesia
◯ Iran (Islamic Republic of)
◯ Iraq
◯ Ireland
◯ Isle of Man
◯ Israel
◯ Italy
◯ Jamaica
◯ Japan
◯ Jersey
◯ Jordan
◯ Kazakhstan
◯ Kenya
◯ Kiribati
◯ Korea (Democratic People's Republic of)
◯ Korea, Republic of
◯ Kuwait
◯ Kyrgyzstan
◯ Lao People's Democratic Republic
◯ Latvia
◯ Lebanon
◯ Lesotho
◯ Liberia
◯ Libya
◯ Liechtenstein
◯ Lithuania
◯ Luxembourg
◯ Macao
◯ Madagascar
◯ Malawi
◯ Malaysia
◯ Maldives
◯ Mali
◯ Malta
◯ Marshall Islands
◯ Martinique

◯ Mauritania
◯ Mauritius
◯ Mayotte
◯ Mexico
◯ Micronesia (Federated States of)
◯ Moldova, Republic of
◯ Monaco
◯ Mongolia
◯ Montenegro
◯ Montserrat
◯ Morocco
◯ Mozambique
◯ Myanmar
◯ Namibia
◯ Nauru
◯ Nepal
◯ Netherlands
◯ New Caledonia
◯ New Zealand
◯ Nicaragua
◯ Niger
◯ Nigeria
◯ Niue
◯ Norfolk Island
◯ North Macedonia
◯ Northern Mariana Islands
◯ Norway
◯ Oman
◯ Pakistan
◯ Palau
◯ Palestine, State of
◯ Panama
◯ Papua New Guinea
◯ Paraguay
◯ Peru
◯ Philippines
◯ Pitcairn
◯ Poland
◯ Portugal
◯ Puerto Rico
◯ Qatar
◯ Réunion
◯ Romania
◯ Russian Federation
◯ Rwanda
◯ Saint Barthélemy
◯ Saint Helena, Ascension and Tristan da Cunha
◯ Saint Kitts and Nevis
◯ Saint Lucia
◯ Saint Martin (French part)
◯ Saint Pierre and Miquelon
◯ Saint Vincent and the Grenadines
◯ Samoa
◯ San Marino
◯ Sao Tome and Principe
◯ Saudi Arabia
◯ Senegal
◯ Serbia
◯ Seychelles
◯ Sierra Leone
◯ Singapore
◯ Sint Maarten (Dutch part)
◯ Slovakia
◯ Slovenia
◯ Solomon Islands
◯ Somalia
◯ South Africa
◯ South Georgia and the South Sandwich Islands
◯ South Sudan
◯ Spain
◯ Sri Lanka

◯ Sudan
◯ Suriname
◯ Svalbard and Jan Mayen
◯ Sweden
◯ Switzerland
◯ Syrian Arab Republic
◯ Taiwan
◯ Tajikistan
◯ Tanzania, United Republic of
◯ Thailand
◯ Timor-Leste
◯ Togo
◯ Tokelau
◯ Tonga
◯ Trinidad and Tobago
◯ Tunisia
◯ Turkey
◯ Turkmenistan
◯ Turks and Caicos Islands
◯ Tuvalu
◯ Uganda
◯ Ukraine
◯ United Arab Emirates
◯ United Kingdom of Great Britain and Northern Ireland
◯ United States of America
◯ United States Minor Outlying Islands
◯ Uruguay
◯ Uzbekistan
◯ Vanuatu
◯ Venezuela (Bolivarian Republic of)
◯ Viet Nam
◯ Virgin Islands (British)
◯ Virgin Islands (U.S.)
◯ Wallis and Futuna
◯ Western Sahara
◯ Yemen
◯ Zambia
◯ Zimbabwe

(Start typing and the country will be suggested. If you only visited 2 counties, move to the next question)

Country 4 visited	◯ Afghanistan<br>◯ Åland Islands<br>◯ Albania<br>◯ Algeria<br>◯ American Samoa<br>◯ Andorra<br>◯ Angola<br>◯ Anguilla<br>◯ Antarctica<br>◯ Antigua and Barbuda<br>◯ Argentina<br>◯ Armenia<br>◯ Aruba<br>◯ Australia<br>◯ Austria<br>◯ Azerbaijan<br>◯ Bahamas<br>◯ Bahrain<br>◯ Bangladesh<br>◯ Barbados<br>◯ Belarus<br>◯ Belgium<br>◯ Belize<br>◯ Benin<br>◯ Bermuda<br>◯ Bhutan<br>◯ Bolivia (Plurinational State of)<br>◯ Bonaire, Sint Eustatius and Saba<br>◯ Bosnia and Herzegovina<br>◯ Botswana<br>◯ Bouvet Island<br>◯ Brazil<br>◯ British Indian Ocean Territory<br>◯ Brunei Darussalam<br>◯ Bulgaria<br>◯ Burkina Faso<br>◯ Burundi<br>◯ Cabo Verde<br>◯ Cambodia<br>◯ Cameroon<br>◯ Canada<br>◯ Cayman Islands<br>◯ Central African Republic<br>◯ Chad<br>◯ Chile<br>◯ China<br>◯ Christmas Island<br>◯ Cocos (Keeling) Islands<br>◯ Colombia<br>◯ Comoros<br>◯ Congo, Republic of the<br>◯ Congo, Democratic Republic of the<br>◯ Cook Islands<br>◯ Costa Rica<br>◯ Côte d'Ivoire<br>◯ Croatia<br>◯ Cuba<br>◯ Curaçao<br>◯ Cyprus<br>◯ Czechia<br>◯ Denmark<br>◯ Djibouti<br>◯ Dominica<br>◯ Dominican Republic<br>◯ Ecuador<br>◯ Egypt<br>◯ El Salvador<br>◯ Equatorial Guinea<br>◯ Eritrea

◯ Estonia
◯ Eswatini
◯ Ethiopia
◯ Falkland Islands (Malvinas)
◯ Faroe Islands
◯ Fiji
◯ Finland
◯ France
◯ French Guiana
◯ French Polynesia
◯ French Southern Territories
◯ Gabon
◯ Gambia
◯ Georgia
◯ Germany
◯ Ghana
◯ Gibraltar
◯ Greece
◯ Greenland
◯ Grenada
◯ Guadeloupe
◯ Guam
◯ Guatemala
◯ Guernsey
◯ Guinea
◯ Guinea-Bissau
◯ Guyana
◯ Haiti
◯ Heard Island and McDonald Islands
◯ Holy See
◯ Honduras
◯ Hong Kong
◯ Hungary
◯ Iceland
◯ India
◯ Indonesia
◯ Iran (Islamic Republic of)
◯ Iraq
◯ Ireland
◯ Isle of Man
◯ Israel
◯ Italy
◯ Jamaica
◯ Japan
◯ Jersey
◯ Jordan
◯ Kazakhstan
◯ Kenya
◯ Kiribati
◯ Korea (Democratic People's Republic of)
◯ Korea, Republic of
◯ Kuwait
◯ Kyrgyzstan
◯ Lao People's Democratic Republic
◯ Latvia
◯ Lebanon
◯ Lesotho
◯ Liberia
◯ Libya
◯ Liechtenstein
◯ Lithuania
◯ Luxembourg
◯ Macao
◯ Madagascar
◯ Malawi
◯ Malaysia
◯ Maldives
◯ Mali
◯ Malta
◯ Marshall Islands
◯ Martinique

◯ Mauritania
◯ Mauritius
◯ Mayotte
◯ Mexico
◯ Micronesia (Federated States of)
◯ Moldova, Republic of
◯ Monaco
◯ Mongolia
◯ Montenegro
◯ Montserrat
◯ Morocco
◯ Mozambique
◯ Myanmar
◯ Namibia
◯ Nauru
◯ Nepal
◯ Netherlands
◯ New Caledonia
◯ New Zealand
◯ Nicaragua
◯ Niger
◯ Nigeria
◯ Niue
◯ Norfolk Island
◯ North Macedonia
◯ Northern Mariana Islands
◯ Norway
◯ Oman
◯ Pakistan
◯ Palau
◯ Palestine, State of
◯ Panama
◯ Papua New Guinea
◯ Paraguay
◯ Peru
◯ Philippines
◯ Pitcairn
◯ Poland
◯ Portugal
◯ Puerto Rico
◯ Qatar
◯ Réunion
◯ Romania
◯ Russian Federation
◯ Rwanda
◯ Saint Barthélemy
◯ Saint Helena, Ascension and Tristan da Cunha
◯ Saint Kitts and Nevis
◯ Saint Lucia
◯ Saint Martin (French part)
◯ Saint Pierre and Miquelon
◯ Saint Vincent and the Grenadines
◯ Samoa
◯ San Marino
◯ Sao Tome and Principe
◯ Saudi Arabia
◯ Senegal
◯ Serbia
◯ Seychelles
◯ Sierra Leone
◯ Singapore
◯ Sint Maarten (Dutch part)
◯ Slovakia
◯ Slovenia
◯ Solomon Islands
◯ Somalia
◯ South Africa
◯ South Georgia and the South Sandwich Islands
◯ South Sudan
◯ Spain
◯ Sri Lanka

◯ Sudan
◯ Suriname
◯ Svalbard and Jan Mayen
◯ Sweden
◯ Switzerland
◯ Syrian Arab Republic
◯ Taiwan
◯ Tajikistan
◯ Tanzania, United Republic of
◯ Thailand
◯ Timor-Leste
◯ Togo
◯ Tokelau
◯ Tonga
◯ Trinidad and Tobago
◯ Tunisia
◯ Turkey
◯ Turkmenistan
◯ Turks and Caicos Islands
◯ Tuvalu
◯ Uganda
◯ Ukraine
◯ United Arab Emirates
◯ United Kingdom of Great Britain and Northern Ireland
◯ United States of America
◯ United States Minor Outlying Islands
◯ Uruguay
◯ Uzbekistan
◯ Vanuatu
◯ Venezuela (Bolivarian Republic of)
◯ Viet Nam
◯ Virgin Islands (British)
◯ Virgin Islands (U.S.)
◯ Wallis and Futuna
◯ Western Sahara
◯ Yemen
◯ Zambia
◯ Zimbabwe

(Start typing and the country will be suggested. If you only visited 3 counties, move to the next question)

Country 5 visited

- ◯ Afghanistan
- ◯ Åland Islands
- ◯ Albania
- ◯ Algeria
- ◯ American Samoa
- ◯ Andorra
- ◯ Angola
- ◯ Anguilla
- ◯ Antarctica
- ◯ Antigua and Barbuda
- ◯ Argentina
- ◯ Armenia
- ◯ Aruba
- ◯ Australia
- ◯ Austria
- ◯ Azerbaijan
- ◯ Bahamas
- ◯ Bahrain
- ◯ Bangladesh
- ◯ Barbados
- ◯ Belarus
- ◯ Belgium
- ◯ Belize
- ◯ Benin
- ◯ Bermuda
- ◯ Bhutan
- ◯ Bolivia (Plurinational State of)
- ◯ Bonaire, Sint Eustatius and Saba
- ◯ Bosnia and Herzegovina
- ◯ Botswana
- ◯ Bouvet Island
- ◯ Brazil
- ◯ British Indian Ocean Territory
- ◯ Brunei Darussalam
- ◯ Bulgaria
- ◯ Burkina Faso
- ◯ Burundi
- ◯ Cabo Verde
- ◯ Cambodia
- ◯ Cameroon
- ◯ Canada
- ◯ Cayman Islands
- ◯ Central African Republic
- ◯ Chad
- ◯ Chile
- ◯ China
- ◯ Christmas Island
- ◯ Cocos (Keeling) Islands
- ◯ Colombia
- ◯ Comoros
- ◯ Congo, Republic of the
- ◯ Congo, Democratic Republic of the
- ◯ Cook Islands
- ◯ Costa Rica
- ◯ Côte d'Ivoire
- ◯ Croatia
- ◯ Cuba
- ◯ Curaçao
- ◯ Cyprus
- ◯ Czechia
- ◯ Denmark
- ◯ Djibouti
- ◯ Dominica
- ◯ Dominican Republic
- ◯ Ecuador
- ◯ Egypt
- ◯ El Salvador
- ◯ Equatorial Guinea
- ◯ Eritrea

◯ Estonia
◯ Eswatini
◯ Ethiopia
◯ Falkland Islands (Malvinas)
◯ Faroe Islands
◯ Fiji
◯ Finland
◯ France
◯ French Guiana
◯ French Polynesia
◯ French Southern Territories
◯ Gabon
◯ Gambia
◯ Georgia
◯ Germany
◯ Ghana
◯ Gibraltar
◯ Greece
◯ Greenland
◯ Grenada
◯ Guadeloupe
◯ Guam
◯ Guatemala
◯ Guernsey
◯ Guinea
◯ Guinea-Bissau
◯ Guyana
◯ Haiti
◯ Heard Island and McDonald Islands
◯ Holy See
◯ Honduras
◯ Hong Kong
◯ Hungary
◯ Iceland
◯ India
◯ Indonesia
◯ Iran (Islamic Republic of)
◯ Iraq
◯ Ireland
◯ Isle of Man
◯ Israel
◯ Italy
◯ Jamaica
◯ Japan
◯ Jersey
◯ Jordan
◯ Kazakhstan
◯ Kenya
◯ Kiribati
◯ Korea (Democratic People's Republic of)
◯ Korea, Republic of
◯ Kuwait
◯ Kyrgyzstan
◯ Lao People's Democratic Republic
◯ Latvia
◯ Lebanon
◯ Lesotho
◯ Liberia
◯ Libya
◯ Liechtenstein
◯ Lithuania
◯ Luxembourg
◯ Macao
◯ Madagascar
◯ Malawi
◯ Malaysia
◯ Maldives
◯ Mali
◯ Malta
◯ Marshall Islands
◯ Martinique

◯ Mauritania
◯ Mauritius
◯ Mayotte
◯ Mexico
◯ Micronesia (Federated States of)
◯ Moldova, Republic of
◯ Monaco
◯ Mongolia
◯ Montenegro
◯ Montserrat
◯ Morocco
◯ Mozambique
◯ Myanmar
◯ Namibia
◯ Nauru
◯ Nepal
◯ Netherlands
◯ New Caledonia
◯ New Zealand
◯ Nicaragua
◯ Niger
◯ Nigeria
◯ Niue
◯ Norfolk Island
◯ North Macedonia
◯ Northern Mariana Islands
◯ Norway
◯ Oman
◯ Pakistan
◯ Palau
◯ Palestine, State of
◯ Panama
◯ Papua New Guinea
◯ Paraguay
◯ Peru
◯ Philippines
◯ Pitcairn
◯ Poland
◯ Portugal
◯ Puerto Rico
◯ Qatar
◯ Réunion
◯ Romania
◯ Russian Federation
◯ Rwanda
◯ Saint Barthélemy
◯ Saint Helena, Ascension and Tristan da Cunha
◯ Saint Kitts and Nevis
◯ Saint Lucia
◯ Saint Martin (French part)
◯ Saint Pierre and Miquelon
◯ Saint Vincent and the Grenadines
◯ Samoa
◯ San Marino
◯ Sao Tome and Principe
◯ Saudi Arabia
◯ Senegal
◯ Serbia
◯ Seychelles
◯ Sierra Leone
◯ Singapore
◯ Sint Maarten (Dutch part)
◯ Slovakia
◯ Slovenia
◯ Solomon Islands
◯ Somalia
◯ South Africa
◯ South Georgia and the South Sandwich Islands
◯ South Sudan
◯ Spain
◯ Sri Lanka

◯ Sudan
◯ Suriname
◯ Svalbard and Jan Mayen
◯ Sweden
◯ Switzerland
◯ Syrian Arab Republic
◯ Taiwan
◯ Tajikistan
◯ Tanzania, United Republic of
◯ Thailand
◯ Timor-Leste
◯ Togo
◯ Tokelau
◯ Tonga
◯ Trinidad and Tobago
◯ Tunisia
◯ Turkey
◯ Turkmenistan
◯ Turks and Caicos Islands
◯ Tuvalu
◯ Uganda
◯ Ukraine
◯ United Arab Emirates
◯ United Kingdom of Great Britain and Northern Ireland
◯ United States of America
◯ United States Minor Outlying Islands
◯ Uruguay
◯ Uzbekistan
◯ Vanuatu
◯ Venezuela (Bolivarian Republic of)
◯ Viet Nam
◯ Virgin Islands (British)
◯ Virgin Islands (U.S.)
◯ Wallis and Futuna
◯ Western Sahara
◯ Yemen
◯ Zambia
◯ Zimbabwe
(Start typing and the country will be suggested. If you only visited 4 counties, move to the next question)

Have you visited a state other than Washington since [enrollment_date]?

◯ Yes
◯ No - I have not traveled to a state outside of Washington

State 1

◯ Alabama
◯ Alaska
◯ Arizona
◯ Arkansas
◯ California
◯ Colorado
◯ Connecticut
◯ Delaware
◯ District of Columbia
◯ Florida
◯ Georgia
◯ Hawaii
◯ Idaho
◯ Illinois
◯ Indiana
◯ Iowa
◯ Kansas
◯ Kentucky
◯ Louisiana
◯ Maine
◯ Maryland
◯ Massachusetts
◯ Michigan
◯ Minnesota
◯ Mississippi
◯ Missouri
◯ Montana
◯ Nebraska
◯ Nevada
◯ New Hampshire
◯ New Jersey
◯ New Mexico
◯ New York
◯ North Carolina
◯ North Dakota
◯ Ohio
◯ Oklahoma
◯ Oregon
◯ Pennsylvania
◯ Rhode Island
◯ South Carolina
◯ South Dakota
◯ Tennessee
◯ Texas
◯ Utah
◯ Vermont
◯ Virginia
◯ Washington
◯ West Virginia
◯ Wisconsin
◯ Wyoming
◯ American Samoa
◯ Guam
◯ Northern Mariana Islands
◯ Puerto Rico
◯ U.S. Minor Outlying Islands
◯ U.S. Virgin Islands
(Start typing and the state will be suggested.)

State 2

◯ Alabama
◯ Alaska
◯ Arizona
◯ Arkansas
◯ California
◯ Colorado
◯ Connecticut
◯ Delaware
◯ District of Columbia
◯ Florida
◯ Georgia
◯ Hawaii
◯ Idaho
◯ Illinois
◯ Indiana
◯ Iowa
◯ Kansas
◯ Kentucky
◯ Louisiana
◯ Maine
◯ Maryland
◯ Massachusetts
◯ Michigan
◯ Minnesota
◯ Mississippi
◯ Missouri
◯ Montana
◯ Nebraska
◯ Nevada
◯ New Hampshire
◯ New Jersey
◯ New Mexico
◯ New York
◯ North Carolina
◯ North Dakota
◯ Ohio
◯ Oklahoma
◯ Oregon
◯ Pennsylvania
◯ Rhode Island
◯ South Carolina
◯ South Dakota
◯ Tennessee
◯ Texas
◯ Utah
◯ Vermont
◯ Virginia
◯ Washington
◯ West Virginia
◯ Wisconsin
◯ Wyoming
◯ American Samoa
◯ Guam
◯ Northern Mariana Islands
◯ Puerto Rico
◯ U.S. Minor Outlying Islands
◯ U.S. Virgin Islands

(Start typing and the state will be suggested. If you only visited 1 state move to the next question.)

State 3

- ◯ Alabama
- ◯ Alaska
- ◯ Arizona
- ◯ Arkansas
- ◯ California
- ◯ Colorado
- ◯ Connecticut
- ◯ Delaware
- ◯ District of Columbia
- ◯ Florida
- ◯ Georgia
- ◯ Hawaii
- ◯ Idaho
- ◯ Illinois
- ◯ Indiana
- ◯ Iowa
- ◯ Kansas
- ◯ Kentucky
- ◯ Louisiana
- ◯ Maine
- ◯ Maryland
- ◯ Massachusetts
- ◯ Michigan
- ◯ Minnesota
- ◯ Mississippi
- ◯ Missouri
- ◯ Montana
- ◯ Nebraska
- ◯ Nevada
- ◯ New Hampshire
- ◯ New Jersey
- ◯ New Mexico
- ◯ New York
- ◯ North Carolina
- ◯ North Dakota
- ◯ Ohio
- ◯ Oklahoma
- ◯ Oregon
- ◯ Pennsylvania
- ◯ Rhode Island
- ◯ South Carolina
- ◯ South Dakota
- ◯ Tennessee
- ◯ Texas
- ◯ Utah
- ◯ Vermont
- ◯ Virginia
- ◯ Washington
- ◯ West Virginia
- ◯ Wisconsin
- ◯ Wyoming
- ◯ American Samoa
- ◯ Guam
- ◯ Northern Mariana Islands
- ◯ Puerto Rico
- ◯ U.S. Minor Outlying Islands
- ◯ U.S. Virgin Islands

(Start typing and the state will be suggested. If you only visited 2 states, move to the next question.)

State 4

- ◯ Alabama
- ◯ Alaska
- ◯ Arizona
- ◯ Arkansas
- ◯ California
- ◯ Colorado
- ◯ Connecticut
- ◯ Delaware
- ◯ District of Columbia
- ◯ Florida
- ◯ Georgia
- ◯ Hawaii
- ◯ Idaho
- ◯ Illinois
- ◯ Indiana
- ◯ Iowa
- ◯ Kansas
- ◯ Kentucky
- ◯ Louisiana
- ◯ Maine
- ◯ Maryland
- ◯ Massachusetts
- ◯ Michigan
- ◯ Minnesota
- ◯ Mississippi
- ◯ Missouri
- ◯ Montana
- ◯ Nebraska
- ◯ Nevada
- ◯ New Hampshire
- ◯ New Jersey
- ◯ New Mexico
- ◯ New York
- ◯ North Carolina
- ◯ North Dakota
- ◯ Ohio
- ◯ Oklahoma
- ◯ Oregon
- ◯ Pennsylvania
- ◯ Rhode Island
- ◯ South Carolina
- ◯ South Dakota
- ◯ Tennessee
- ◯ Texas
- ◯ Utah
- ◯ Vermont
- ◯ Virginia
- ◯ Washington
- ◯ West Virginia
- ◯ Wisconsin
- ◯ Wyoming
- ◯ American Samoa
- ◯ Guam
- ◯ Northern Mariana Islands
- ◯ Puerto Rico
- ◯ U.S. Minor Outlying Islands
- ◯ U.S. Virgin Islands

(Start typing and the state will be suggested. If you only visited 3 states, move to the next question.)

State 5

◯ Alabama
◯ Alaska
◯ Arizona
◯ Arkansas
◯ California
◯ Colorado
◯ Connecticut
◯ Delaware
◯ District of Columbia
◯ Florida
◯ Georgia
◯ Hawaii
◯ Idaho
◯ Illinois
◯ Indiana
◯ Iowa
◯ Kansas
◯ Kentucky
◯ Louisiana
◯ Maine
◯ Maryland
◯ Massachusetts
◯ Michigan
◯ Minnesota
◯ Mississippi
◯ Missouri
◯ Montana
◯ Nebraska
◯ Nevada
◯ New Hampshire
◯ New Jersey
◯ New Mexico
◯ New York
◯ North Carolina
◯ North Dakota
◯ Ohio
◯ Oklahoma
◯ Oregon
◯ Pennsylvania
◯ Rhode Island
◯ South Carolina
◯ South Dakota
◯ Tennessee
◯ Texas
◯ Utah
◯ Vermont
◯ Virginia
◯ Washington
◯ West Virginia
◯ Wisconsin
◯ Wyoming
◯ American Samoa
◯ Guam
◯ Northern Mariana Islands
◯ Puerto Rico
◯ U.S. Minor Outlying Islands
◯ U.S. Virgin Islands

(Start typing and the state will be suggested. If you only visited 4 states, move to the next question.)

What was the purpose of your recent travel?	☐ Tourism<br>☐ Business<br>☐ Visiting family and/or friends<br>☐ I just moved here from another US state<br>☐ I just moved here from another country<br>☐ Other
When your recent illness was at its worst, how did it affect your ability to do your regular activities (work, school, etc.)?	◯ Not at all<br>◯ A little bit<br>◯ Somewhat<br>◯ Quite a bit<br>◯ Very much
Were any of the following activities impacted because you were feeling sick? Select all that apply.	☐ Running errands<br>☐ Exercising<br>☐ Socializing<br>☐ Volunteering<br>☐ Ability to take care of myself and/or family<br>☐ None of the Above/ My activities have not been impacted
Were any of the following activities impacted because you were feeling sick? Select all that apply.	☐ School<br>☐ Work<br>☐ Running errands<br>☐ Exercising<br>☐ Socializing<br>☐ Volunteering<br>☐ Ability to take care of myself and/or family<br>☐ None of the above/ my activities have not been impacted
Did your illness keep you from doing any of the following? Select all that apply.	☐ Attending class<br>☐ Going to work<br>☐ Studying<br>☐ Performing well on an exam or written assignment<br>☐ None of the Above/ My activities have not been impacted
How many days were you not able to go to school?	______________________
Did any of the following occur because you were feeling sick? Select all that apply.	☐ I missed work<br>☐ I worked from home<br>☐ I worked fewer hours than usual<br>☐ None of the above
How many days were you not able to go to work?	______________________
Did you seek clinical care for your illness since your symptoms were reported on [enrollment_date]?	◯ Yes - Doctor's office or Urgent Care<br>◯ Yes - Pharmacy (drugstore)<br>◯ Yes - Hospital or Emergency Department<br>◯ Yes - Other<br>◯ No

Did you receive an antiviral medication for your illness?	◯ No<br>◯ Yes; Oseltamivir (Tamiflu)<br>◯ Yes; Zanamivir (Relenza)<br>◯ Yes; Peramivir (Rapivab)<br>◯ Yes; Baloxavir (Xofluza)<br>◯ Yes, but I don't know which medication<br>◯ Do not know
Did you receive antibiotics for your illness?	◯ No<br>◯ Yes; Zithromycin (Z-pack or Zithromax)<br>◯ Yes; Amoxicillin (Moxatag)<br>◯ Yes; Amoxicillin/Clavulanate (Augmentin)<br>◯ Yes; Levofloxacin (Levaquin)<br>◯ Yes; Moxifloxacin (Avelox)<br>◯ Yes, but I don't remember which antibiotic<br>◯ Yes, but my antibiotic is not listed<br>◯ Do not know
Did you do any of the following because you enrolled in the Seattle Flu Study on [consent_date]? Select all that apply.	☐ Washed hands/used hand sanitizer more frequently<br>☐ Avoided public transport<br>☐ Stayed home<br>☐ Wore a face mask<br>☐ Encouraged others to get the flu vaccine<br>☐ Avoided contact with others<br>☐ Other
Did you do any of the following in the past week because you were feeling sick? Select all that apply.	☐ More frequently washed hands with soap and water or used hand sanitizer more frequently than usual<br>☐ More frequently covered my mouth and nose with a tissue when coughing or sneezing<br>☐ More frequently coughed or sneezed into my elbow or shoulder<br>☐ More frequently cleaned or disinfected my living space or workspace<br>☐ Avoided public transport or took it less often than I normally would<br>☐ Stayed home<br>☐ Wore a face mask in public to help protect others from getting sick<br>☐ Encouraged others to get the flu vaccine<br>☐ Avoided contact with others<br>☐ I did not change any of my behaviors because I was feeling sick
Is there anything else about your Seattle Flu Study experience that you'd like to share with us?	______________________________
What kind of gift card would you like?<br><br>You will receive a claim code for a $10 gift card within 1 month of us receiving your sample.	◯ Amazon<br>◯ Starbucks
